# Supplementary material for: Lineage frequency time series reveal elevated levels of genetic drift in SARS-CoV-2 transmission in England
Source: PLoS Pathog. 2024 Apr 15;20(4):e1012090. doi: 10.1371/journal.ppat.1012090 (PMC11045146; doi:10.1371/journal.ppat.1012090)
Supplement: S1 Appendix — (PDF) [file ppat.1012090.s002.pdf]

S1 Appendix

Summary of existing methods for inferring the strength of genetic drift

There are currently four main types of methods for estimating the strength of genetic drift in pathogen transmission, which we summarize here for giving context to this study.

1. **Contact tracing** can directly measure superspreading by following the close contacts of infected individuals to measure the distribution of the number of secondary cases (the offspring number distribution) [1]. However, some secondary cases may be missed which can lead to measurement bias [2]. Additionally, it is challenging to trace multiple generations of transmission, so we miss important information on host contact network structure.

2. Another type of method fits disease prevalence over time to **branching process models** [3]. These models assume a particular distribution for the offspring number distribution (often a negative binomial distribution) and estimate the combination of parameters of the offspring number distribution along with growth rate that best fit the observed disease prevalence. External information about the growth rate can be used to constrain the parameters of the offspring number distribution.

3. **Phylogenetics** methods arrange genomics sequences into a tree based on genomic distance and either measure the distribution of lineage sizes (number of sequences in different parts of the tree) [4] or fit the rate at which branches in the tree coalescence to determine the effective population size [5, 6, 7, 8]. The effective population size is the population size that would reproduce the observed population dynamics under the idealized conditions of Wright-Fisher dynamics (discrete non- overlapping generations, a constant population size, and offspring determined by sampling with replacement from the previous generation). In neutral populations, a lower effective population size indicates a higher level of genetic drift.

4. **Time series frequency methods** make use of a signature that genetic drift leaves in time series data, which is that it causes fluctuations in the lineage abundances. Higher amounts of genetic drift (lower effective population size) lead to larger fluctuations, and the magnitude of the fluctuations can be fit to determine the effective population size [9, 10] (Fig 1a). Time series methods have also been used extensively in population genetics [11, 12, 13, 14, 15, 16] and to estimate within-host effective population size [17] and between-host transmission bottleneck sizes [18].

Comparison to SEIR null model

In the main text, we compared the inferred $\tilde{N}_e(t)$ to an SIR model. However, there are likely more complex epidemiological dynamics describing SARS-CoV-2. Here we check the results for an SEIR model which includes a susceptible, exposed, infectious, and recovered class. The SEIR model is a good representation of the epidemiology of SARS-CoV-2 when PCR test positivity is closely associated with an infected host being infectious; the literature suggests that this is a good assumption for SARS-CoV-2 [19], but we also test this assumption below. The exposed class thus represents individuals before they are infectious and test positive. $\tilde{N}_e(t)$ for an SEIR model in equilibrium (number of infectious individuals is constant over time) is given by (see Methods for derivation):

$$\tilde{N_e}^{\text{SEIR,eq}}(t) \equiv \{N_e(t)\tau(t)\}^{\text{SEIR,eq}} = \frac{(E(t)+I(t))^2}{2R_t\gamma_I(t)}. \quad (1)$$

where $E(t)$ is the number of exposed individuals, $I(t)$ is the number of infectious individuals, R_t is the effective reproduction number, and γ_I is the rate at which infectious individuals stop being infectious. While this equation is derived under equilibrium conditions, we show using simulations that this equation accurately estimates $\tilde{N}_e(t)$ in non-equilibrium conditions after the peak of the pandemic (S29 Fig); before the pandemic peak, this equation overestimates $\tilde{N}_e(t)$ but by less than one order of magnitude. Additionally, we show that calculating the $\tilde{N}_e(t)$ using the equation for an SIR model (Equation 1) when the dynamics are actually described by an SEIR model provides a lower bound on the actual $\tilde{N}_e(t)$. Thus, if the true dynamics of

SARS-CoV-2 in England are actually SEIR dynamics, then the inference results shown in Fig 3c using the SIR model should be an underestimate of the level of genetic drift; thus our main result that the literature values of superspreading do not sufficiently explain our results should still hold.

In reality, it may also be the case that some people test positive in a PCR test before they become infectious. To test the impact of this possibility on our results, in our simulations we recorded both exposed and infectious individuals as testing positive. We then calculated the SEIR model $\tilde{N}_e(t)$ numerically as described in "Calculating the effective population size for an SIR or SEIR model" assuming that $I(t)$ includes both infectious and exposed individuals (S30 Fig). We find that the numerical solutions give slightly higher $\tilde{N}_e(t)$ as compared with the true analytical solutions; however, the numerical solutions to the SEIR and SIR models bound the inferred $\tilde{N}_e(t)$. Thus we also expect that our main result that the literature values of superspreading do not sufficiently explain our results should still hold in this scenario.

To calculate the SEIR model $\tilde{N}_e(t)$ for the actual data, for the number of infectious individuals, we used the number of positive individuals estimated from the UK Office for National Statistics' COVID-19 Infection Survey [20], which is a household surveillance study that reports positive PCR tests, regardless of symptom status. We used the measured effective reproduction number in England reported by the UK Health Security Agency [21]. We found that $\tilde{N_e}^{\text{SEIR}}(t)$ is very similar to the number of positives because the effective reproduction number in England was very close to 1 across time. To calculate $\tilde{N_e}^{\text{SEIR}}(t)$ for each variant or group of lineages, we rescaled the population-level $I(t)$ and R_t based on the fraction of each variant in the population and the relative differences in reproduction numbers between variants (see Methods). We then calculated the scaled true population size, $\tilde{N}(t) \equiv N(t)\tau(t)$, for the SEIR model by multiplying by the variance in offspring number, σ^2, for the SEIR model [22]

$$\tilde{N}^{\text{SEIR}}(t) = \tilde{N_e}^{\text{SEIR}}(t)\{\sigma^2\}^{\text{SEIR}} \tag{2}$$

$$\{\sigma^2\}^{\text{SEIR}} = 2. \tag{3}$$

Overall, the inferred $\tilde{N}_e(t)$ is lower than $\tilde{N}^{\text{SEIR}}(t)$ by a time-dependent factor that varies between 70 and 2000 (S13 Fig), suggesting high levels of genetic drift in England across time, which is consistent with what we find with an SIR model (Figs 2 and S12). Also similarly to in the case with an SIR model, the ratio of $\tilde{N}^{\text{SEIR}}(t)$ to the inferred $\tilde{N}_e(t)$ for Alpha decreased over time, suggesting that the stochasticity in the transmission of Alpha decreased over time.

The effect of background selection on effective population size

We estimated the magnitude by which we expect the effective population size to be decreased due to background selection given the empirically estimated distribution of fitness effects using both simulations (described in the main text) and analytical theory (described here). Most studies on background selection consider strongly deleterious mutations with a single negative fitness value and assume that deleterious mutants quickly die out so that multiple mutations do not occur in the same background [23]. However, in this case we need to consider a distribution of fitness effects and the possibility of mutants with different fitnesses existing simultaneously. As such, we used Equation 8 derived from Ref. [24] for the effective population size in the presence of deleterious mutations with a distribution of fitness effects, assuming a constant mutation rate and no recombination

$$N_e \approx N\exp\Big[-\int_{\frac{1}{N}}^{\infty} \frac{\mu}{s}(1-e^{-st})^2\rho(s)ds\Big] \tag{4}$$

where μ is the deleterious mutation rate per generation per genome, $\rho(s)$ is the deleterious distribution of fitness effects (i.e. the fitness effect is $-s$), t is time in generations into the past, and N is the census population size. Assuming no recombination is a conservative assumption, as recombination mitigates the effects of background selection [23].

Using the empirically estimated distribution of fitness effects from Ref. [25] (which are consistent with experimental measurements, see Refs. [26, 27, 28, 29]) and the clock rate of 31 substitutions per year (Nextstrain SARS-CoV-2 GISAID build on August 7, 2023), a generation time of 5.1 days [30], and a population size of 10^4 (order of magnitude of true population size), we estimate that the effective population

size will be decreased by at most a factor of 2 at times far into the past, and less in more recent times (see S31 Fig). The above formula was derived assuming strong selection ($s \gg \frac{1}{N}$) for the bulk of deleterious mutations, which we see from the distribution of fitness effects does hold (S15 Fig). Thus, while background selection will in general decrease the effective population size, in this system it can only explain a small fraction of the observed reduction of two orders of magnitude. This result is consistent with what we found in the simulations (S18 Fig).

Application to COG-UK data by regions in England

The inference of effective population size can also reveal information about the well-mixed or spatially-structured nature of transmission dynamics within England. This can be done by inferring effective population size at smaller geographical scales within England. If the transmission dynamics were completely well-mixed, then we would expect $\tilde{N}_e(t)$ to be the same across regions and compared to England. On the other hand, if the transmission dynamics were completely spatially segregated (i.e. transmission only occurs within the defined geographical areas, but not between them) and the dynamics were the same in each region, we would expect that the ratio $\tilde{N}_e^{\text{SIR}}(t)/\tilde{N}_e^{\text{inf}}(t)$ to be the same across regions.

The geographical areas that we used were the 9 regions of England: East Midlands, East of England, London, North East, North West, South East, South West, West Midlands, and Yorkshire and The Humber. We looked at sequences from each region, repeating the analysis described above, and inferred the scaled effective population size (S19 Fig). We observe a lower $\tilde{N}_e(t)$ for in the region than in England for Delta in all regions, for Alpha in all regions except North East (where there was not enough data), and for B.1.177 in all regions except North East. For lineages pre-B.1.177, the inferred $\tilde{N}_e(t)$ is not significantly differnt in the region than in England. These results suggest that the dynamics are not well-mixed during the B.1.177, Alpha, and Delta waves.

The calculated SIR model $\tilde{N}_e^{\text{SIR}}(t)$ (S32 Fig) and the number of positive individuals in each region (S20 Fig) were 1-2 orders of magnitude higher than the inferred $\tilde{N}_e(t)$, suggesting high levels of genetic drift. The ratios of the SIR model $\tilde{N}_e(t)$ and the number of positives to the inferred $\tilde{N}_e(t)$ in the regions were similar to one another and to that seen in England as a whole, consistent with a scenario where the dynamics are spatially-structured and the extent of stochasticity in transmission is similar across regions.

Similarly to in England as a whole, the inferred measurement noise in each region was mostly indistinguishable from uniform sampling except for in a few timepoints (S33 Fig).

References

1. Lloyd-Smith JO, Schreiber SJ, Kopp PE, and Getz WM. Superspreading and the effect of individual variation on disease emergence. Nature 2005; 438:355–9
2. Adam D, Gostic K, Tsang T, Wu P, Lim WW, Yeung A, Wong J, Lau E, Du Z, Chen D, et al. Time-varying transmission heterogeneity of SARS and COVID-19 in Hong Kong (preprint). 2022
3. Endo A et al. Estimating the overdispersion in COVID-19 transmission using outbreak sizes outside China. Wellcome Open Research 2020; 5
4. Du Plessis L, McCrone JT, Zarebski AE, Hill V, Ruis C, Gutierrez B, Raghwani J, Ashworth J, Colquhoun R, Connor TR, et al. Establishment and lineage dynamics of the SARS-CoV-2 epidemic in the UK. Science 2021; 371:708–12
5. Stadler T. On incomplete sampling under birth–death models and connections to the sampling-based coalescent. Journal of Theoretical Biology 2009; 261:58–66
6. Pybus OG, Rambaut A, and Harvey PH. An integrated framework for the inference of viral population history from reconstructed genealogies. Genetics 2000; 155:1429–37
7. Volz EM and Didelot X. Modeling the growth and decline of pathogen effective population size provides insight into epidemic dynamics and drivers of antimicrobial resistance. Systematic Biology 2018; 67:719–28

8. Ho SY and Shapiro B. Skyline-plot methods for estimating demographic history from nucleotide sequences. Molecular Ecology Resources 2011; 11:423–34
9. Zinger T, Gelbart M, Miller D, Pennings PS, and Stern A. Inferring population genetics parameters of evolving viruses using time-series data. Virus Evolution 2019; 5:vez011
10. Levy SF, Blundell JR, Venkataram S, Petrov DA, Fisher DS, and Sherlock G. Quantitative evolutionary dynamics using high-resolution lineage tracking. Nature 2015; 519:181–6
11. Bollback JP, York TL, and Nielsen R. Estimation of $2N_e s$ from temporal allele frequency data. Genetics 2008; 179:497–502
12. Ferrer-Admetlla A, Leuenberger C, Jensen JD, and Wegmann D. An approximate Markov model for the Wright–Fisher diffusion and its application to time series data. Genetics 2016; 203:831–46
13. Feder AF, Kryazhimskiy S, and Plotkin JB. Identifying signatures of selection in genetic time series. Genetics 2014; 196:509–22
14. Buffalo V and Coop G. The linked selection signature of rapid adaptation in temporal genomic data. Genetics 2019; 213:1007–45
15. Steinrücken M, Bhaskar A, and Song YS. A novel spectral method for inferring general diploid selection from time series genetic data. The Annals of Applied Statistics 2014; 8:2203
16. Williamson EG and Slatkin M. Using maximum likelihood to estimate population size from temporal changes in allele frequencies. Genetics 1999; 152:755–61
17. Lumby CK, Zhao L, Breuer J, and Illingworth CJ. A large effective population size for established within-host influenza virus infection. eLife 2020; 9:e56915
18. Sobel Leonard A, Weissman DB, Greenbaum B, Ghedin E, and Koelle K. Transmission bottleneck size estimation from pathogen deep-sequencing data, with an application to human influenza A virus. Journal of Virology 2017; 91:e00171–17
19. Goyal A, Reeves DB, Cardozo-Ojeda EF, Schiffer JT, and Mayer BT. Viral load and contact heterogeneity predict SARS-CoV-2 transmission and super-spreading events. eLife 2021; 10:e63537
20. UK Office for National Statistics. Coronavirus (COVID-19) Infection Survey: England. `https://www.ons.gov.uk/peoplepopulationandcommunity/healthandsocialcare/conditionsanddiseases/datasets/coronaviruscovid19infectionsurveydata`. Accessed: 2021-12-10
21. UK Health Security Agency. The R value and growth rate. `https://www.gov.uk/guidance/the-r-value-and-growth-rate#:~:text=The%20R%20range%20for%20the,as%20of%2019%20March%202021.&text=The%20R%20range%20for%20the,as%20of%2012%20March%202021.`. Accessed: 2021-12-10
22. Koelle K and Rasmussen DA. Rates of coalescence for common epidemiological models at equilibrium. Journal of the Royal Society Interface 2012; 9:997–1007
23. Charlesworth B and Jensen JD. Effects of selection at linked sites on patterns of genetic variability. Annual Review of Ecology, Evolution, and Systematics 2021; 52:177–97
24. Nicolaisen LE and Desai MM. Distortions in genealogies due to purifying selection and recombination. Genetics 2013; 195:221–30
25. Bloom JD and Neher RA. Fitness effects of mutations to SARS-CoV-2 proteins. bioRxiv 2023 :2023–1
26. Dadonaite B, Crawford KH, Radford CE, Farrell AG, Timothy CY, Hannon WW, Zhou P, Andrabi R, Burton DR, Liu L, et al. A pseudovirus system enables deep mutational scanning of the full SARS-CoV-2 spike. Cell 2023; 186:1263–78
27. Starr TN, Greaney AJ, Stewart CM, Walls AC, Hannon WW, Veesler D, and Bloom JD. Deep mutational scans for ACE2 binding, RBD expression, and antibody escape in the SARS-CoV-2 Omicron BA. 1 and BA. 2 receptor-binding domains. PLoS Pathogens 2022; 18:e1010951
28. Iketani S, Hong SJ, Sheng J, Bahari F, Culbertson B, Atanaki FF, Aditham AK, Kratz AF, Luck MI, Tian R, et al. Functional map of SARS-CoV-2 3CL protease reveals tolerant and immutable sites. Cell Host & Microbe 2022; 30:1354–62

29. Flynn JM, Huang QYJ, Zvornicanin SN, Schneider-Nachum G, Shaqra AM, Yilmaz NK, Moquin SA, Dovala D, Schiffer CA, and Bolon DN. Systematic analyses of the resistance potential of drugs targeting SARS-CoV-2 main protease. ACS Infectious Diseases 2023; 9:1372–86

30. Hart WS, Abbott S, Endo A, Hellewell J, Miller E, Andrews N, Maini PK, Funk S, and Thompson RN. Inference of the SARS-CoV-2 generation time using UK household data. eLife 2022; 11:e70767
