# Supplementary material for: Lineage frequency time series reveal elevated levels of genetic drift in SARS-CoV-2 transmission in England
Source: PLoS Pathog. 2024 Apr 15;20(4):e1012090. doi: 10.1371/journal.ppat.1012090 (PMC11045146; doi:10.1371/journal.ppat.1012090)
Supplement: S1 Table — Any error intervals that are reported are taken from the reference (sometimes defined differently). The estimate taken from Ref. [57] assumes no self-isolation upon symptom onset and no testing; lifting these assumptions leads to similar or lower overdispersion. (PDF) [file ppat.1012090.s003.pdf]

**Date**	**Location**	**Method**	$\langle Z \rangle$	**Var(Z)**	k	**Reference**
February 23 to April 22 2020	Israel	Phylodynamics	2.5 (2, 3)	(65, 627.5)	(0.02, 0.1)	[1]
Beginning of pandemic to February 27 2020	Worldwide excluding China	Branching process model of number of imported and local cases	2.5 (,)	65 (33.75, 127.5)	0.1 (0.05, 0.2)	[2]
March 1 to May 3 2020	Georgia (USA)	Spatiotemporal transmission model fit to multiple data sources	2 (0.5, 3.5)	12.26 (0.88, 101.5)	0.39 (0.125, 0.65)	[3]
March 1 to November 1 2020	Denmark	Model fitting the case numbers across multiple regions	1.1 (0.8, 1.4)	12.1 (4.36, 25.9)	0.11 (0.08, 0.18)	[4]
Beginning of pandemic until January 18 2020	China (Wuhan)	Stochastic simulations fit to infected cases	2.2 (1.4, 3.8)	11.16 (1.68, 1035.2)	0.54 (0.014, 6.95)	[5]
August to September 2020	UK	Model using empirical viral load trajectories and contact numbers	1.21 (0.84, 2.51)	7.07 (2.65, 44.51)	0.25 (0.15, 0.39)	[6]
May 15 to August 1 2020	Tamil Nadu and Andhra Pradesh (India)	Contact tracing and incidence	1.25 (1.1, 1.4)	4.31 (3.43, 5.4)	0.51 (0.49, 0.52)	[7]
January to February 2021	UK	Model using empirical viral load trajectories and contact numbers	0.54 (0.4, 1.03)	1.42 (0.66, 9.19)	0.33 (0.13, 0.61)	[6]
January 23 to April 28 2020	Hong Kong	Contact tracing	0.58 (,)	1.36 (,)	0.43 (,)	[8]
January 16 to April 3 2020	Hunan (China)	Contact tracing	0.4 (0.35, 0.47)	0.93 (0.66, 1.43)	0.3 (0.23, 0.39)	[9]
January 14 to February 12 2020	Shenzhen (China)	Contact tracing	0.4 (0.3, 0.5)	0.68 (0.38, 1.21)	0.58 (0.35, 1.18)	[10]

S1 Table: Overdispersion values from the literature ordered from highest to lowest variance in offspring number. Any error intervals that are reported are taken from the reference (sometimes defined differently). The estimate taken from Ref. [6] assumes no self-isolation upon symptom onset and no testing; lifting these assumptions leads to similar or lower overdispersion.

References

1. Miller D, Martin MA, Harel N, Tirosh O, Kustin T, Meir M, Sorek N, Gefen-Halevi S, Amit S, Vorontsov O, et al. Full genome viral sequences inform patterns of SARS-CoV-2 spread into and within Israel. Nature Communications 2020; 11:1–10
2. Endo A et al. Estimating the overdispersion in COVID-19 transmission using outbreak sizes outside China. Wellcome Open Research 2020; 5
3. Lau MS, Grenfell B, Thomas M, Bryan M, Nelson K, and Lopman B. Characterizing superspreading events and age-specific infectiousness of SARS-CoV-2 transmission in Georgia, USA. Proceedings of the National Academy of Sciences 2020; 117:22430–5
4. Kirkegaard JB and Sneppen K. Variability of individual infectiousness derived from aggregate statistics of COVID-19. medRxiv 2021
5. Riou J and Althaus CL. Pattern of early human-to-human transmission of Wuhan 2019 novel coronavirus (2019-nCoV), December 2019 to January 2020. Eurosurveillance 2020; 25:2000058
6. Quilty BJ, Chapman LA, Wong KL, Gimma A, Pickering S, JD S, Neil RPG, Jarvis CI, and Kucharski AJ. Reconstructing the secondary case distribution of SARS-CoV-2 from heterogeneity in viral load trajectories and social contacts. Report for SPI-M-O and SAGE 2021
7. Laxminarayan R, Wahl B, Dudala SR, Gopal K, Mohan B C, Neelima S, Jawahar Reddy K, Radhakrishnan J, and Lewnard JA. Epidemiology and transmission dynamics of COVID-19 in two Indian states. Science 2020; 370:691–7
8. Adam DC, Wu P, Wong JY, Lau EH, Tsang TK, Cauchemez S, Leung GM, and Cowling BJ. Clustering and superspreading potential of SARS-CoV-2 infections in Hong Kong. Nature Medicine 2020; 26:1714–9
9. Sun K, Wang W, Gao L, Wang Y, Luo K, Ren L, Zhan Z, Chen X, Zhao S, Huang Y, et al. Transmission heterogeneities, kinetics, and controllability of SARS-CoV-2. Science 2021; 371:eabe2424
10. Bi Q, Wu Y, Mei S, Ye C, Zou X, Zhang Z, Liu X, Wei L, Truelove SA, Zhang T, et al. Epidemiology and transmission of COVID-19 in 391 cases and 1286 of their close contacts in Shenzhen, China: a retrospective cohort study. The Lancet Infectious Diseases 2020; 20:911–9
