# Supplementary figures and images for: Lineage frequency time series reveal elevated levels of genetic drift in SARS-CoV-2 transmission in England
Source: PLoS Pathog. 2024 Apr 15;20(4):e1012090. doi: 10.1371/journal.ppat.1012090 (PMC11045146; doi:10.1371/journal.ppat.1012090)

Fraction of simulations where  
95% CI included true value

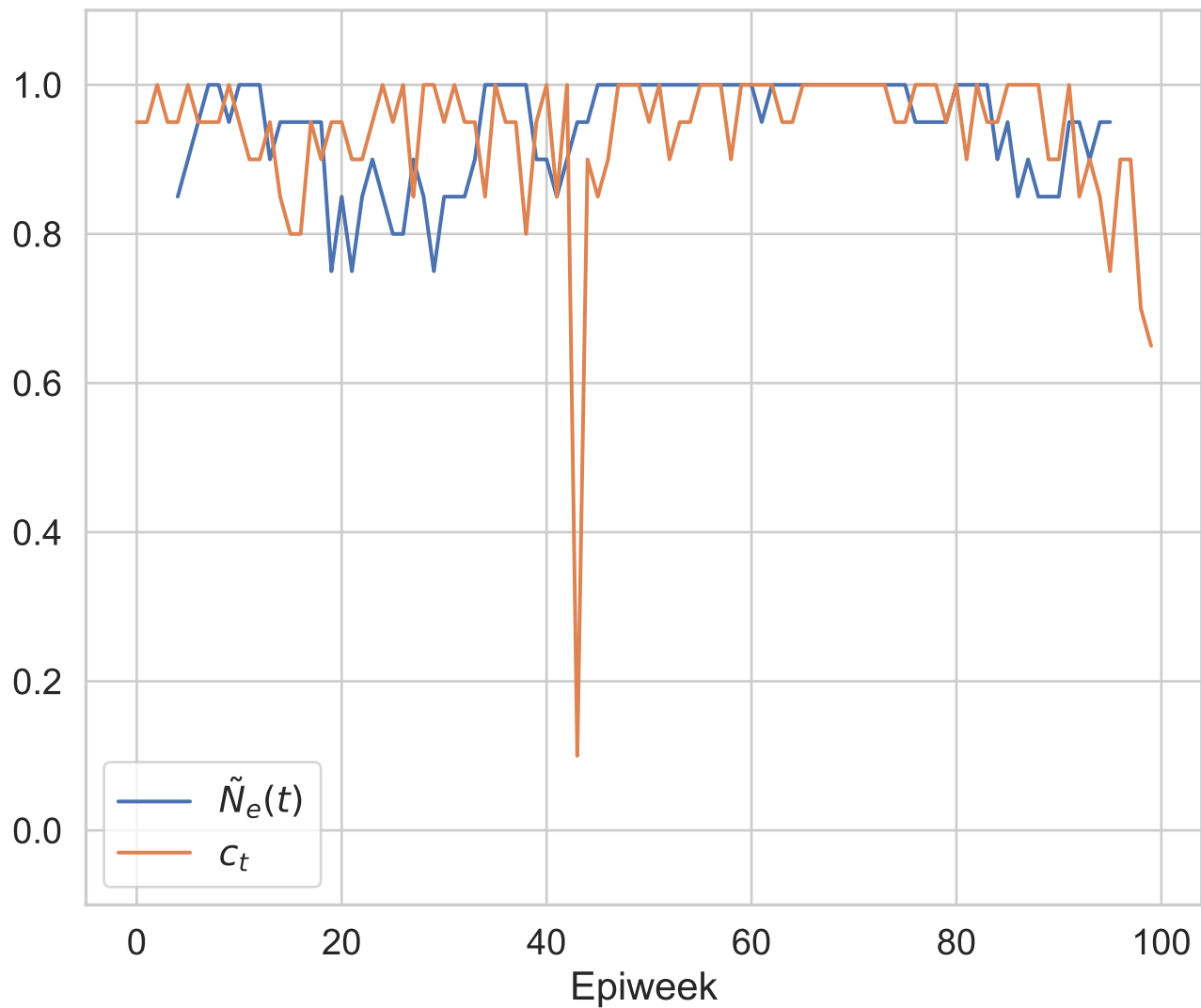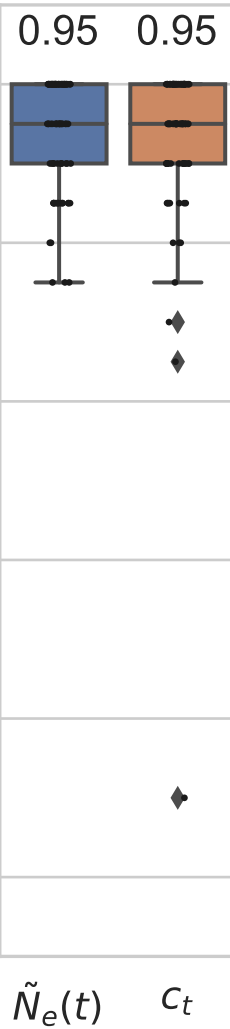

Supplement: S1 Fig — (Right) Boxes indicate the quartiles and the line inside the box (and number above) indicates the median. Whiskers indicate the extreme values excluding outliers. Simulation parameters are specified in the Methods and Fig 1, which shows a single simulation instance. For the inference, we created coarse-grained lineages randomly 20 times. (PDF) [file ppat.1012090.s004.pdf]

**a**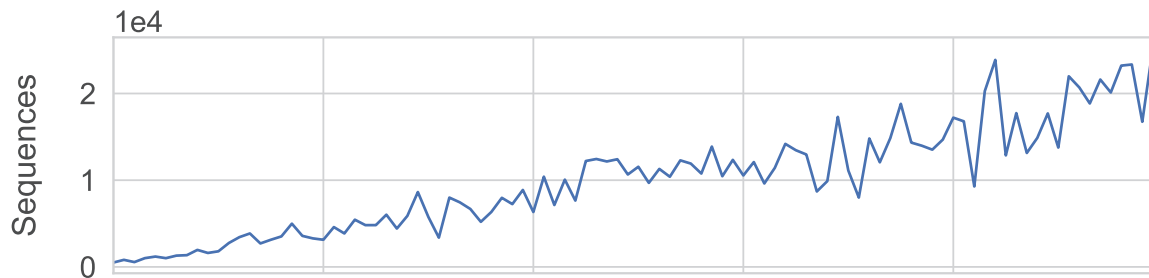**b**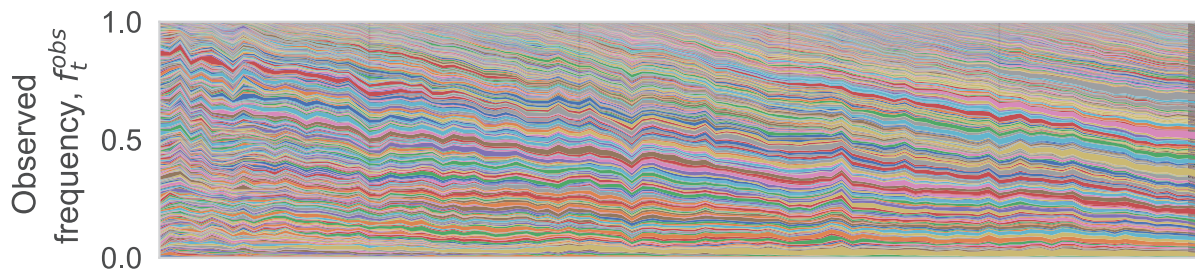**c**  
Scaled effective  
population size,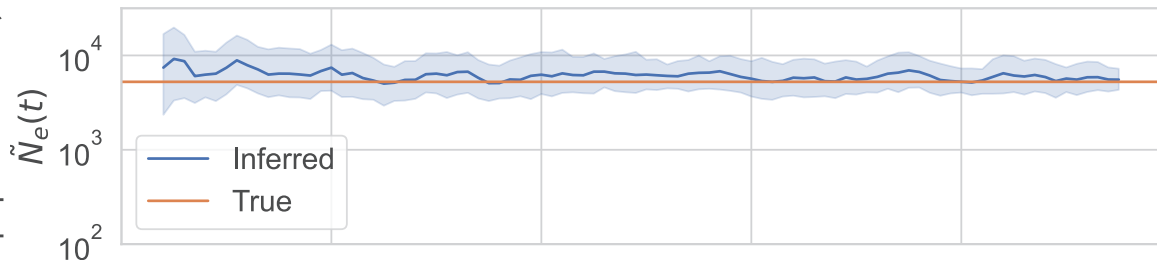**d**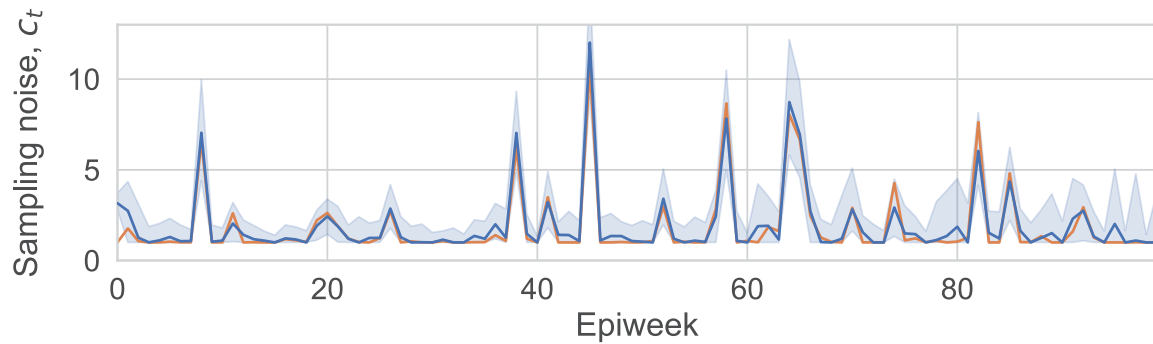

Supplement: S2 Fig — (a) Number of sequences sampled. (b) Simulated lineage frequency trajectories. (c) Inferred effective population size (Ne˜(t)) on simulated data compared to true values. (d) Inferred measurement noise (ct) on simulated data compared to true values. In (c) the shaded region shows the 95% confidence interval calculated using the posterior, and in (d) the shaded region shows the 95% confidence interval calculated using bootstrapping (see Methods). (PDF) [file ppat.1012090.s005.pdf]

**a**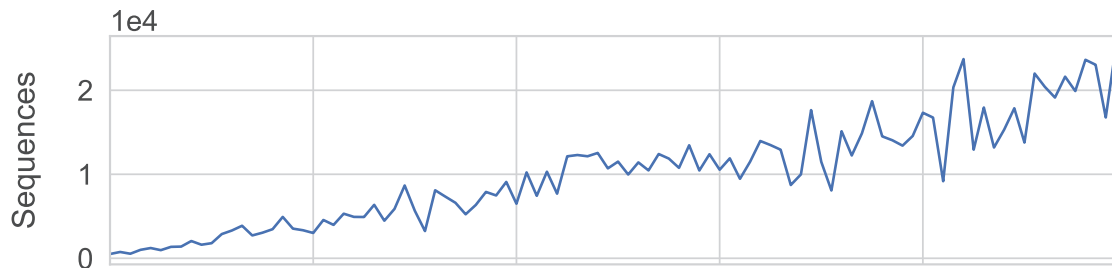**b**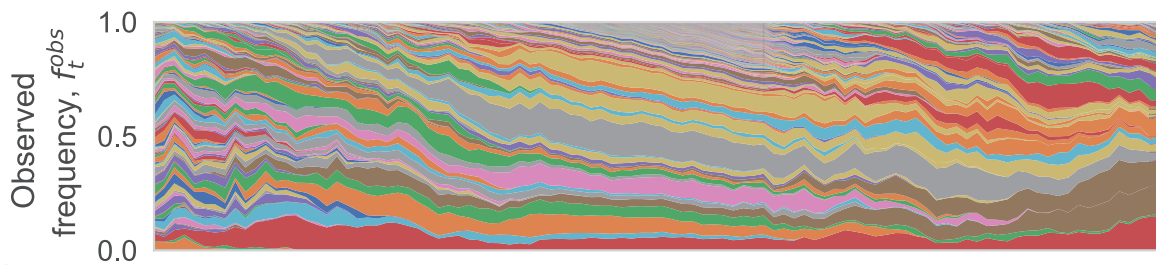**c**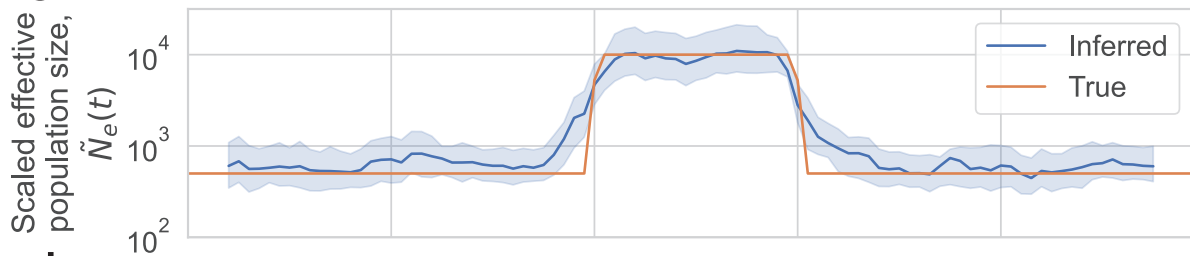**d**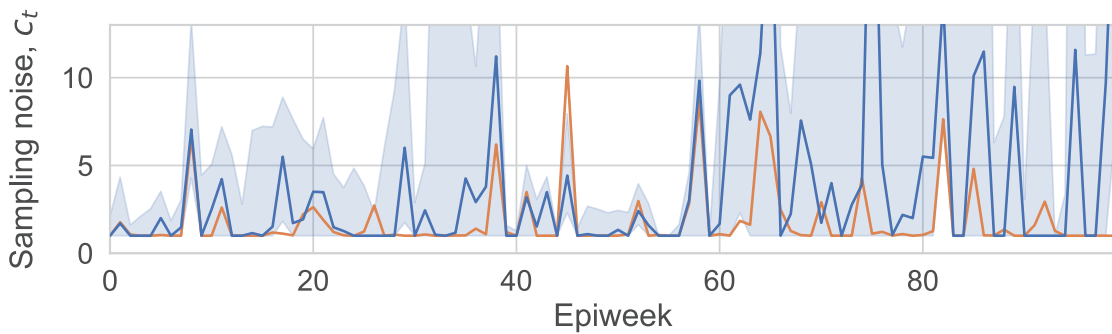

Supplement: S3 Fig — (a) Number of sequences sampled. (b) Simulated lineage frequency trajectories. (c) Inferred effective population size (Ne˜(t)) on simulated data compared to true values. (d) Inferred measurement noise (ct) on simulated data compared to true values. In (c) the shaded region shows the 95% confidence interval calculated using the posterior, and in (d) the shaded region shows the 95% confidence interval calculated using bootstrapping (see Methods). (PDF) [file ppat.1012090.s006.pdf]

pre-B.1.177

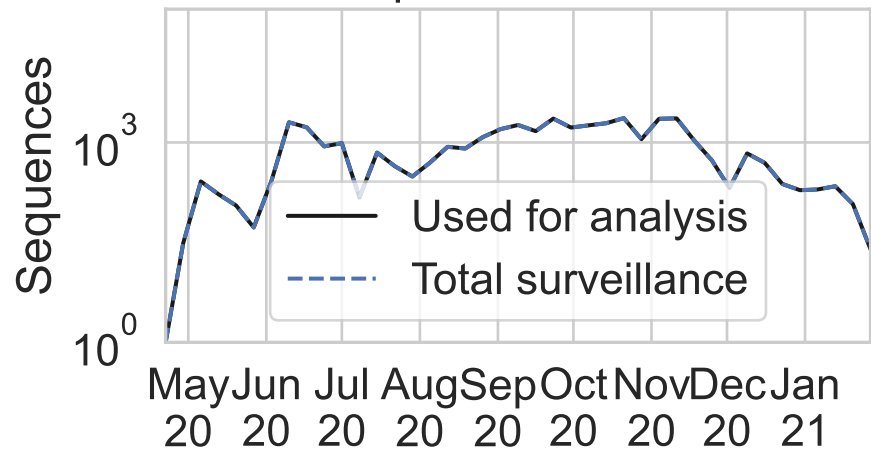

B.1.177

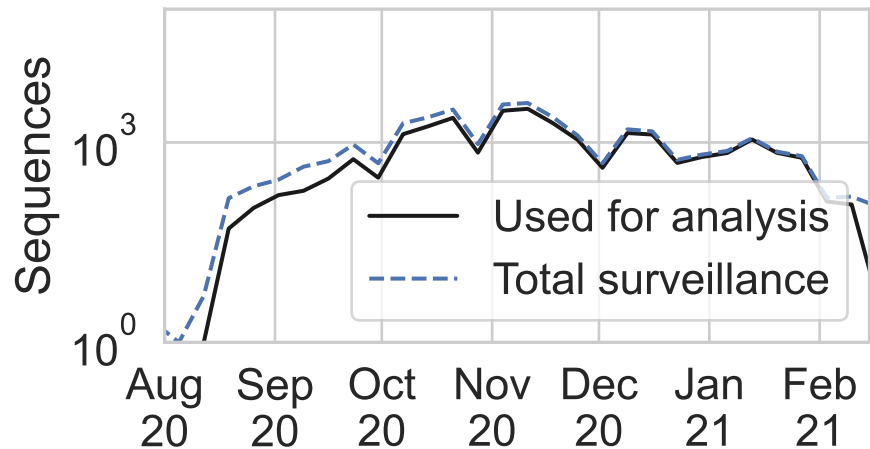

Alpha

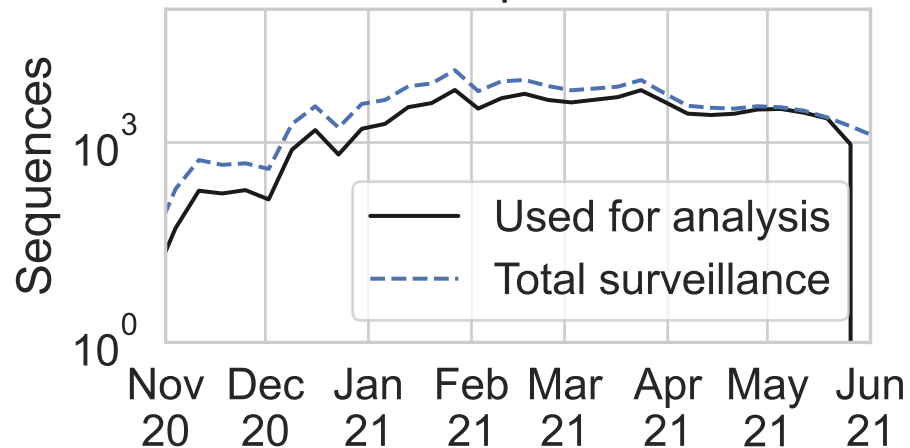

Delta

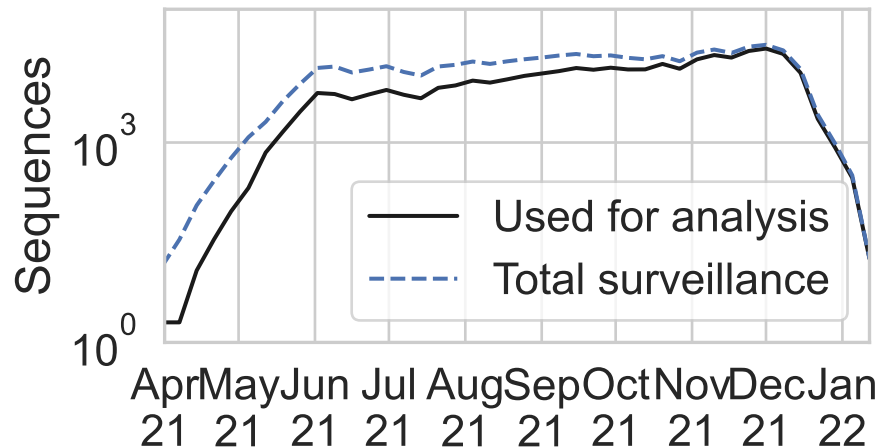

Supplement: S4 Fig — (PDF) [file ppat.1012090.s007.pdf]

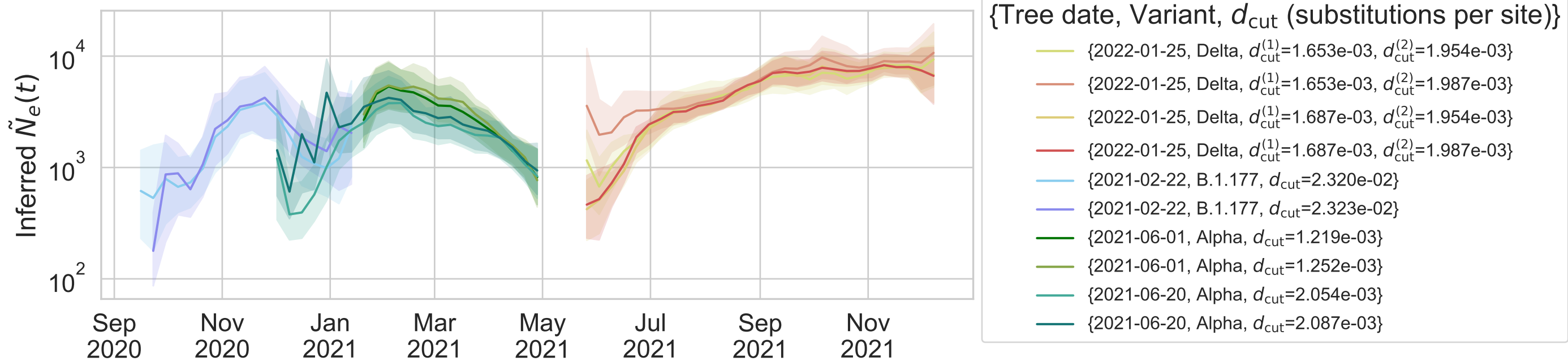

Supplement: S5 Fig — The tree date and depth used in the main text are {2021-02-22, B.1.177, dcut = 2.323 ⋅ 10−2}, {2021-06-20, Alpha, dcut = 2.054 ⋅ 10−3}, {2022-01-25, Delta, dcut(1)=1.687·10-3, dcut(2)=1.954·10-3}. The color of the lines for the parameters that were used in the main text are the same as those shown in Fig 2. (PDF) [file ppat.1012090.s008.pdf]

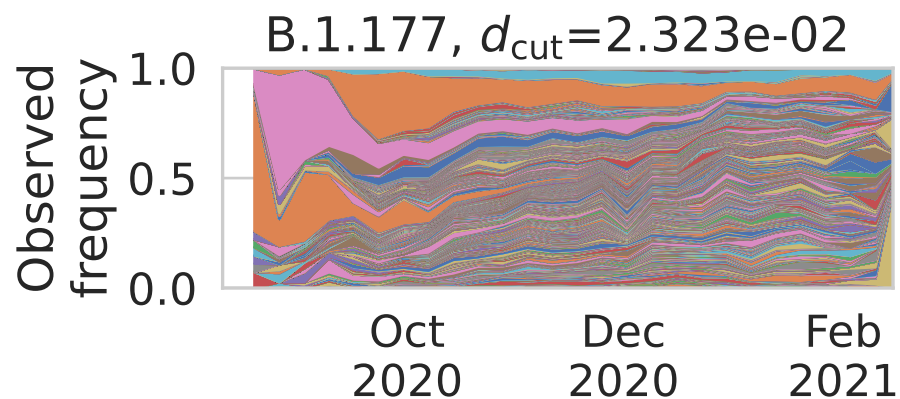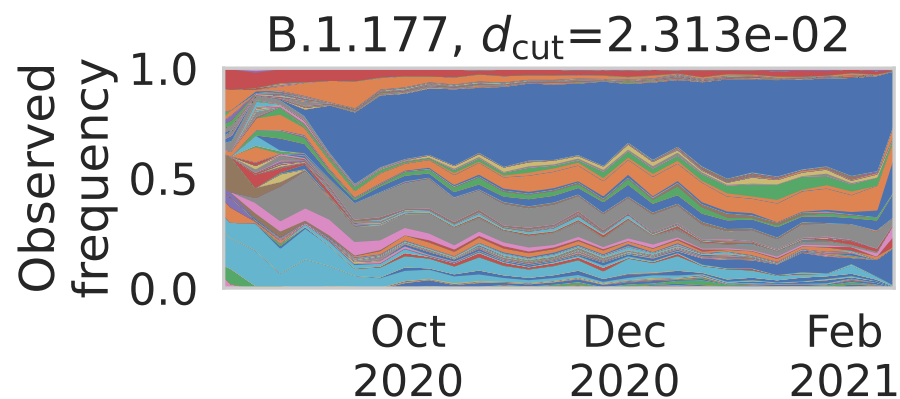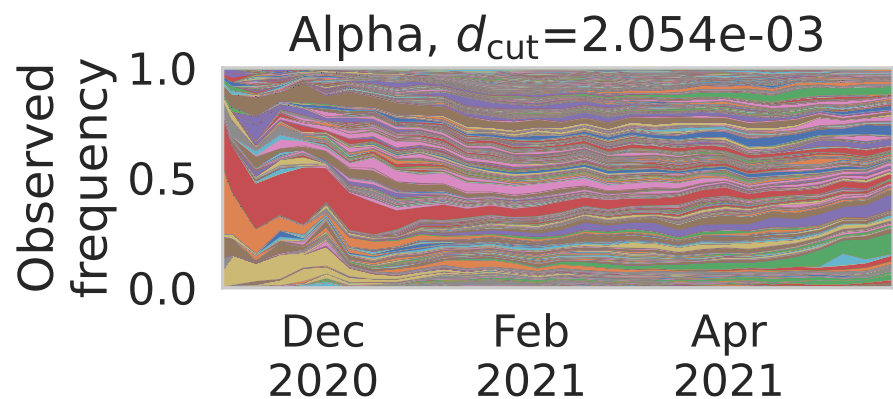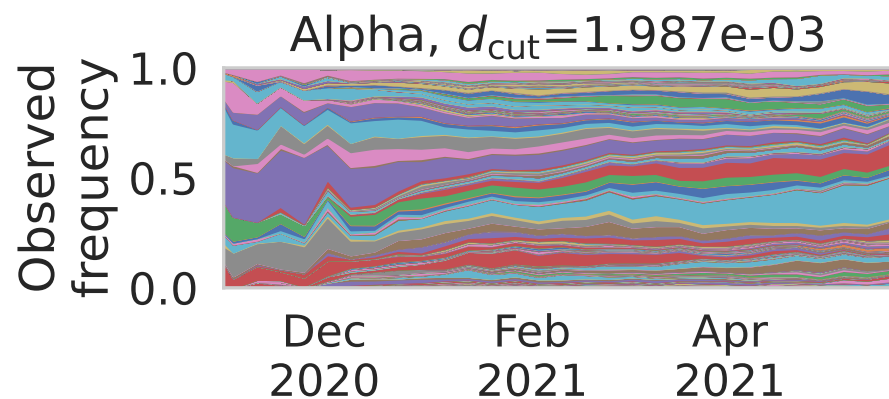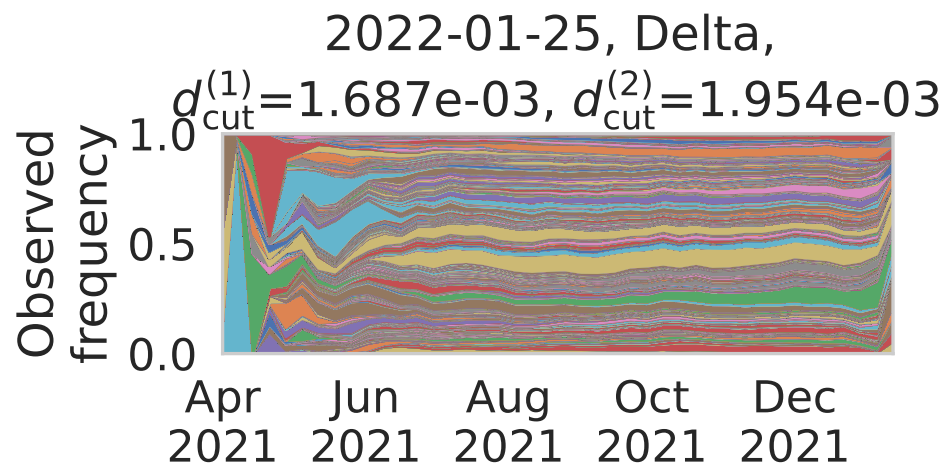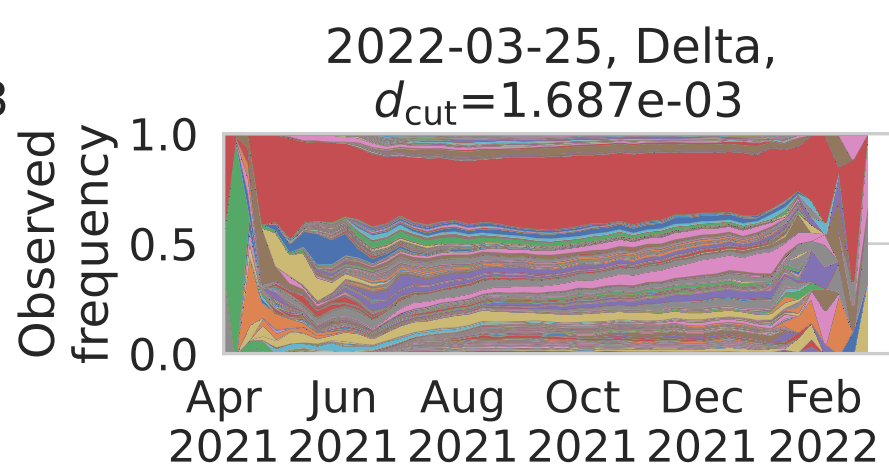

Supplement: S7 Fig — (PDF) [file ppat.1012090.s010.pdf]

pre-B.1.177

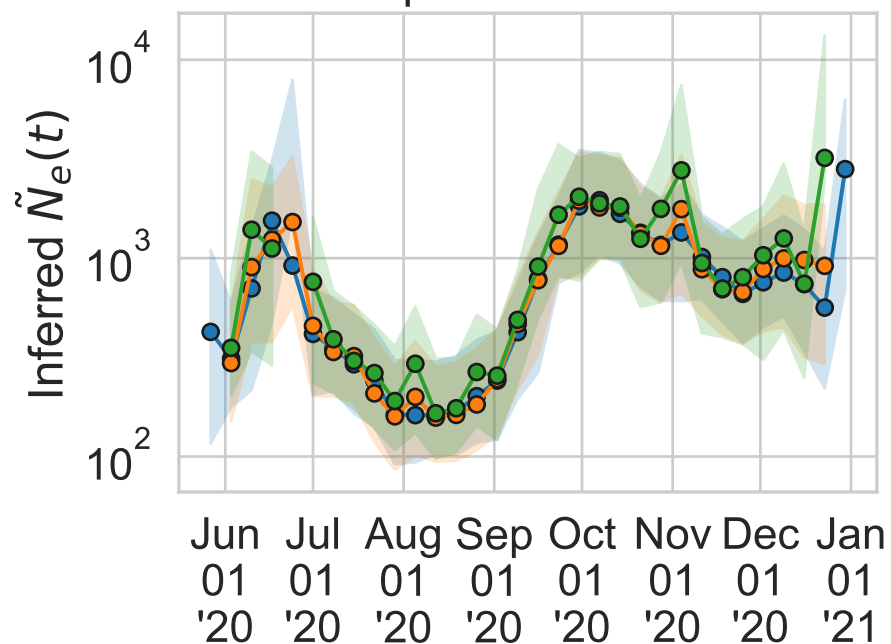

B.1.177

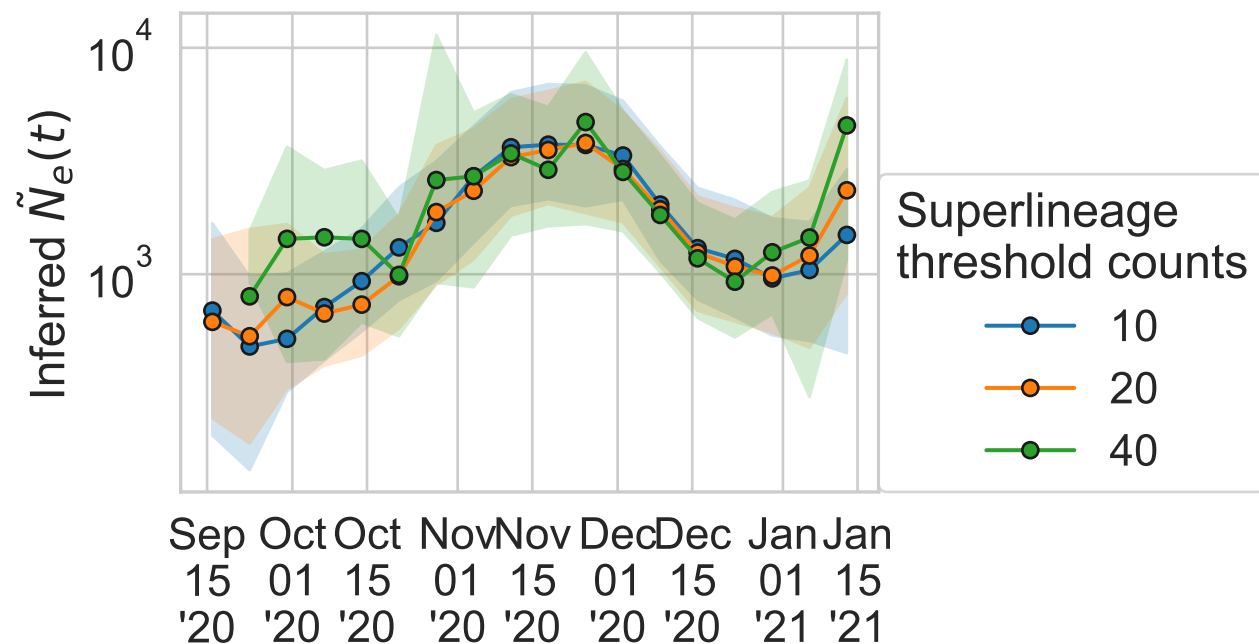

Alpha

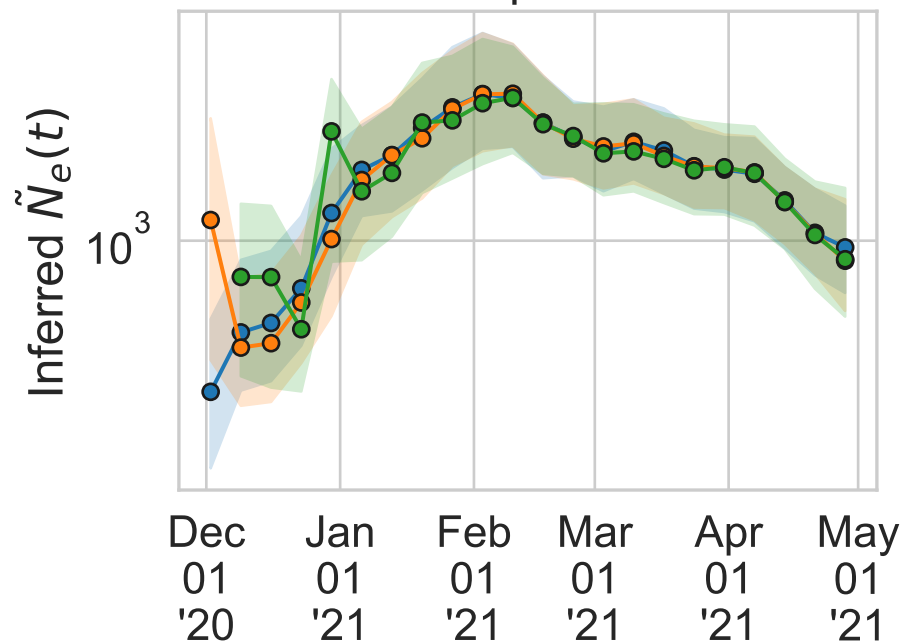

Delta

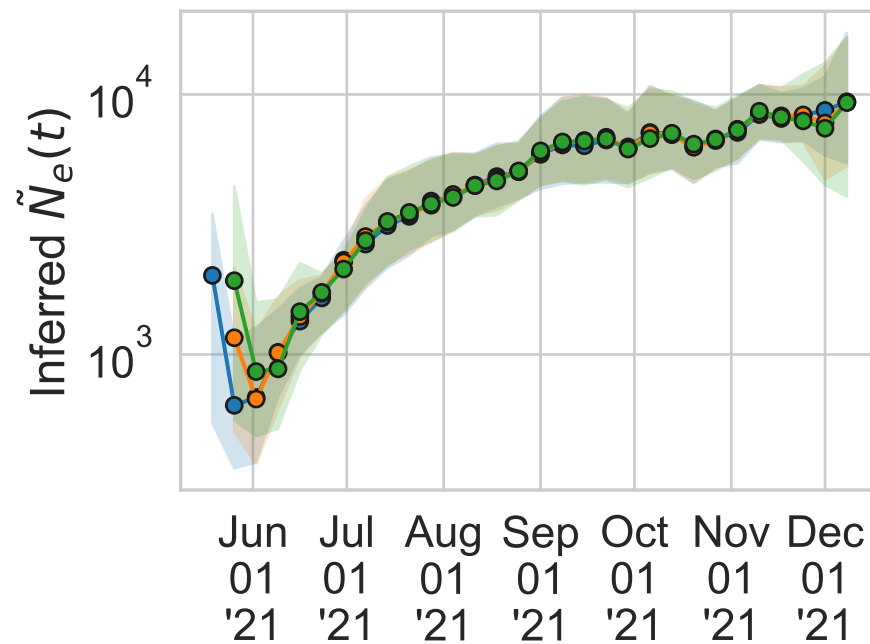

Supplement: S8 Fig — The coarse-grained lineage threshold counts used in the main text is 20. (PDF) [file ppat.1012090.s011.pdf]

pre-B.1.177

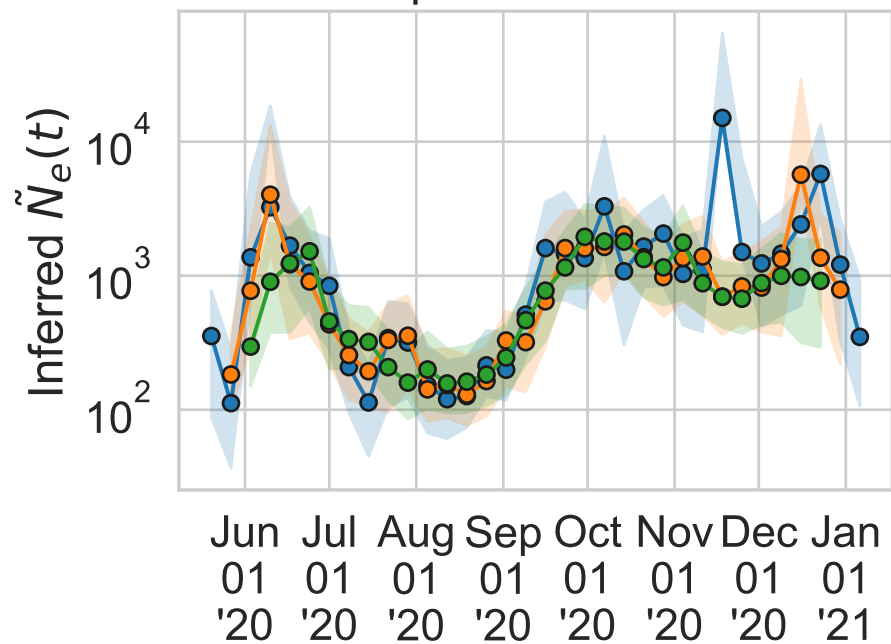

B.1.177

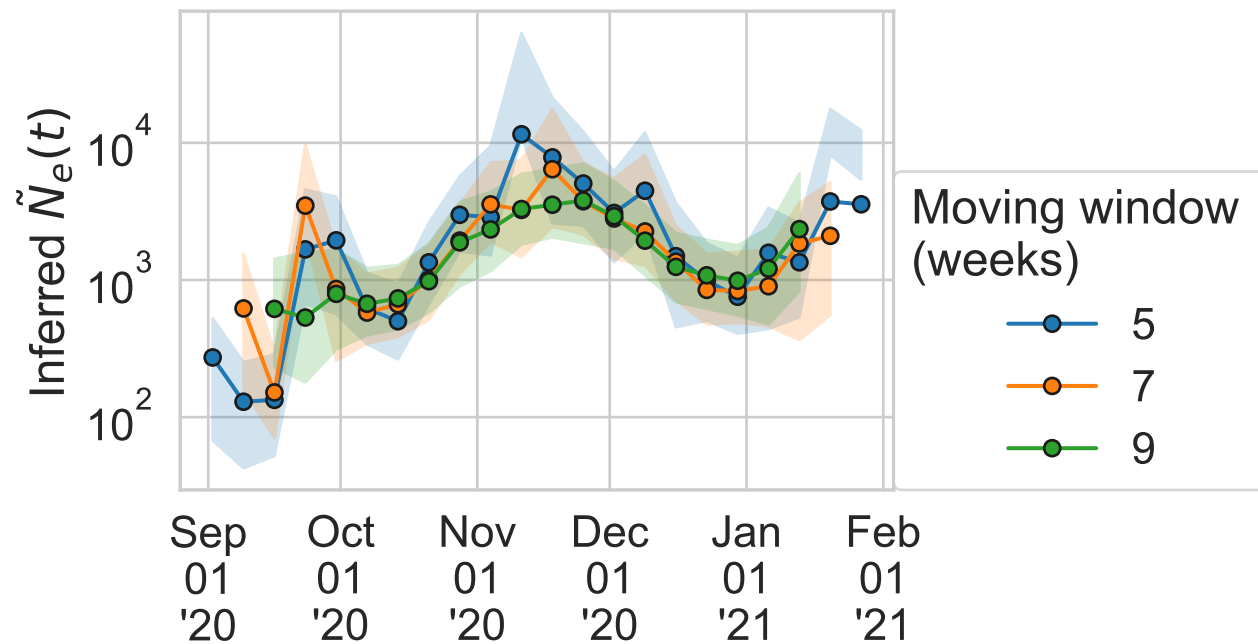

Alpha

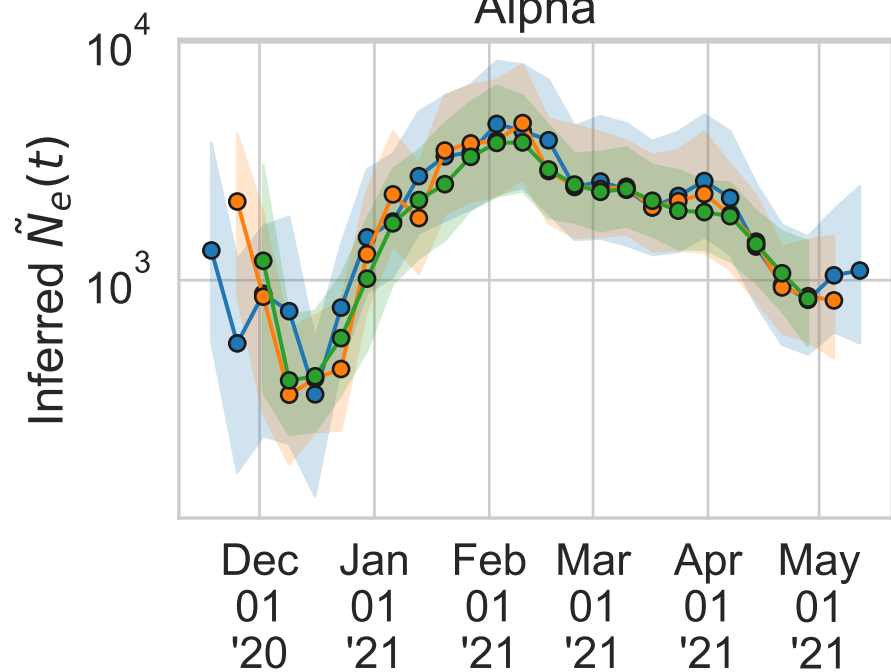

Delta

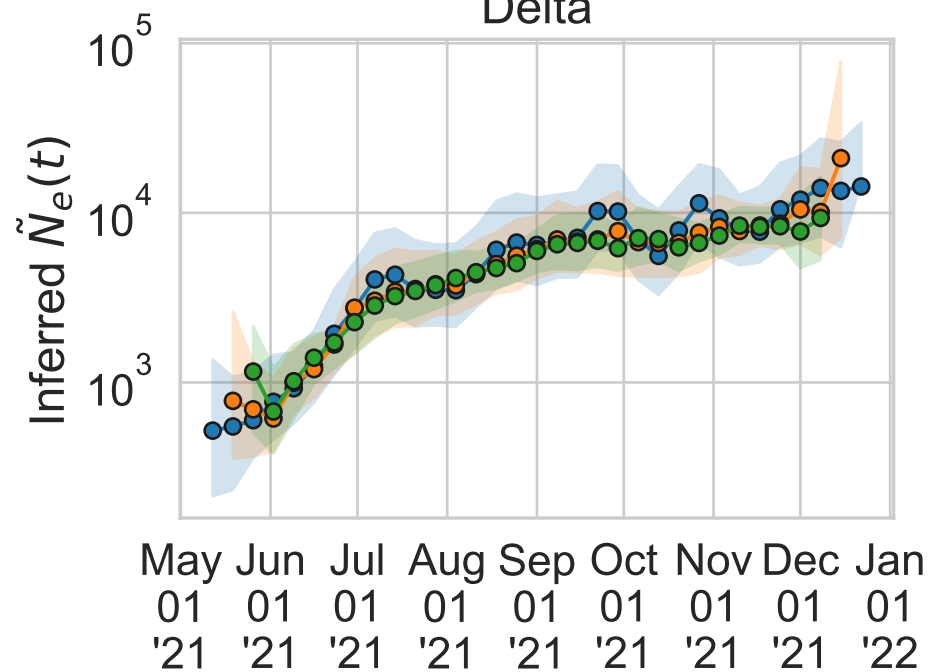

Supplement: S9 Fig — The size of the moving window used in the main text is 9 weeks. (PDF) [file ppat.1012090.s012.pdf]

pre-B.1.177

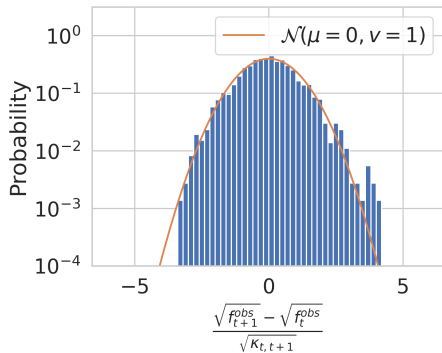

B.1.177

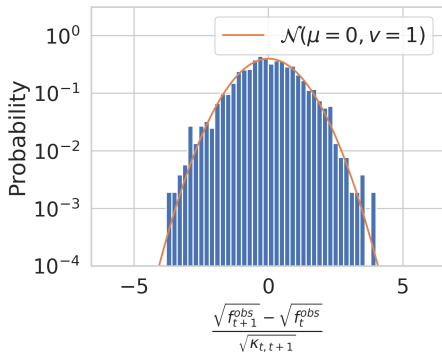

Alpha

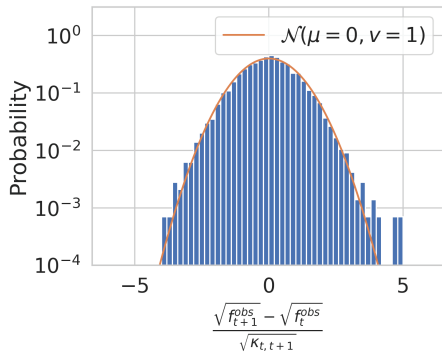

Delta

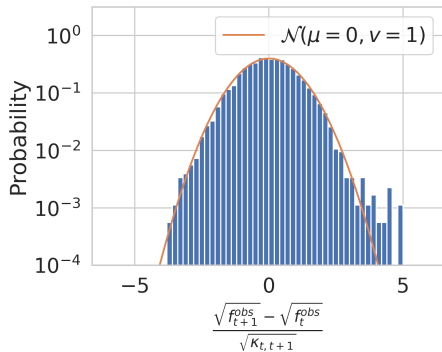

Supplement: S10 Fig — The orange line is a plot of a normal distribution with mean 0 and variance 1. (PDF) [file ppat.1012090.s013.pdf]

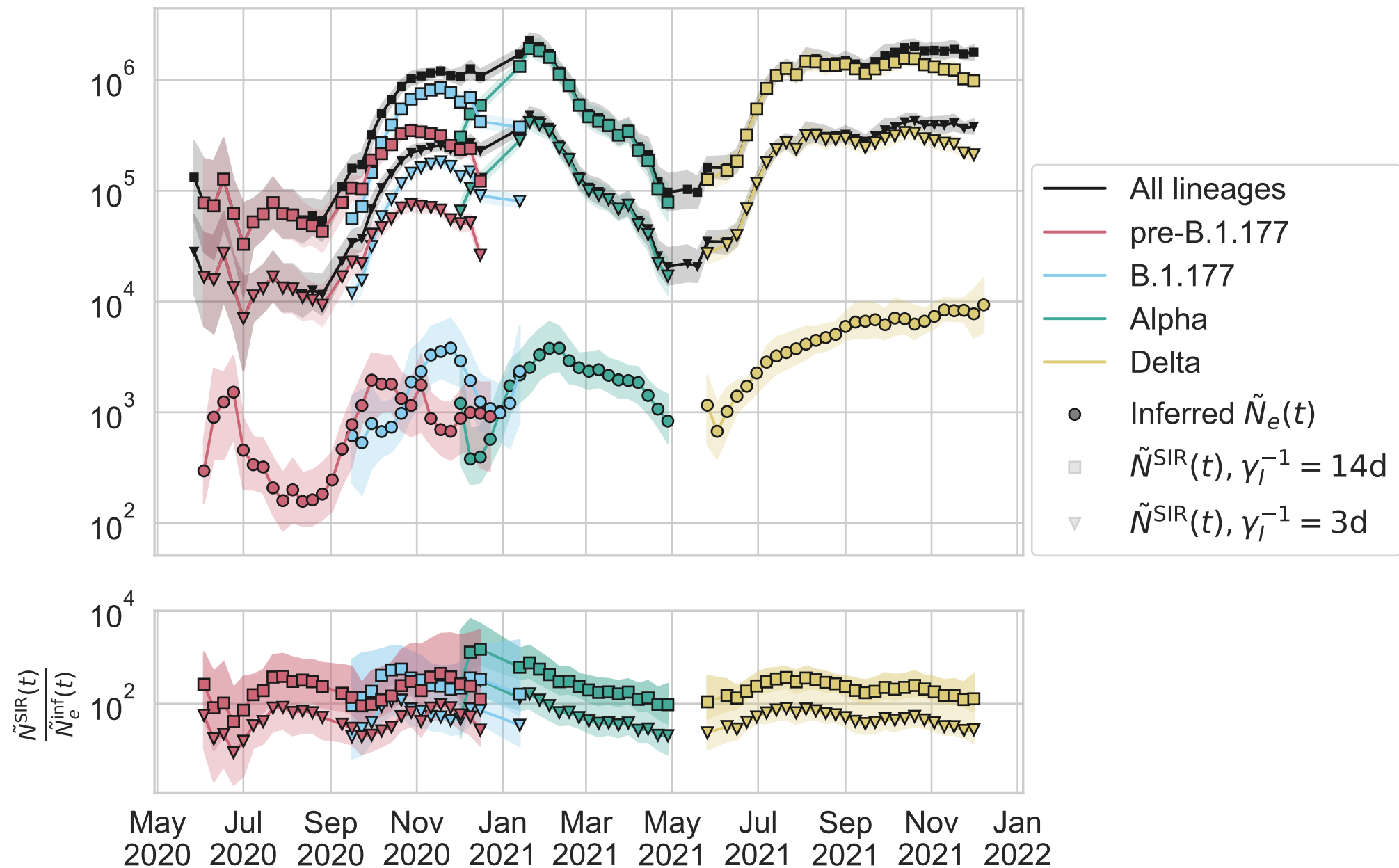

Supplement: S11 Fig — (PDF) [file ppat.1012090.s014.pdf]

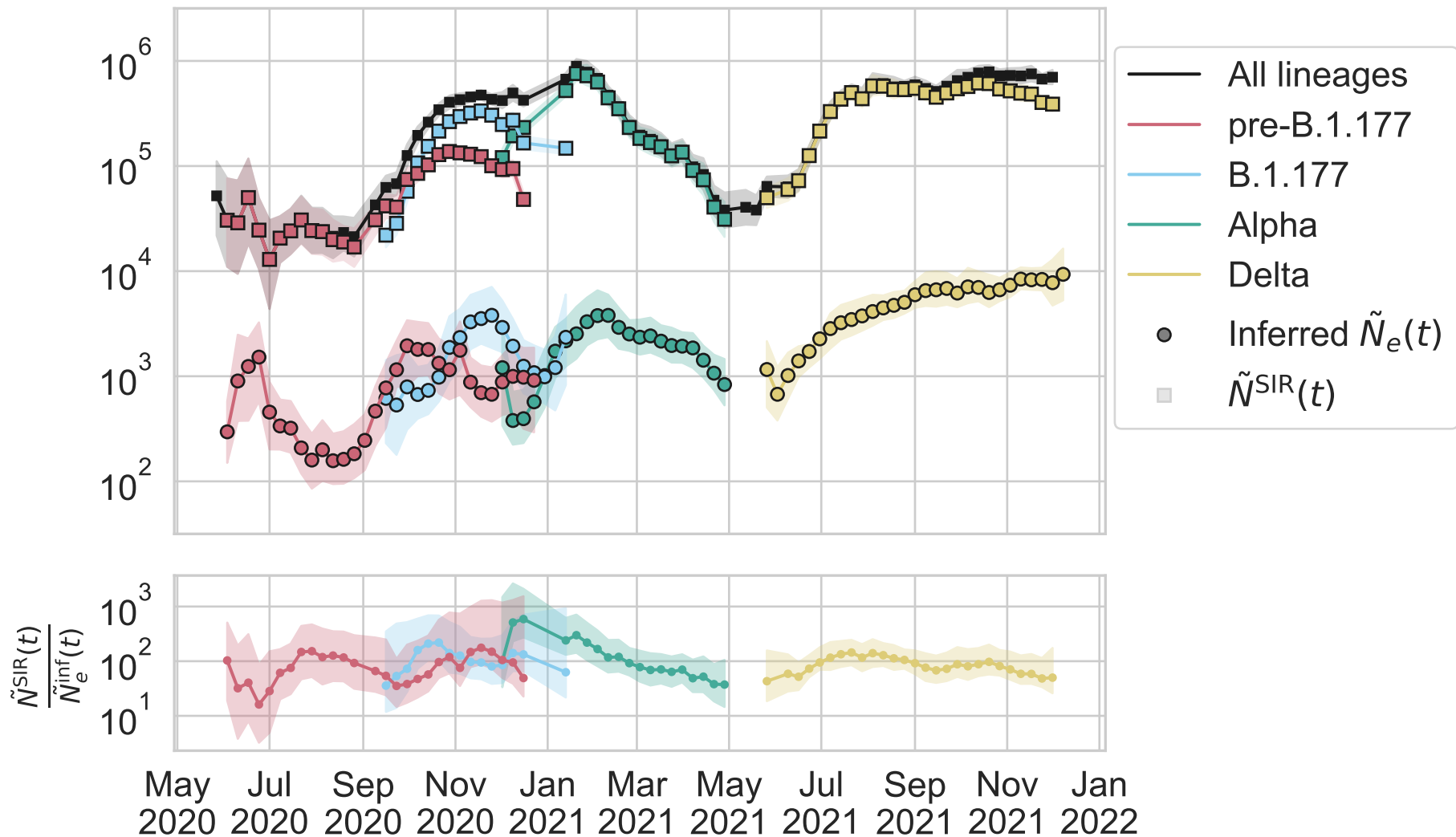

Supplement: S12 Fig — (PDF) [file ppat.1012090.s015.pdf]

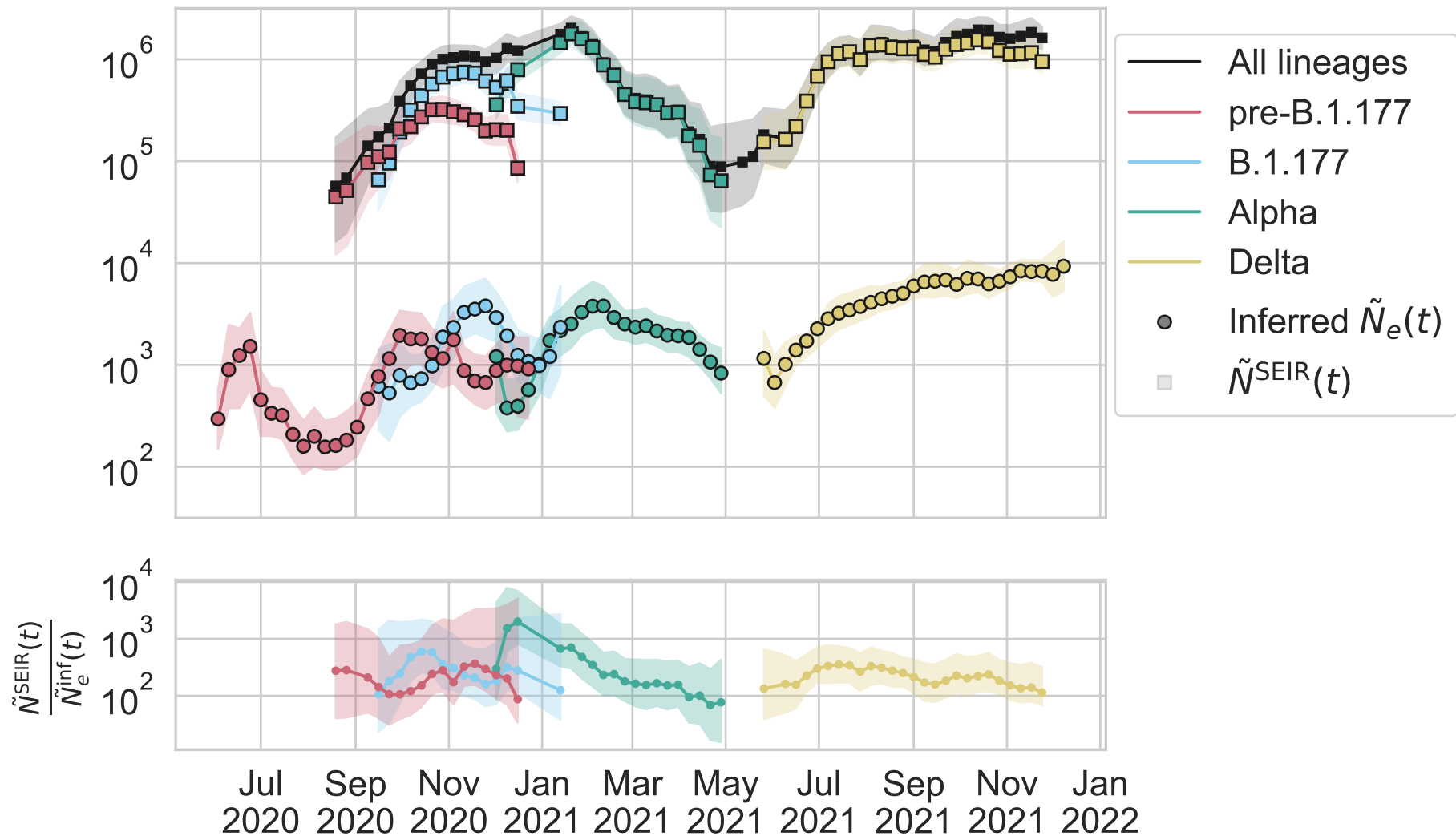

Supplement: S13 Fig — (PDF) [file ppat.1012090.s016.pdf]

pre-B.1.177

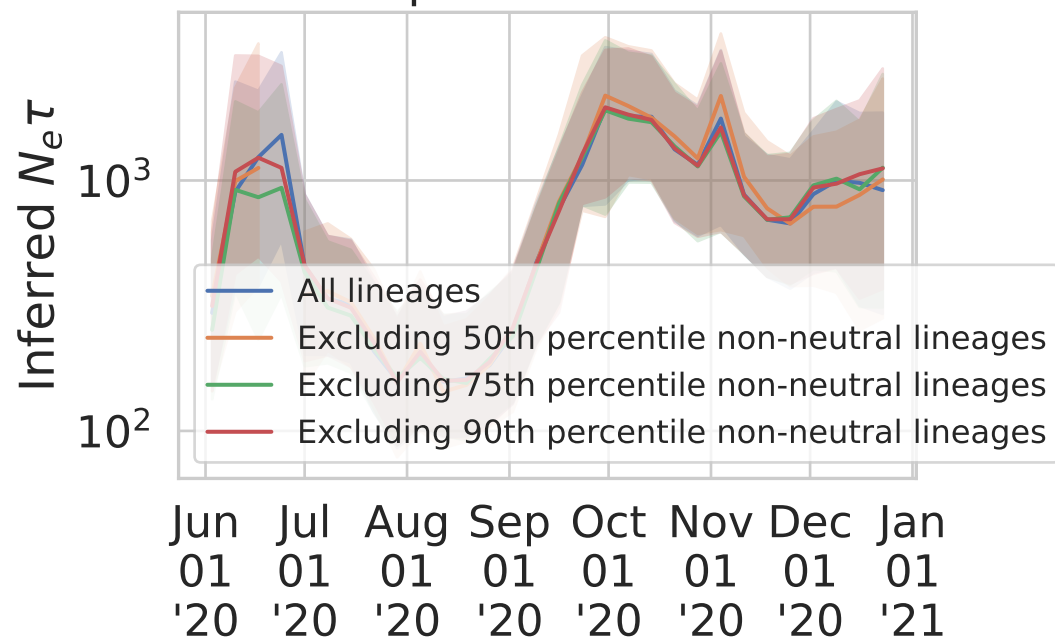

B.1.177

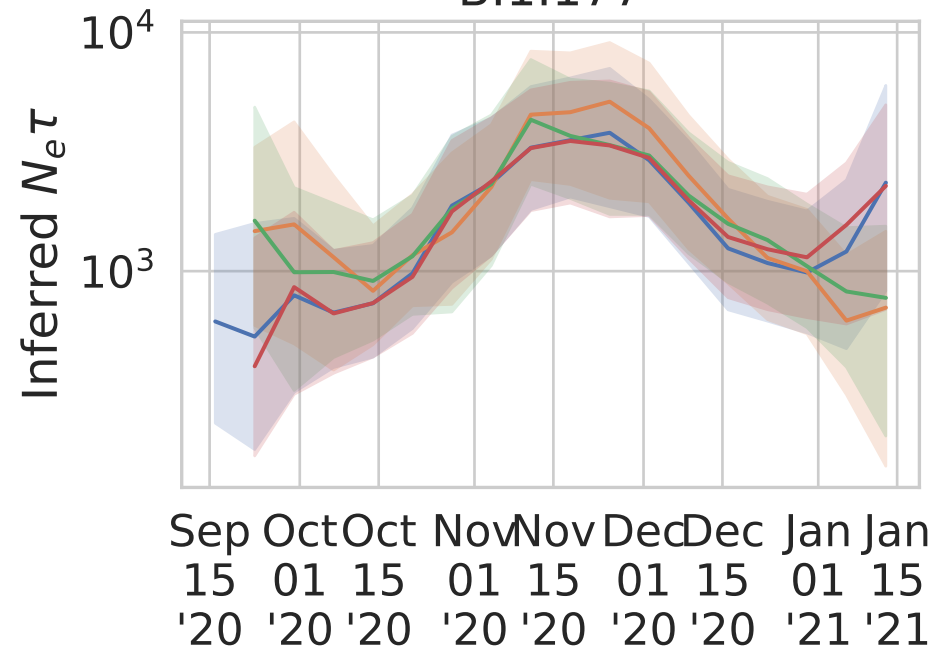

Alpha

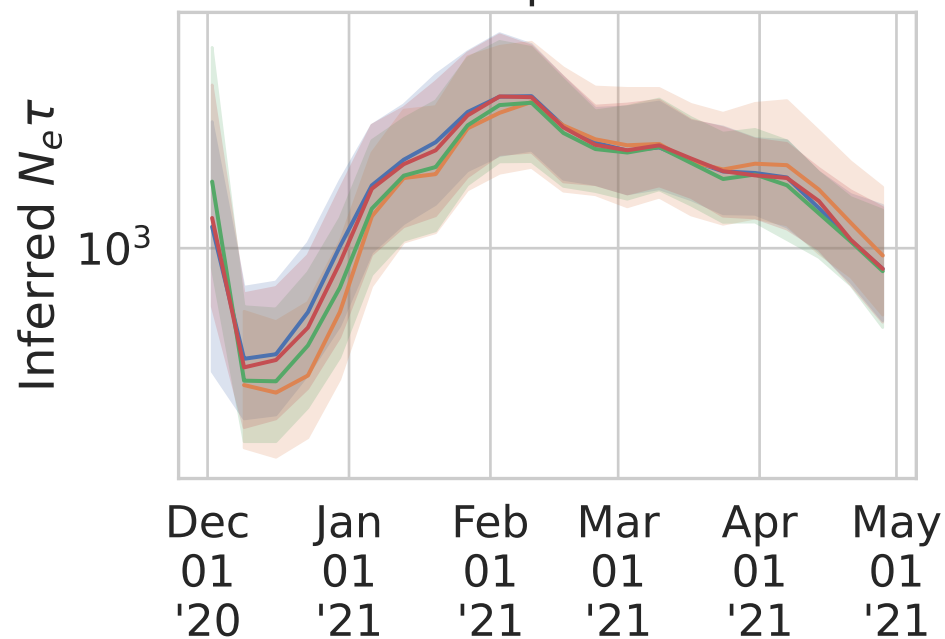

Delta

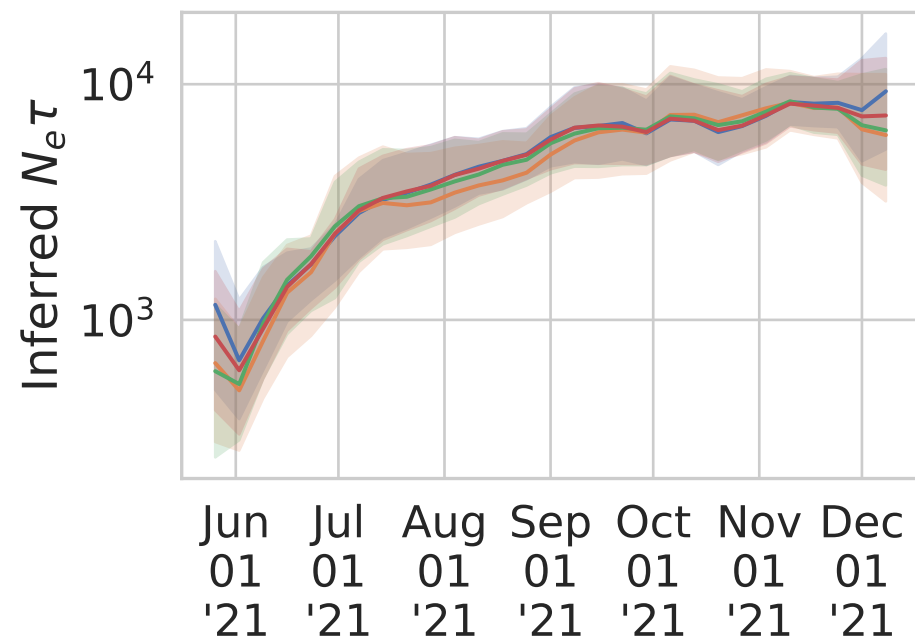

Supplement: S14 Fig — (PDF) [file ppat.1012090.s017.pdf]

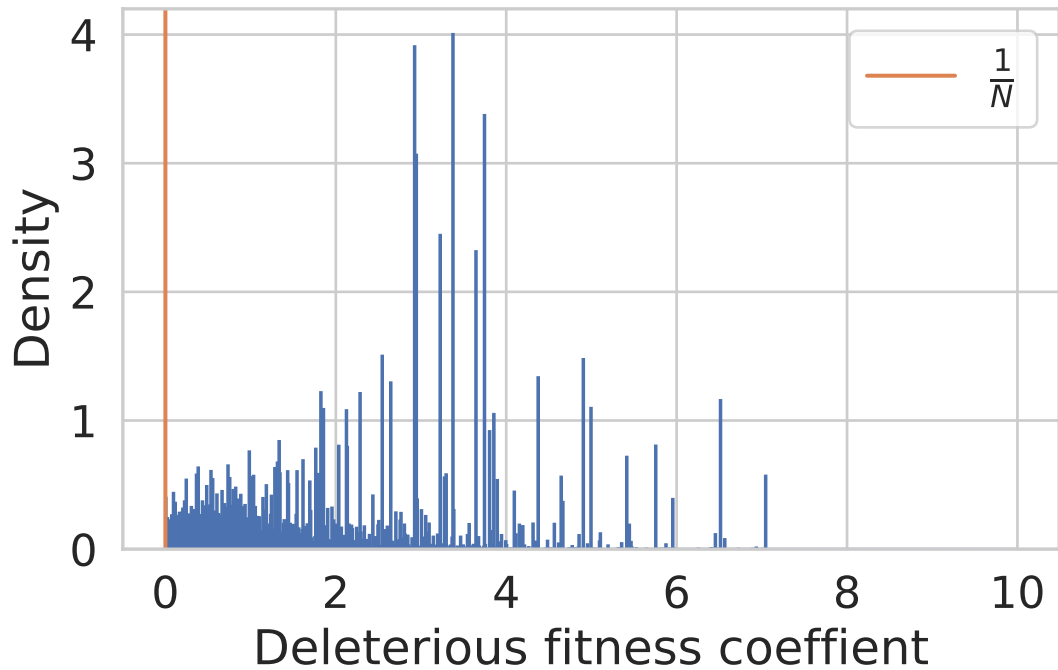

Supplement: S15 Fig — The orange vertical line indicates 1N, which is the threshold in fitness above which selection dominates over genetic drift. Here, N is set to 104, which is the order of magnitude of the census population size of SARS-CoV-2 in England. (PDF) [file ppat.1012090.s018.pdf]

1e4 DFE of deleterious mutations from Bloom and Neher

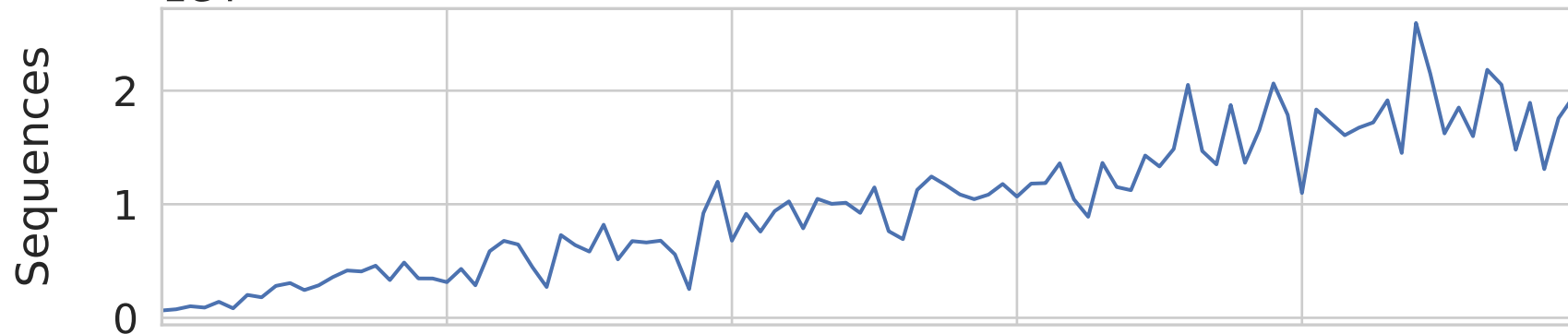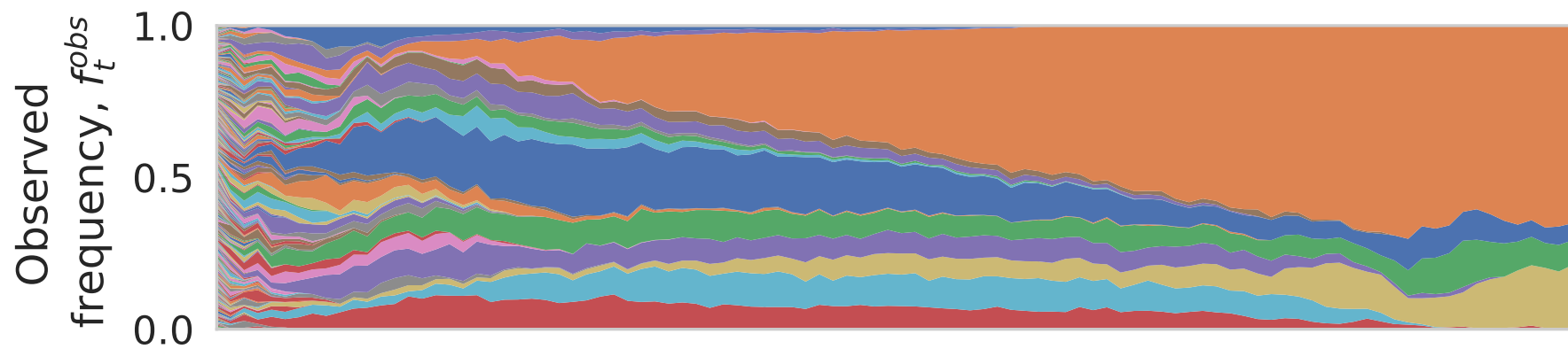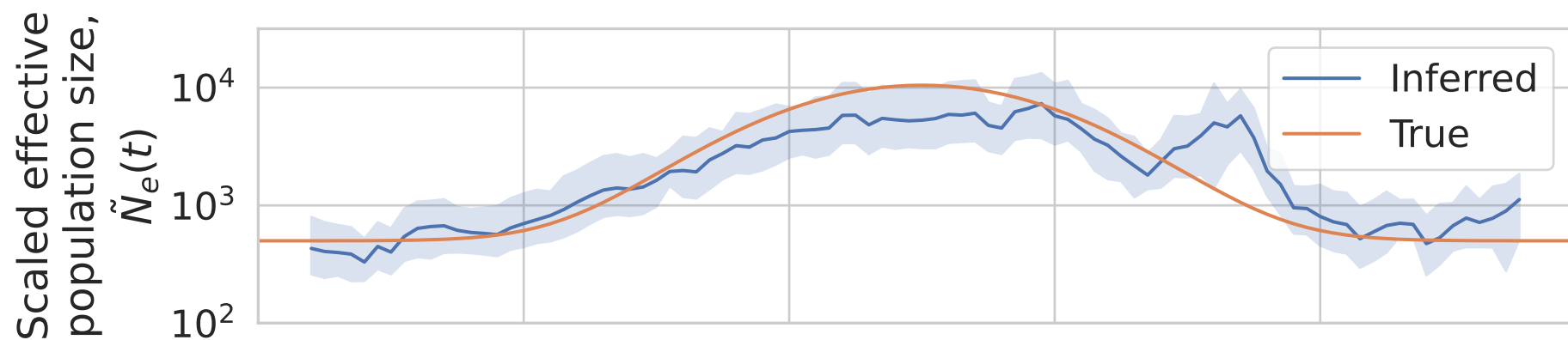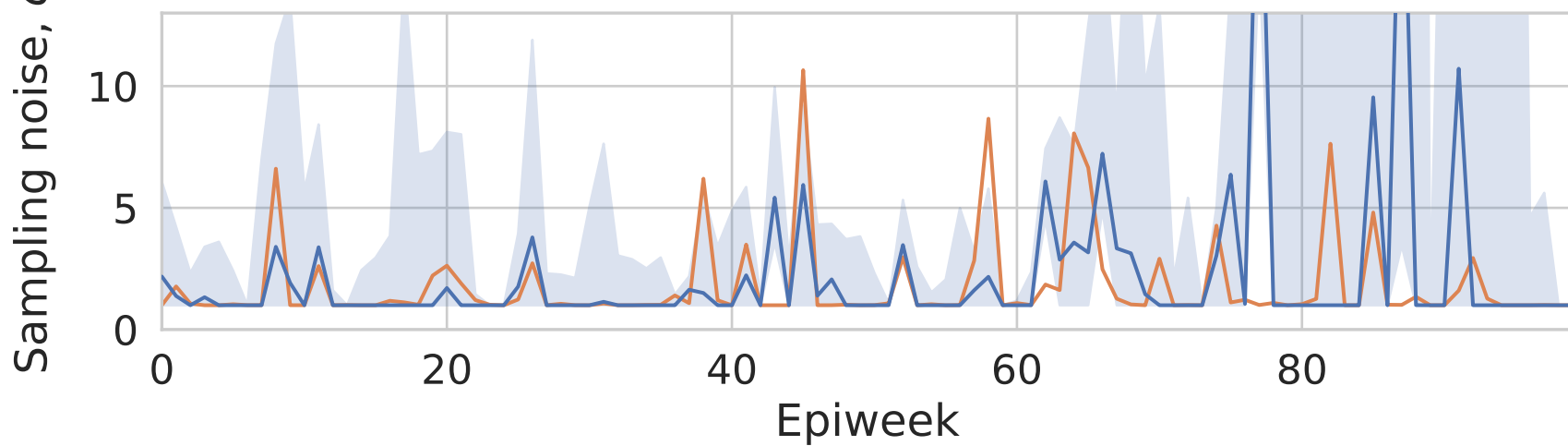

Epiweek

Supplement: S16 Fig — The inferred effective population size and measurement noise are shown. (PDF) [file ppat.1012090.s019.pdf]

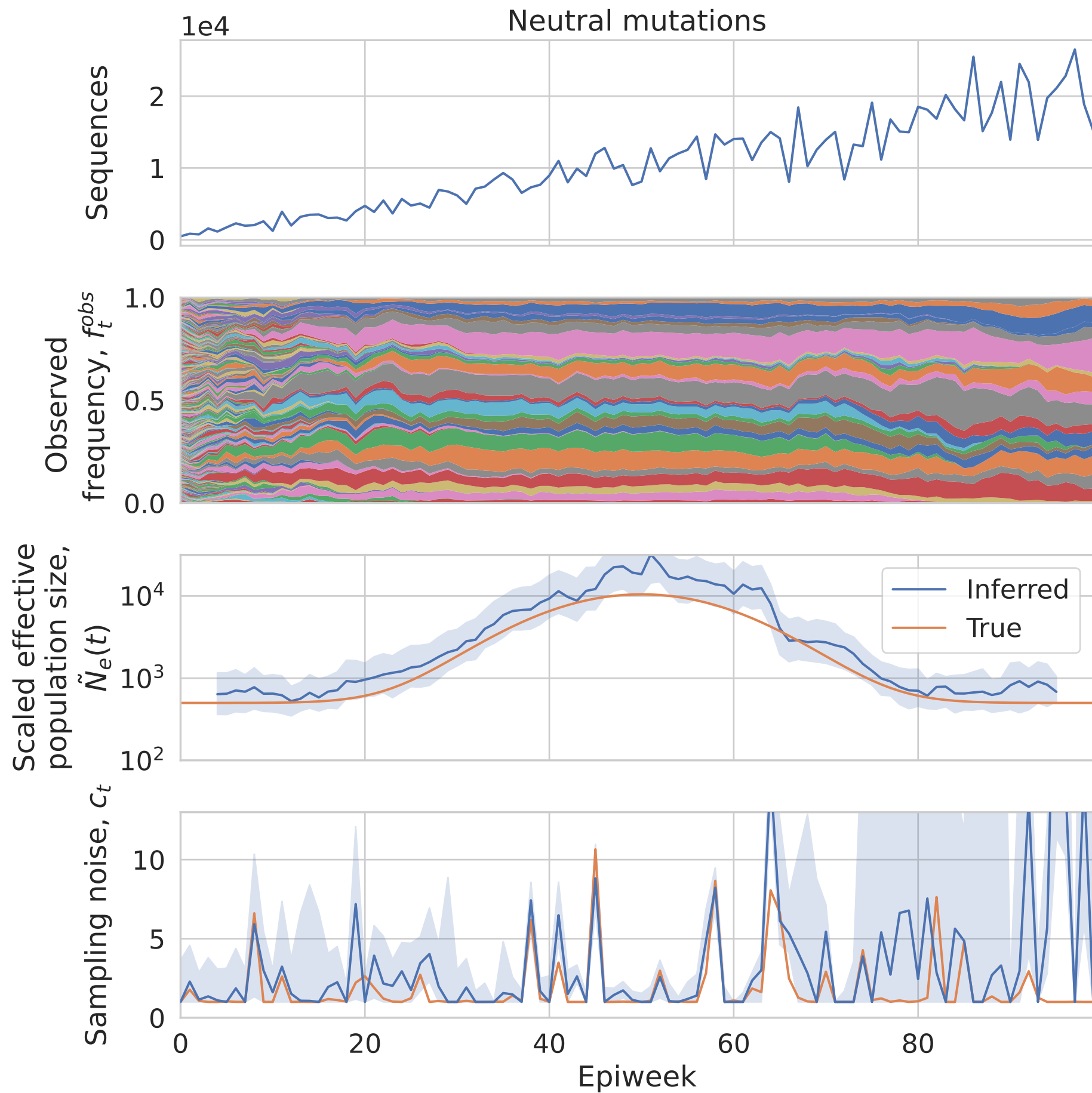

Supplement: S17 Fig — The inferred effective population size and measurement noise are shown. (PDF) [file ppat.1012090.s020.pdf]

gaussian

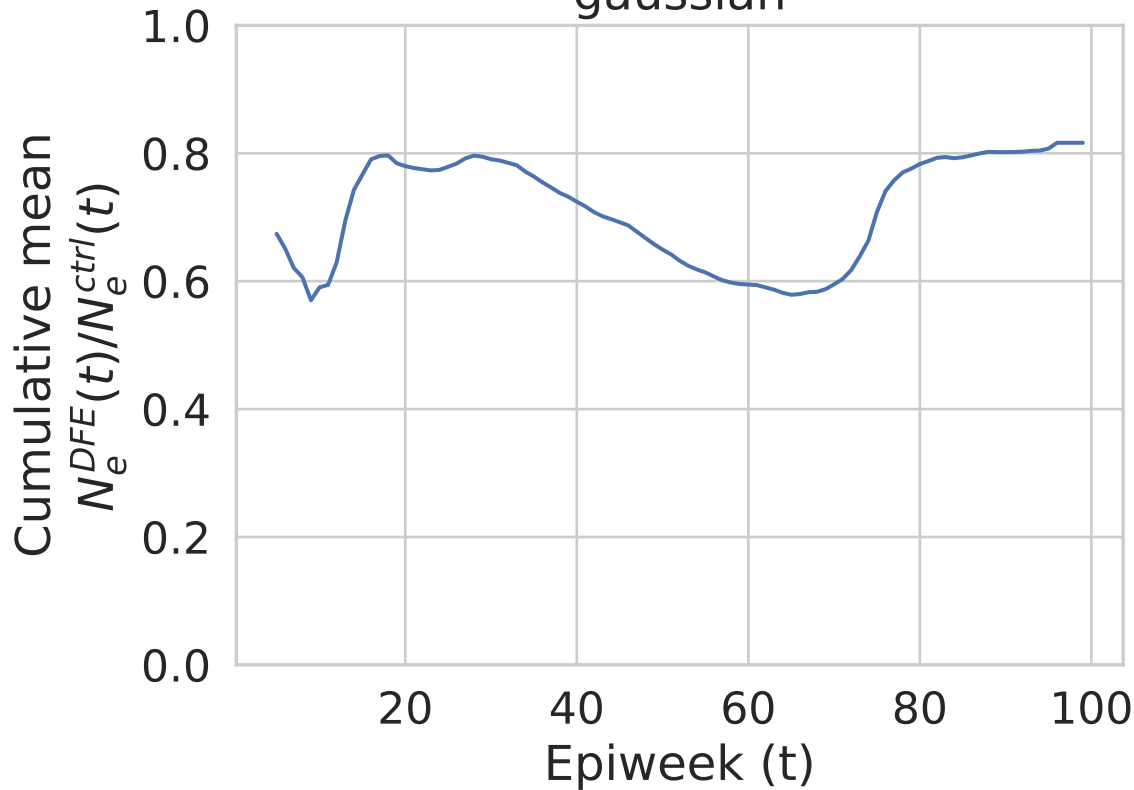

Supplement: S18 Fig — (PDF) [file ppat.1012090.s021.pdf]

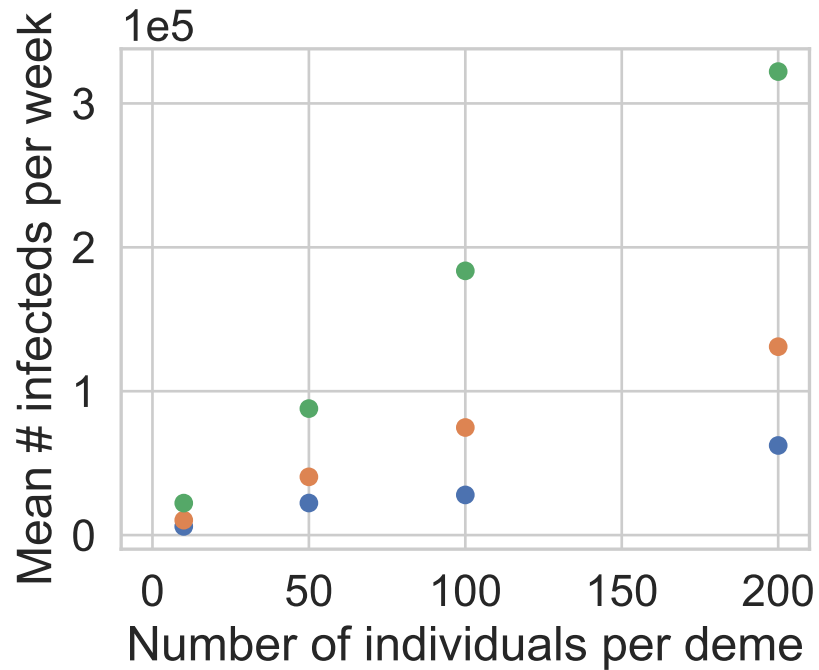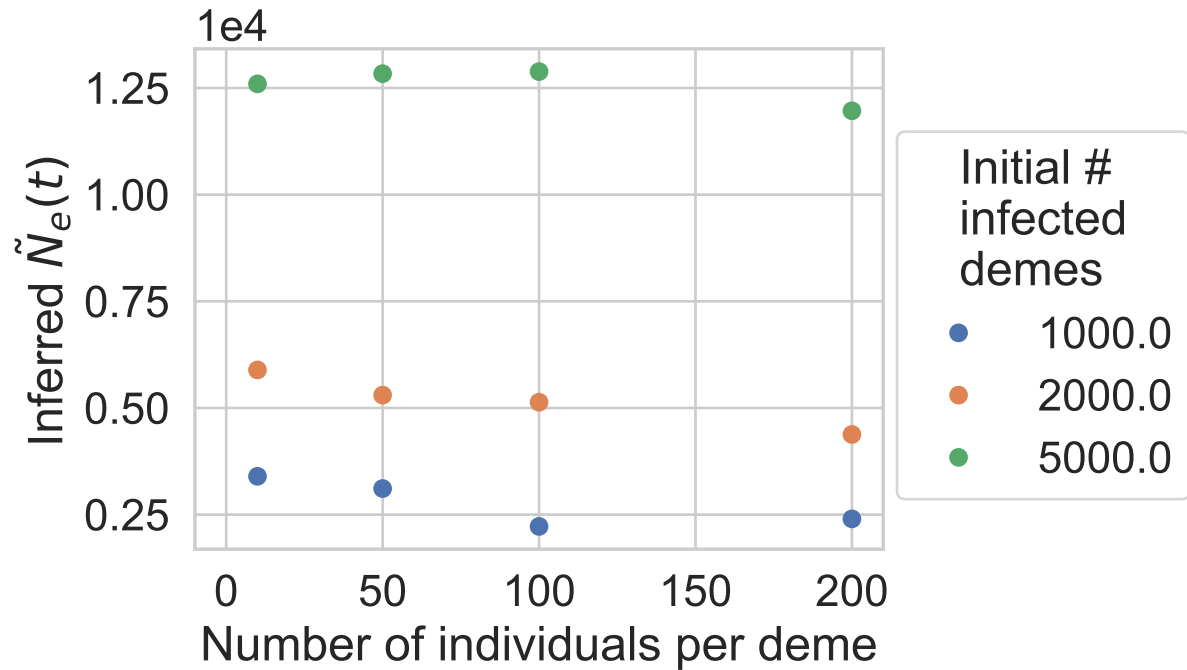

Supplement: S21 Fig — (a) The mean number of infected individuals per week from Weeks 42 to 50. (b) The inferred Ne˜(t) using lineage trajectories from Weeks 42 to 50. (PDF) [file ppat.1012090.s024.pdf]

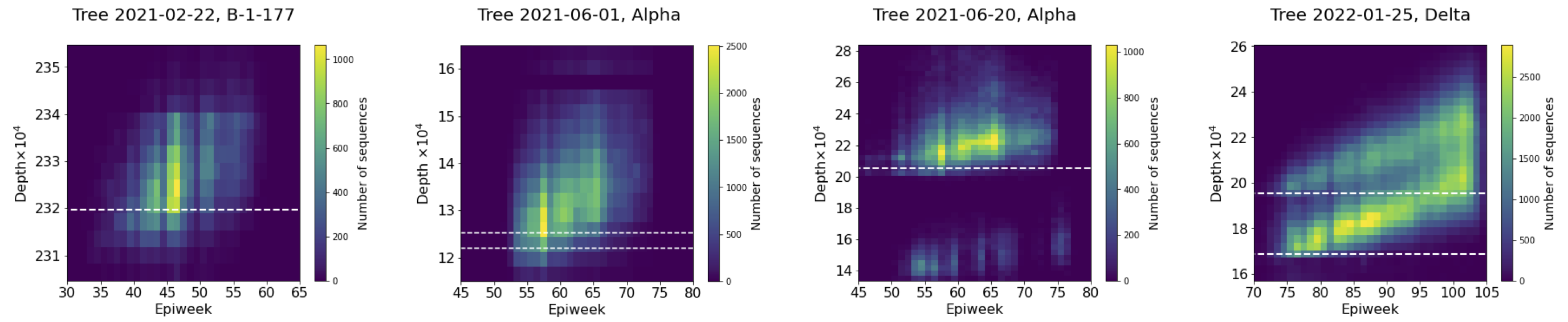

Supplement: S22 Fig — In a phylogenetic tree, the number of sequences (leaf nodes) of a focal variant that fall within specific epiweek and tree depth ranges is counted and summarized as a two-dimensional histogram. The tree depth is the substitution rate measured in units of substitutions per site, with respect to the most recent common ancestor. From left to right, the phylogenetic tree (specified by date created by COG-UK, using the sequences available at the time) and focal variant are {2021-02-22, B-1-177}, {2021-06-01, Alpha}, {2021-06-20, Alpha}, and {2022-01-25, Delta}. Weeks are counted from 2019-12-29. The dashed horizontal lines indicate the values of dcut (dcut(1) and dcut(2) for the Delta variant) used for the results presented in the main text, except for the 2021–06-01 Alpha tree, where they indicate the value of dcut tested in S5 Fig. (PNG) [file ppat.1012090.s025.png]

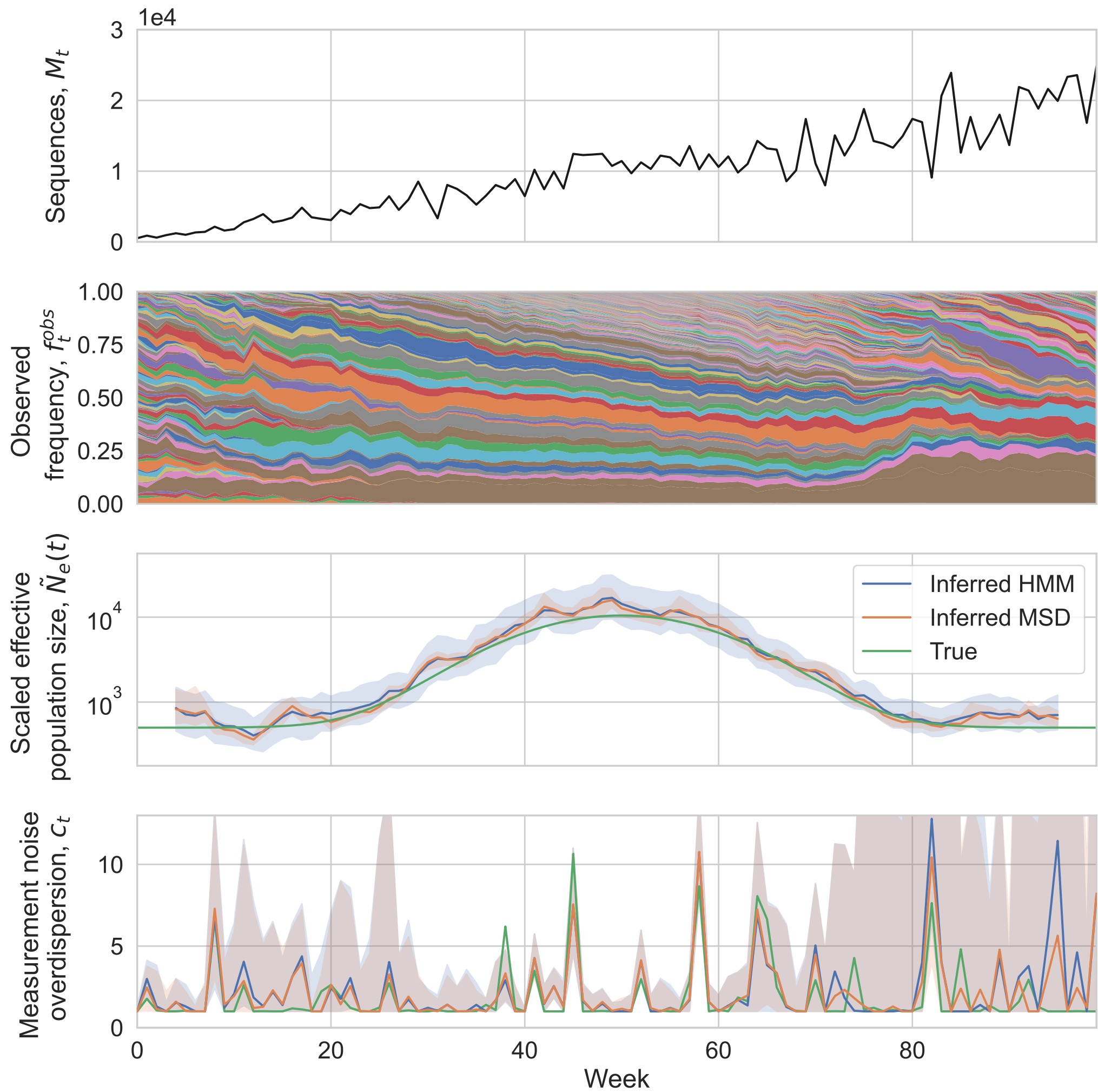

Supplement: S23 Fig — (a) Number of sequences sampled. (b) Simulated lineage frequency trajectories. (c) Inferred effective population size (Ne˜(t)) on simulated data using the method of moments (MSD, for mean squared displacement) and maximum likelhood (HMM, for Hidden Markov Model) estimation approaches compared to true values. The shaded region shows the 95% confidence interval of the inferred values. The confidence interval using the method of moments approach was calculated by taking the middle 95% of values when bootstrapping over the coarse-grained lineages. The confidence interval using the maximum likelihood estimation approach was determined using the posterior (see Methods) and takes into account joint errors in ct and Ne˜(t). (d) Inferred measurement noise (ct) on simulated data using the method of moments and maximum likelihood estimation approaches compared to true values. The shaded region shows the 95% confidence interval calculated using bootstrapping (see Methods). (PDF) [file ppat.1012090.s026.pdf]

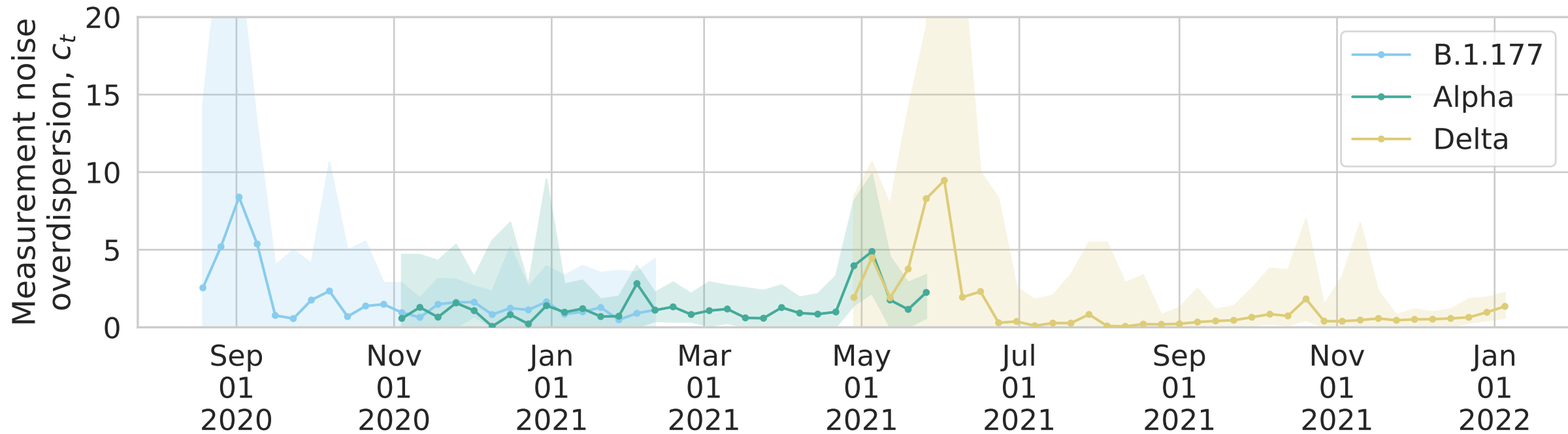

Supplement: S25 Fig — (PDF) [file ppat.1012090.s028.pdf]

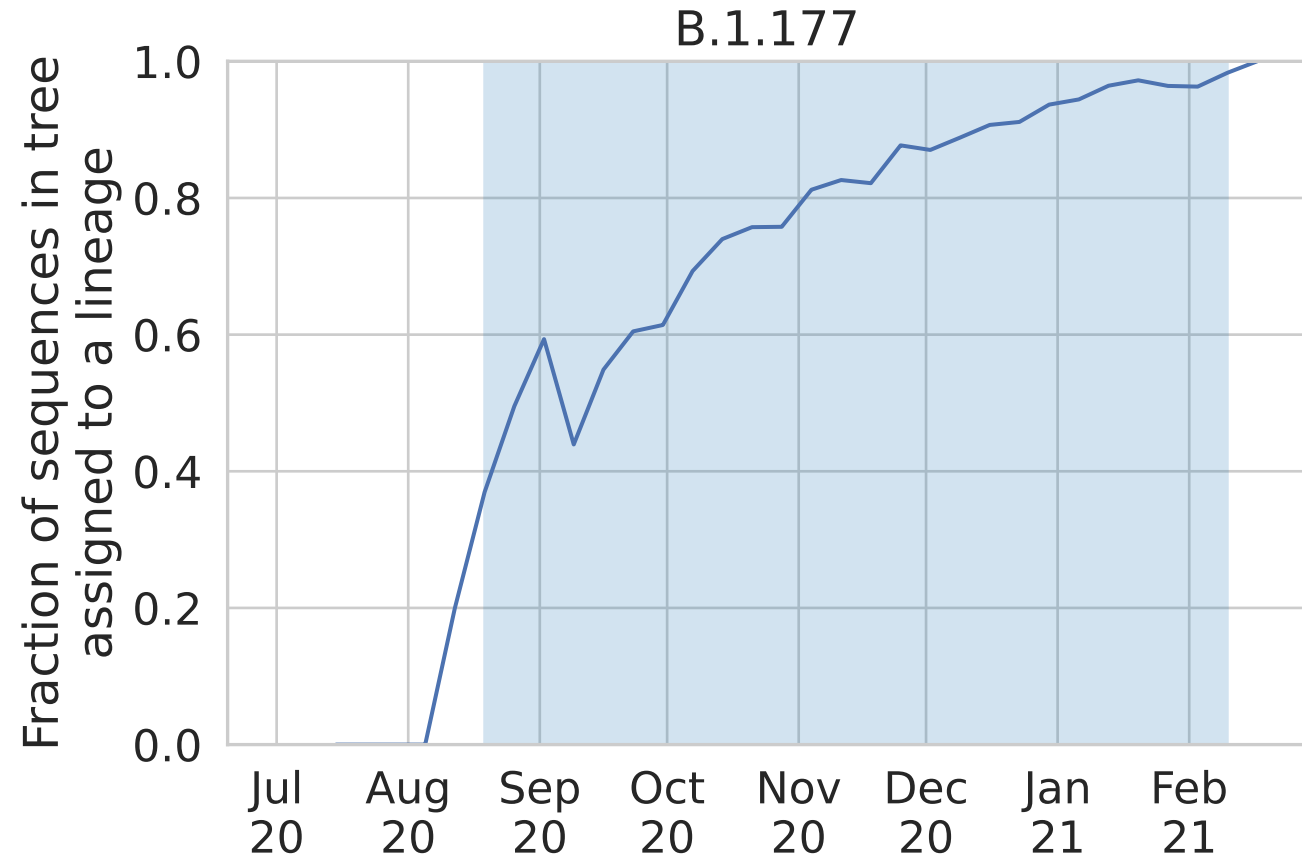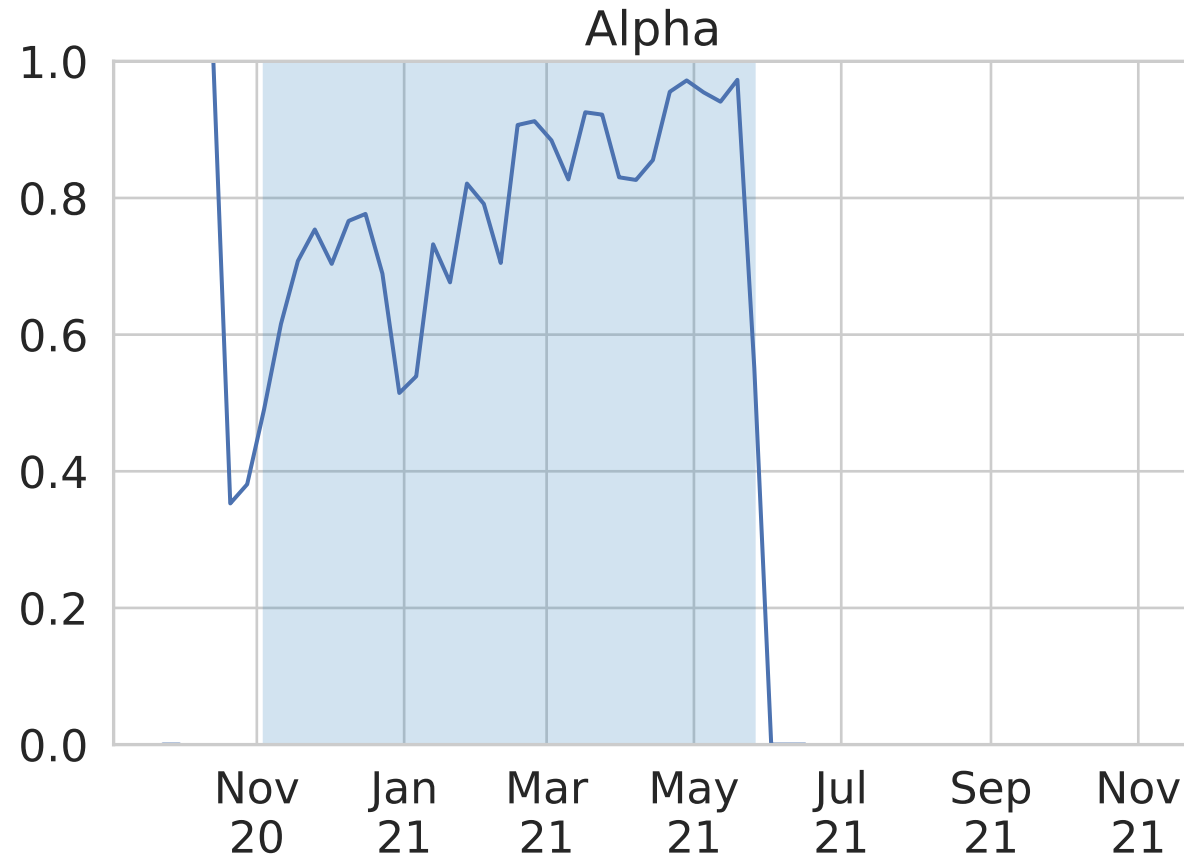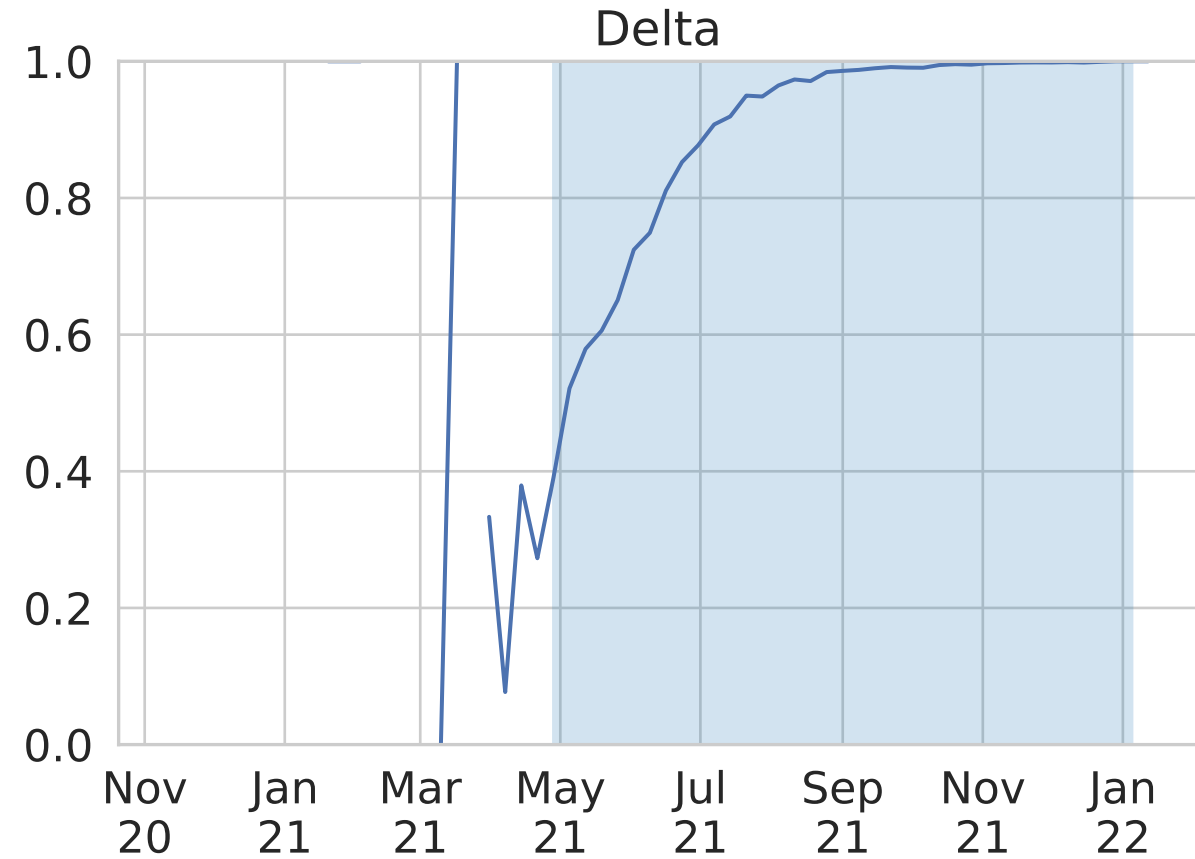

Supplement: S26 Fig — The blue shading indicates the period of time in the data that was used for the inference analysis. (PDF) [file ppat.1012090.s029.pdf]

{2022-01-25, Delta,  $d_{\text{cut}}^{(1)}=1.653\text{e-}03$ ,  $d_{\text{cut}}^{(2)}=1.954\text{e-}03$ }

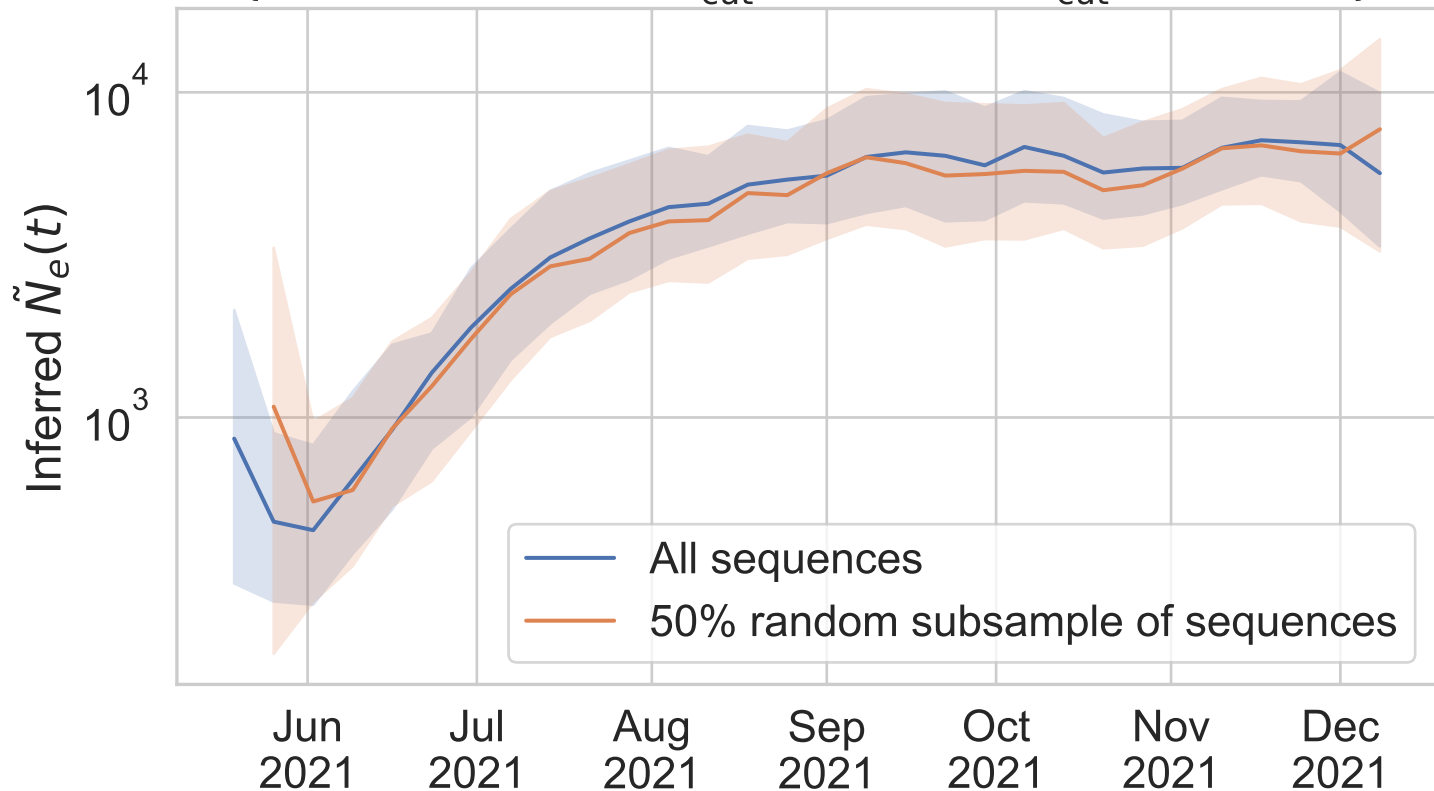

Supplement: S27 Fig — (PDF) [file ppat.1012090.s030.pdf]

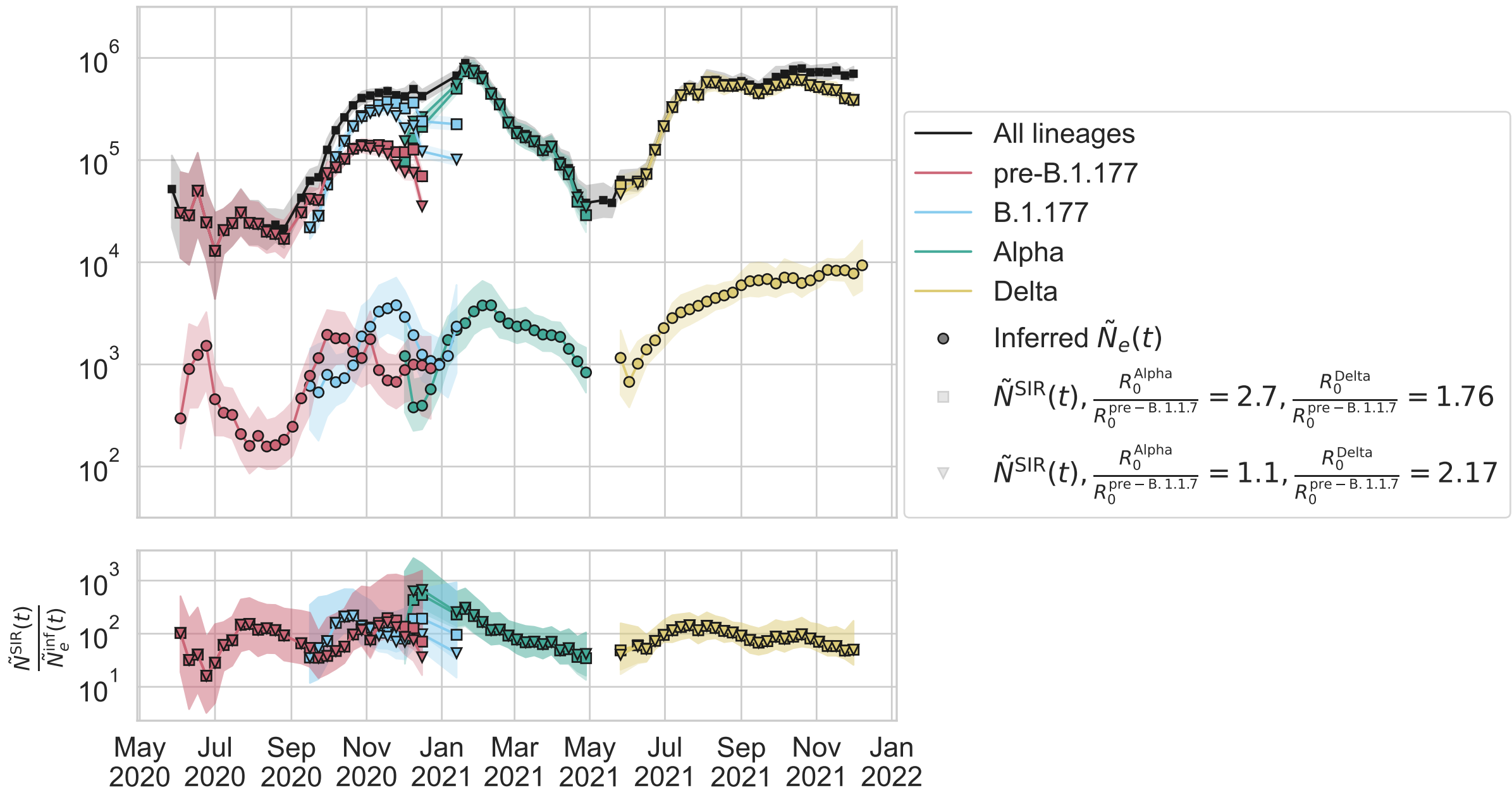

Supplement: S28 Fig — (PDF) [file ppat.1012090.s031.pdf]

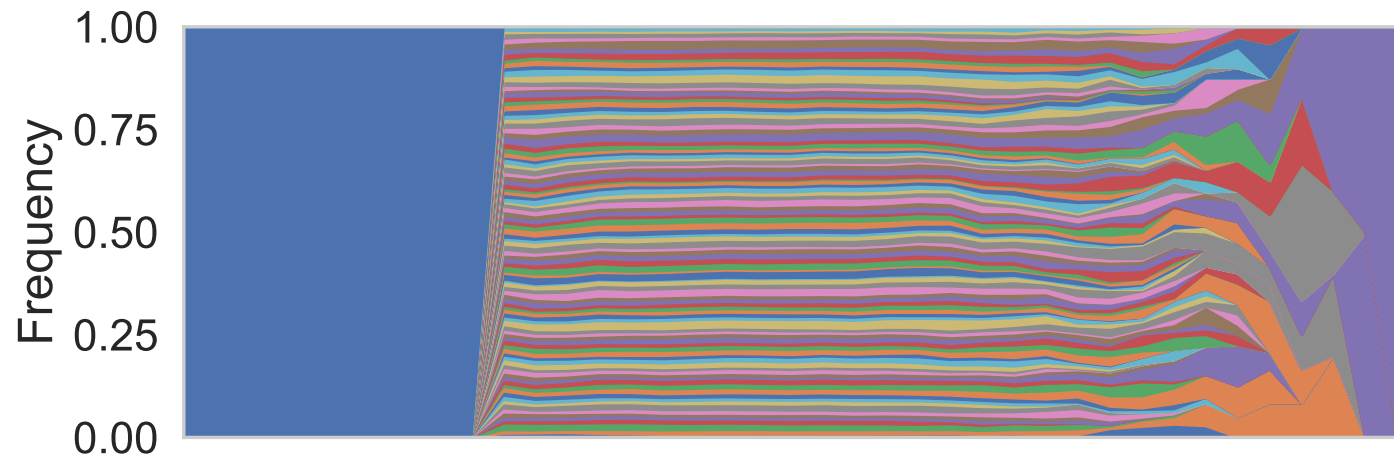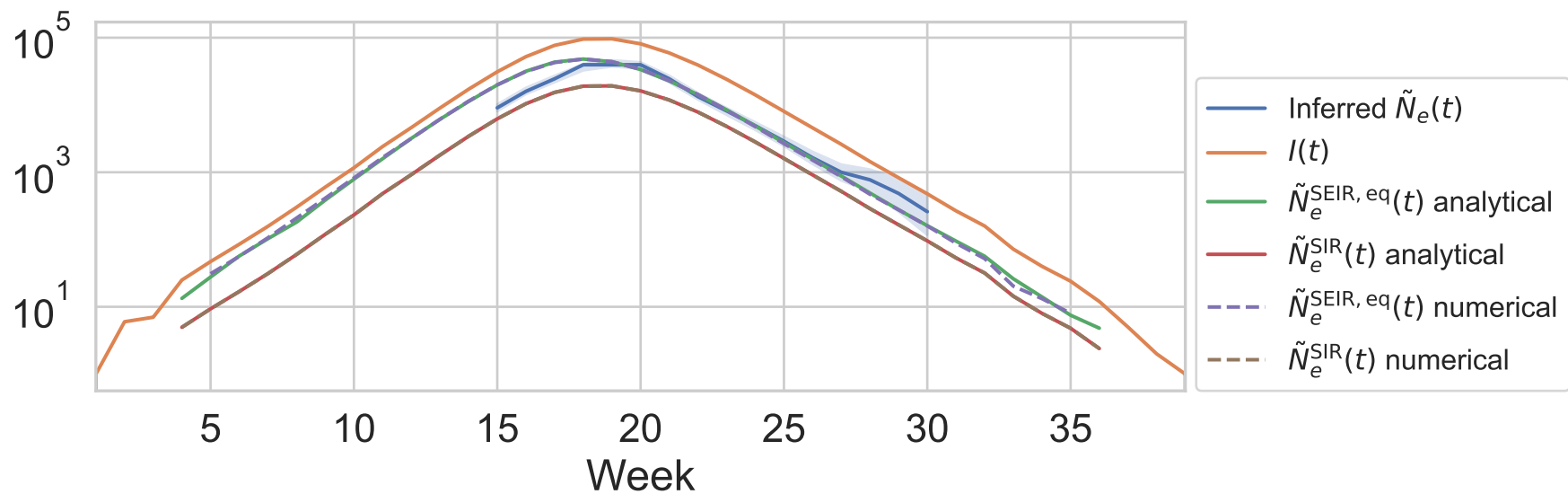

Supplement: S29 Fig — (Top) Muller plot of simulated infectious individuals’ lineage trajectories (simulations described in Methods). Infectious individuals are randomly assigned a lineage in week 11, and individuals that they transmit to are infected with the same lineage. The blue lineage before week 11 indicates the infectious individuals that existed before lineages were assigned. (Bottom) Comparison of the inferred Ne˜(t) using the lineage trajectories shown in the top panel to the number of infectious individuals I(t), Eq 49 (SEIR model Ne˜(t) at equilibrium), and Eq 1 (SIR model Ne˜(t)) calculated analytically or numerically as described in the Methods. The numerical solutions give the same results as the analytical solutions. (PDF) [file ppat.1012090.s032.pdf]

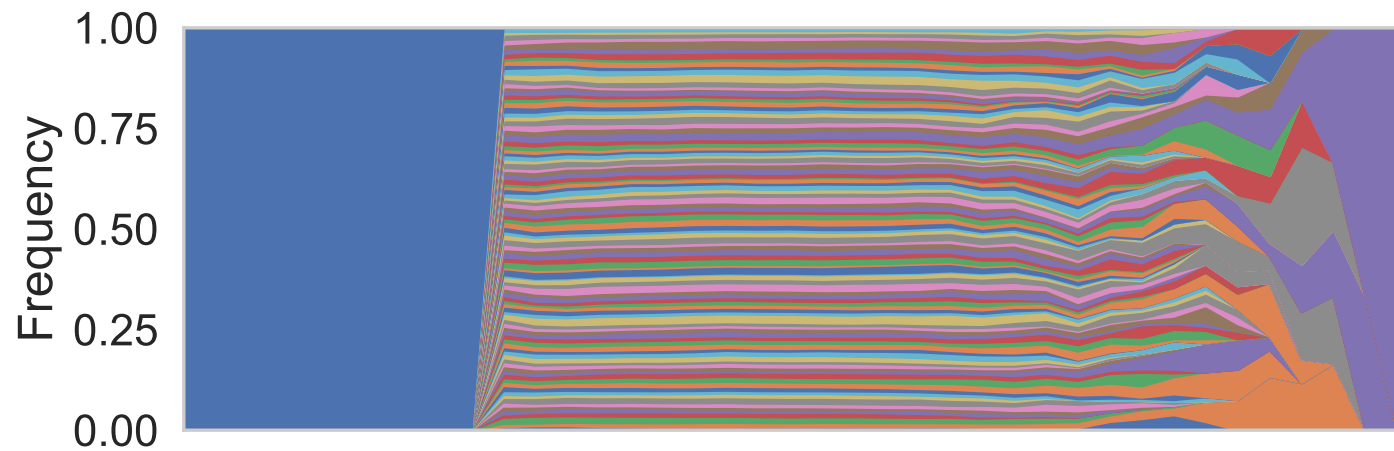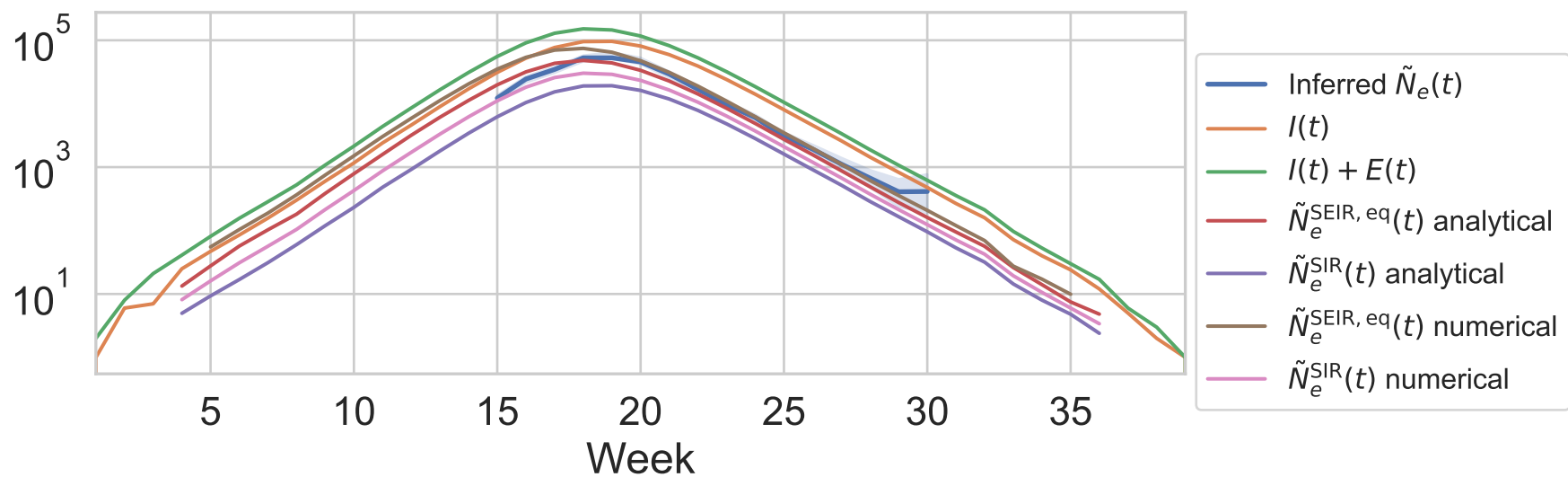

Supplement: S30 Fig — (Top) Muller plot of simulated infectious and exposed individuals’ lineage trajectories (simulations described in Methods). Infectious and exposed individuals are randomly assigned a lineage in week 11, and individuals that they transmit to are infected with the same lineage. The blue lineage before week 11 indicates the infectious and exposed individuals that existed before lineages were assigned. (Bottom) Comparison of the inferred Ne˜(t) using the lineage trajectories shown in the top panel to the number of infectious individuals I(t), the sum of the number of infectious and exposed individuals I(t)+ E(t), Eq 49 (SEIR model Ne˜(t)), and Eq 1 (SIR model Ne˜(t)) calculated analytically or numerically as described in the Methods. The numerical solutions give slightly higher Ne˜(t) as compared with the analytical solutions; however, the numerical solutions to the SEIR and SIR models bound the inferred Ne˜(t). (PDF) [file ppat.1012090.s033.pdf]

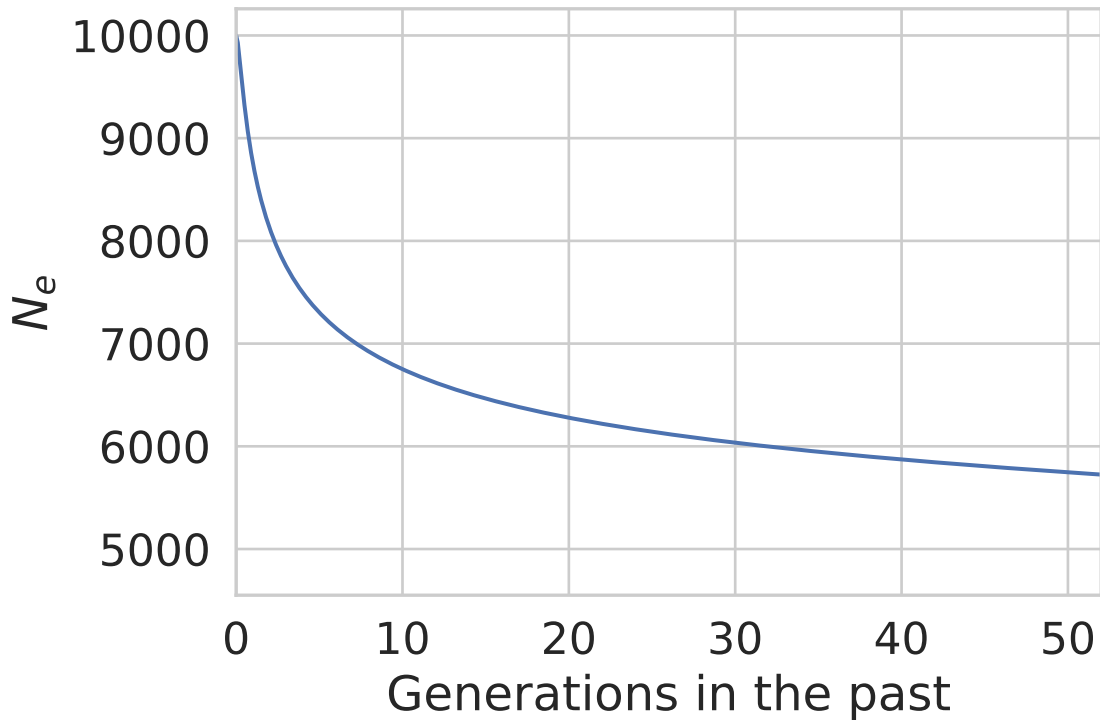

Supplement: S31 Fig — In this calculation, the effective population size in the absence of background selection is 104, the clock rate is 31 substitutions per year, and the generation time is 5.1 days. (PDF) [file ppat.1012090.s034.pdf]

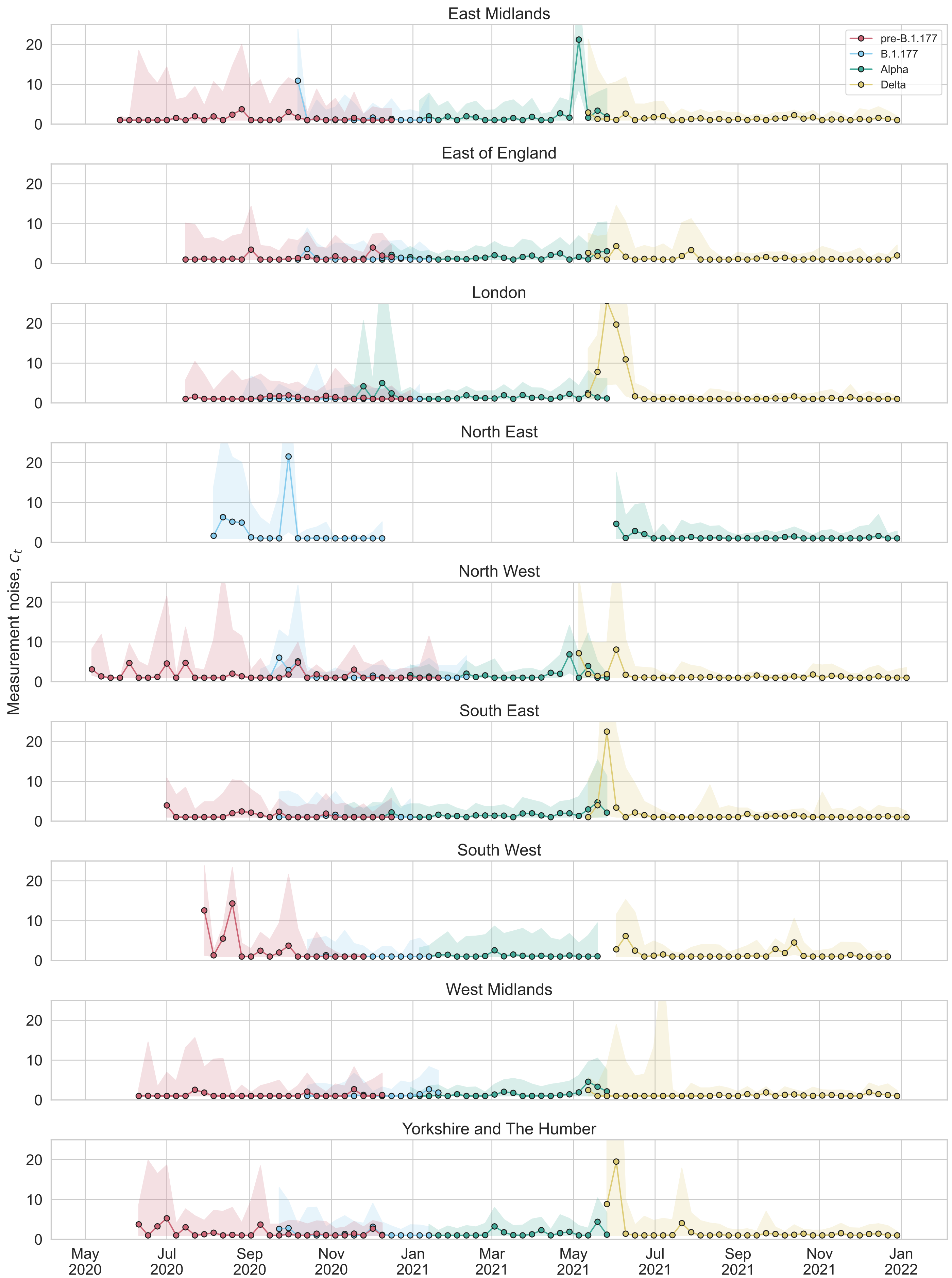

Supplement: S33 Fig — (PDF) [file ppat.1012090.s036.pdf]

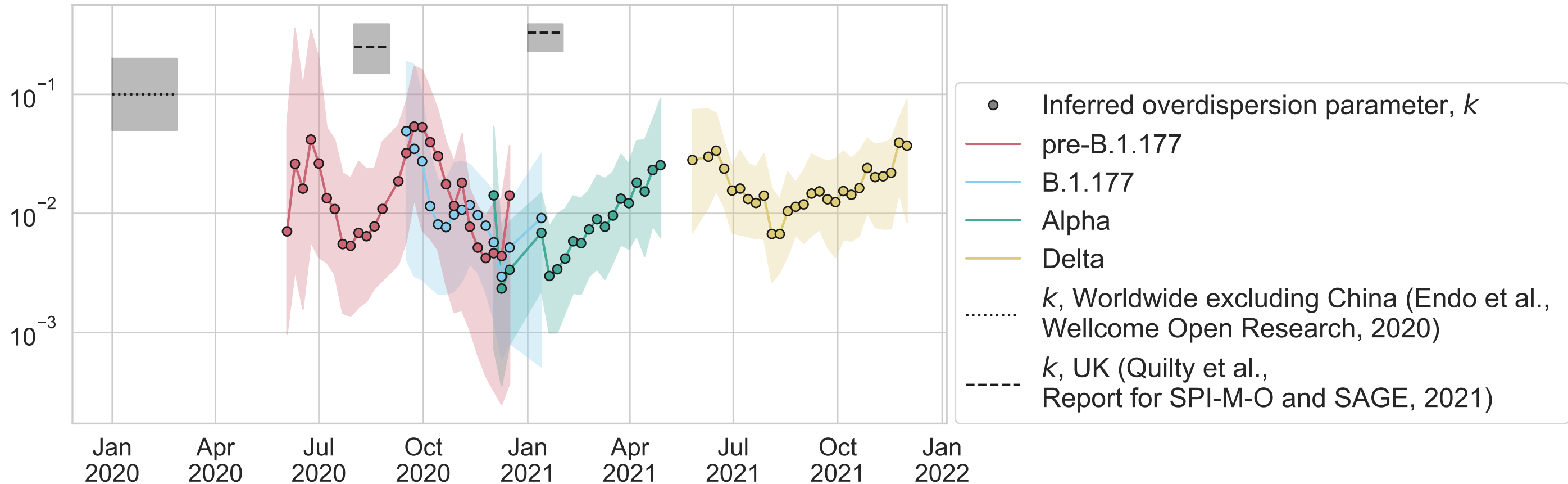

Supplement: S34 Fig — The circles show the inferred overdispersion parameter if we assume there is only superspreading and no deme structure. For the inferred overdispersion parameter, the estimated effective reproduction number in England by variant (see Methods) is used for Rt, and the ratio between the SIR model population size and the inferred effective population size is used for σ2. The shaded area for the inferred overdispersion parameter k gives an estimate of the error and is calculated by combining minimum or maximum values of the individual parameters; note that this does not correspond to a particular confidence interval. (PDF) [file ppat.1012090.s037.pdf]
