# Supplementary material for: Lineage frequency time series reveal elevated levels of genetic drift in SARS-CoV-2 transmission in England
Source: PLoS Pathog. 2024 Apr 15;20(4):e1012090. doi: 10.1371/journal.ppat.1012090 (PMC11045146; doi:10.1371/journal.ppat.1012090)
Supplement: S6 Fig — (PDF) [file ppat.1012090.s009.pdf]

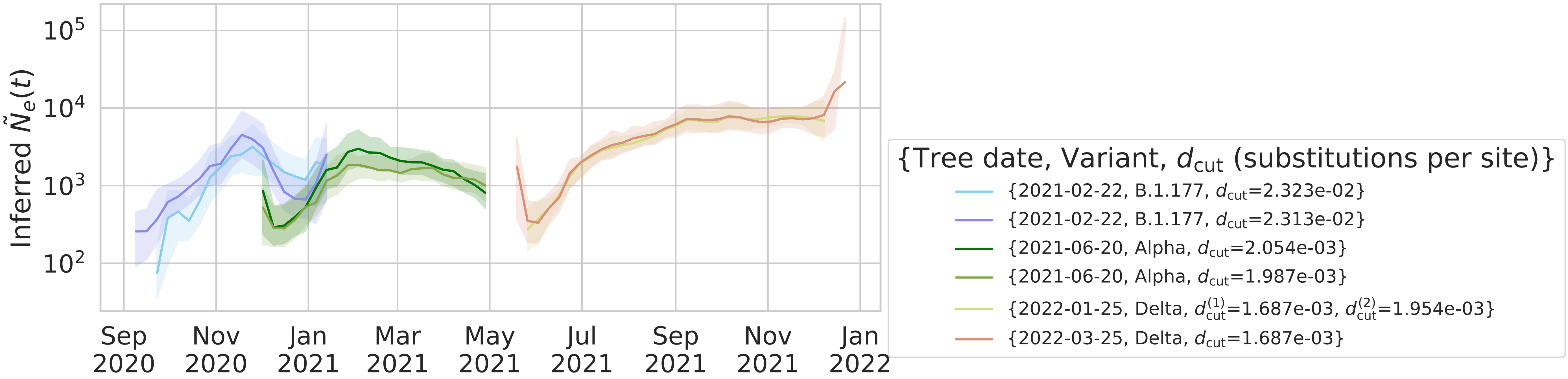

Inferred $\tilde{N}_e(t)$
10^5
10^4
10^3
10^2
Sep 2020
Nov 2020
Jan 2021
Mar 2021
May 2021
Jul 2021
Sep 2021
Nov 2021
Jan 2022
{Tree date, Variant, d_{cut} (substitutions per site)}
{2021-02-22, B.1.177, d_{cut}=2.323e-02}
{2021-02-22, B.1.177, d_{cut}=2.313e-02}
{2021-06-20, Alpha, d_{cut}=2.054e-03}
{2021-06-20, Alpha, d_{cut}=1.987e-03}
{2022-01-25, Delta, $d_{\mathrm{cut}}^{(1)}$=1.687e-03, $d_{\mathrm{cut}}^{(2)}$=1.954e-03}
{2022-03-25, Delta, d_{cut}=1.687e-03}
