# Supplementary material for: Lineage frequency time series reveal elevated levels of genetic drift in SARS-CoV-2 transmission in England
Source: PLoS Pathog. 2024 Apr 15;20(4):e1012090. doi: 10.1371/journal.ppat.1012090 (PMC11045146; doi:10.1371/journal.ppat.1012090)
Supplement: S24 Fig — The vertical dashed line indicates 105 which is the value above which results in the text were thrown away due to non-convergence (these only include values at 1011). (PDF) [file ppat.1012090.s027.pdf]

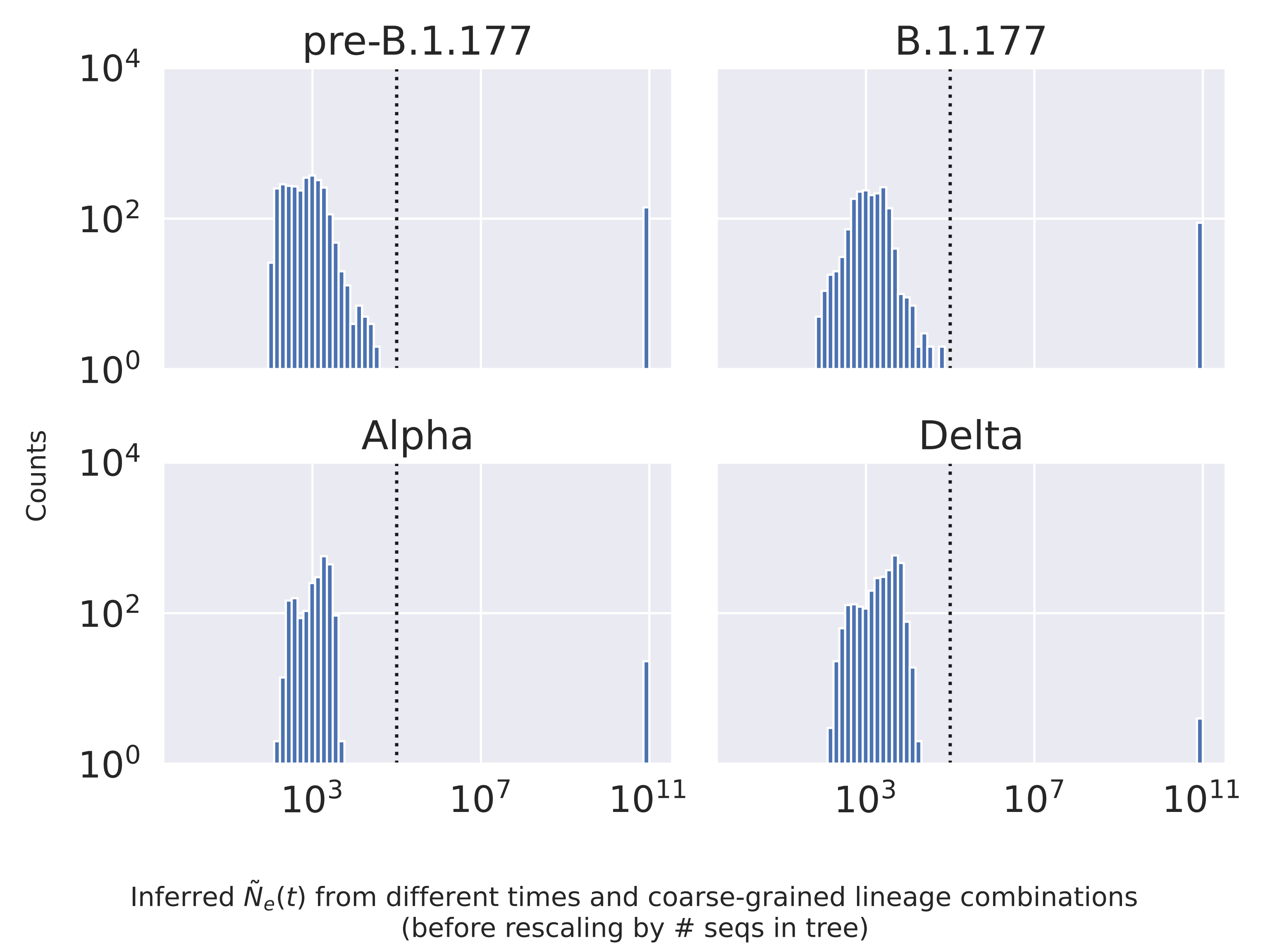
pre-B.1.177
B.1.177
Alpha
Delta
Counts
10^4
10^2
10^0
10^3
10^7
10^{11}
Inferred $\tilde{N}_e(t)$ from different times and coarse-grained lineage combinations
(before rescaling by # seqs in tree)
