# Supplementary material for: Lineage frequency time series reveal elevated levels of genetic drift in SARS-CoV-2 transmission in England
Source: PLoS Pathog. 2024 Apr 15;20(4):e1012090. doi: 10.1371/journal.ppat.1012090 (PMC11045146; doi:10.1371/journal.ppat.1012090)
Supplement: S19 Fig — (Top panels) Inferred Ne˜(t) of pre-B.1.177 lineages, B.1.177, Alpha, and Delta for each region of England. The inferred Ne˜(t) for England as a whole is shown for reference. Shaded regions show 95% confidence intervals (see Methods). (Bottom panels) The ratio between the inferred Ne˜(t) of England and that of the region for each variant. A horizontal dashed line indicates a ratio of 1 (i.e. Ne˜(t) is the same in that region of England and England as a whole). Shared regions show the minimum and maximum possible values of the ratio from the combined error intervals of the numerator and denominator (thus, not corresponding to a specific confidence interval range). (PDF) [file ppat.1012090.s022.pdf]

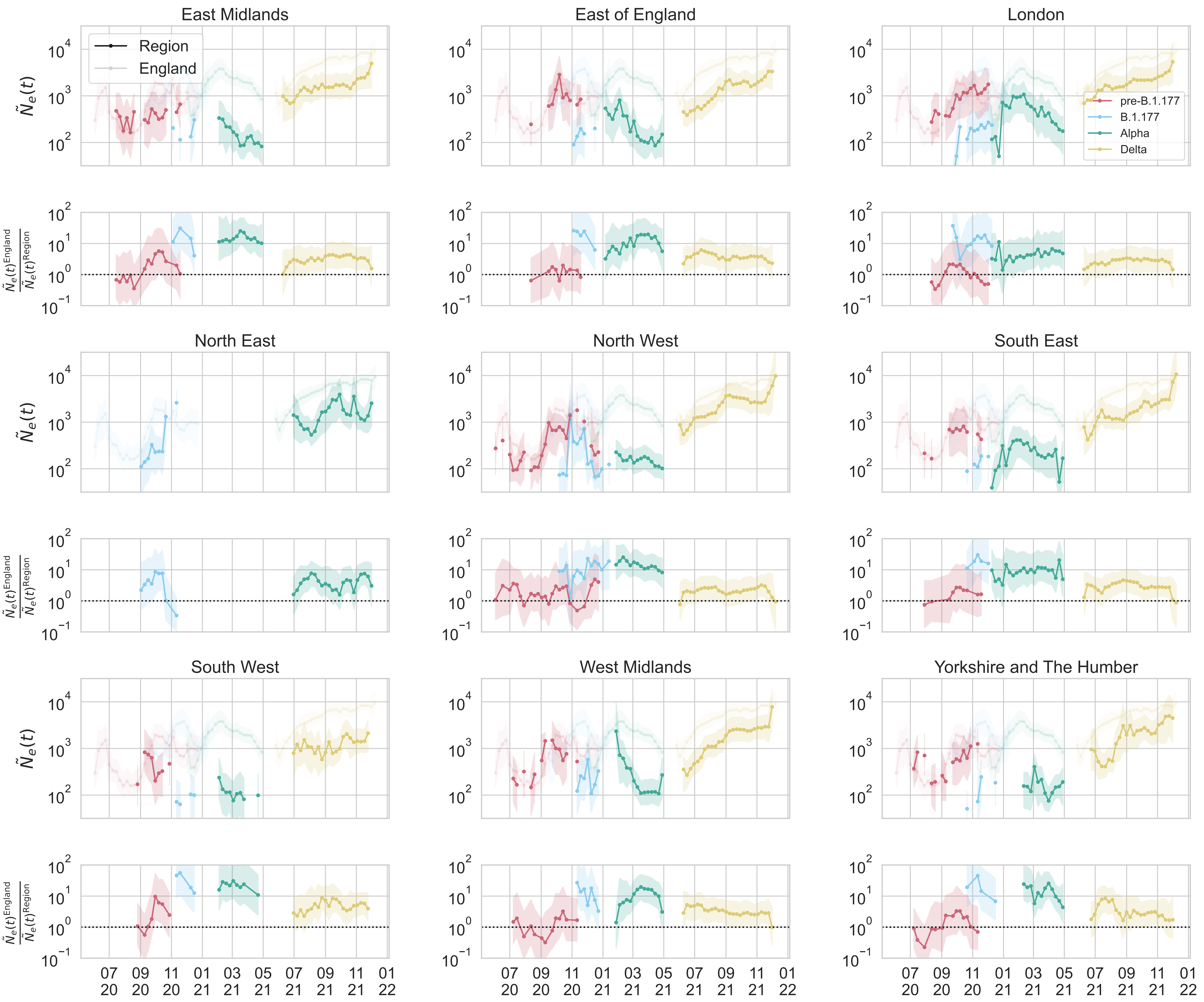

East Midlands
East of England
London
North East
North West
South East
South West
West Midlands
Yorkshire and The Humber
Region
England
pre-B.1.177
B.1.177
Alpha
Delta
$\tilde{N}_e(t)$
$\frac{\tilde{N}_e(t)^{\mathrm{England}}}{\tilde{N}_e(t)^{\mathrm{Region}}}$
10^{-1}
10^0
10^1
10^2
10^3
10^4
07 20
09 20
11 20
01 21
03 21
05 21
07 21
09 21
11 21
01 22
