# Supplementary material for: Lineage frequency time series reveal elevated levels of genetic drift in SARS-CoV-2 transmission in England
Source: PLoS Pathog. 2024 Apr 15;20(4):e1012090. doi: 10.1371/journal.ppat.1012090 (PMC11045146; doi:10.1371/journal.ppat.1012090)
Supplement: S32 Fig — (PDF) [file ppat.1012090.s035.pdf]

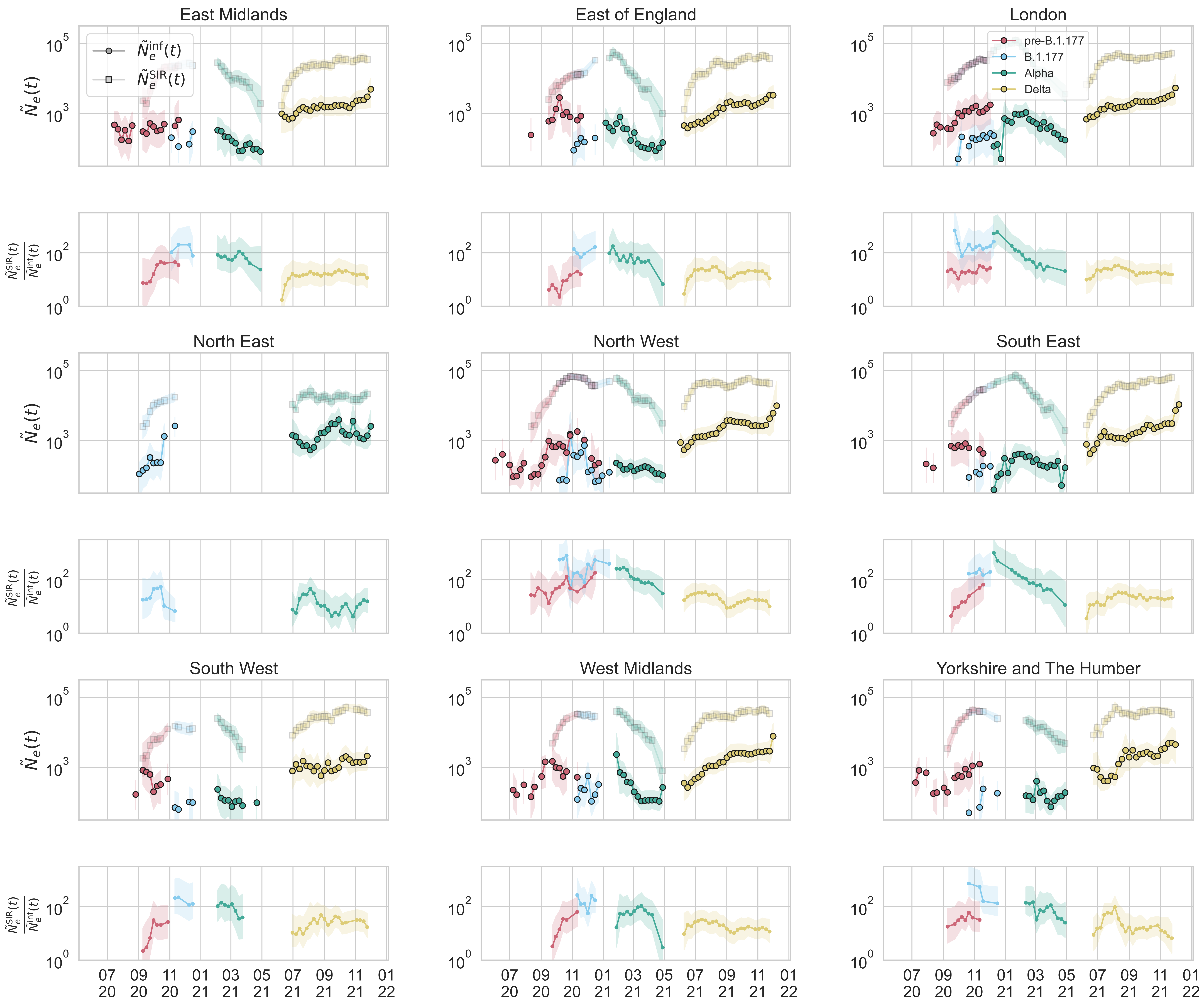
East Midlands
East of England
London
North East
North West
South East
South West
West Midlands
Yorkshire and The Humber
$\tilde{N}_e^{\mathrm{inf}}(t)$
$\tilde{N}_e^{\mathrm{SIR}}(t)$
pre-B.1.177
B.1.177
Alpha
Delta
$\tilde{N}_e(t)$
$\frac{\tilde{N}_e^{\mathrm{SIR}}(t)}{\tilde{N}_e^{\mathrm{inf}}(t)}$
10^0
10^2
10^3
10^5
07 20
09 20
11 20
01 21
03 21
05 21
07 21
09 21
11 21
01 22
