# Supplementary material for: Lineage frequency time series reveal elevated levels of genetic drift in SARS-CoV-2 transmission in England
Source: PLoS Pathog. 2024 Apr 15;20(4):e1012090. doi: 10.1371/journal.ppat.1012090 (PMC11045146; doi:10.1371/journal.ppat.1012090)
Supplement: Attachment — Submitted filename: Response to reviewers.pdf [file ppat.1012090.s038.pdf]

Note that the line numbers below refer to the manuscript pdf that has tracked changes.

Part I - Summary
Please use this section to discuss strengths/weaknesses of study, novelty/significance, general execution and scholarship.

Reviewer #1: see below

Reviewer #2: This manuscript uses UK COVID-19 survey data to estimate the "effective population size" of the SARS-CoV-2 epidemic in the UK, and finds that it is roughly 100x smaller than the number of cases. Concretely, the fluctuations in the observed frequencies of different lineages over time are far larger than expected. While some of the fluctuations are due to fluctuating biases in sampling, for the most part they compound over time, indicating that they are primarily due to fluctuations in the true underlying frequencies of the lineages. I think this paper is very exciting, both for its results and as an example of the new kinds of epidemiological analyses that can be done with large sequence surveys. Overall, I think that the scholarship is very solid, although I do I have some suggestions that the authors may want to consider.
I guess the issue with simultaneously inferring selection and drift is computational, in that it breaks the nice factorized form of the approximate likelihood. (As I mentioned, I think that the importance of this form should be pointed out earlier in the manuscript.) If this makes it impractical, then I think another option would be to follow the same s inference procedures as currently used, but discard lineages based on the magnitude of the point estimates of s or s * (delta t), where delta t is the time interval over which the lineage is observed, rather than a statistical significance test. Or one could even use a direct comparison of the inferred magnitude of allele change due to selection vs the expected allele frequency change based on the inferred N_e and keep only those lineages where the former was small compared to the latter.

As the reviewer has suggested, we have updated the procedure of discarding lineages to be based off of the magnitude of the inferred fitness, s, rather than a statistical significance test. We discarded lineages whose absolute value of s were above the 50th ($|s|>0.09$), 75th ($|s|>0.16$), and 90th ($|s|>0.27$) percentiles. The inferred effective population size for all three of these cases was similar to when not discarding lineages (Figure R1). This has been updated in the text in lines 259-268 and the section in the methods describing the calculation of statistical significance and the second round of fitness inference have been removed (lines 851-872) as they are no longer relevant.

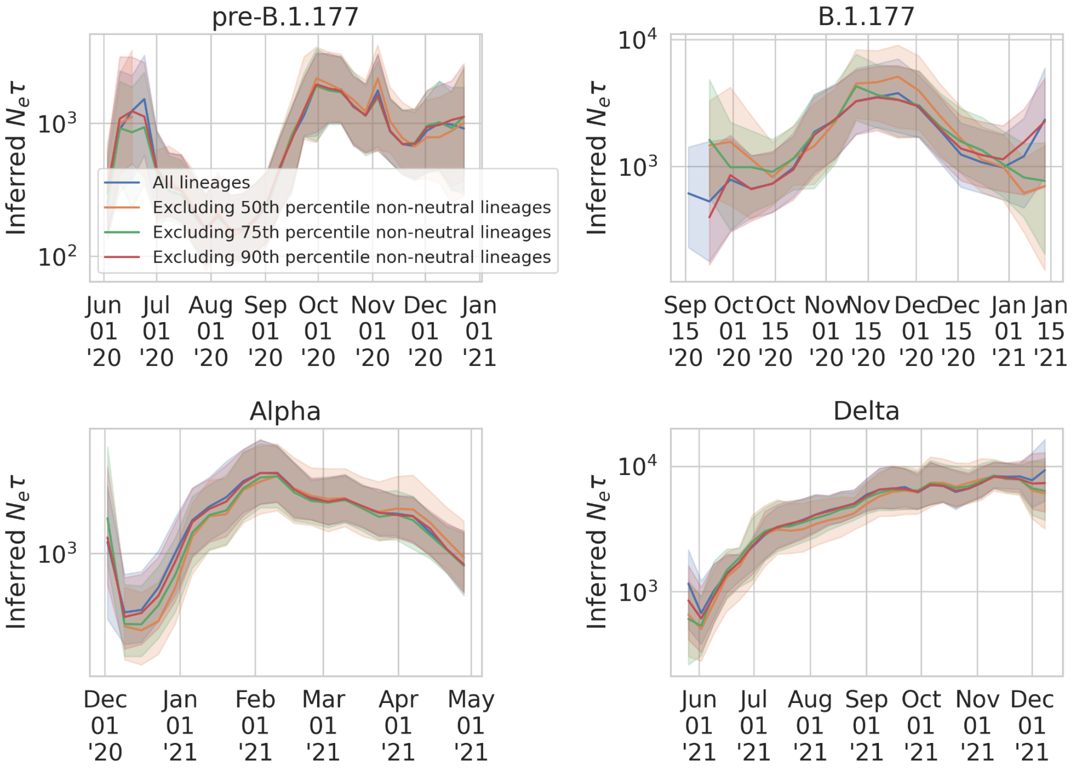

Figure R1: The inferred effective population size when excluding beneficial lineages whose inferred absolute fitness value are above the 50th (|s|>0.09), 75th (|s|>0.16), and 90th (|s|>0.27) percentiles compared to that when all lineages are included.

Reviewer #3: The authors present an approach to infer genetic drift and measurement noise from time-series lineage frequency data using genomic sequence and surveilance data from COG-UK up to December 2021. Measurement noise is overdispersed and the strength of overdispersion varies over time. The authors show that these two types of noise leave distinct footprints in the data. The method is convincing, presented in a clear way, and validated on simulated data.

The inference for SARS-CoV-2 is performed in 4 distinct major lineages to limit the effect of selection. The authors map the effective population size as a function of time for each major lineage. They show that the inferred effective population size is orders of magnitude smaller than what one would expect from an SIR model using case data. The authors discuss two potential mechanism that might explain the high level of genetic drift: super-spreading and deme structure within the population.

The authors have used a careful method to infer an important evolutionary parameter of SARS-CoV-2. However, their results may be more strongly linked to the evolutionary context.

Part II – Major Issues: Key Experiments Required for Acceptance
Please use this section to detail the key new experiments or modifications of existing experiments that should be absolutely required to validate study conclusions.

Generally, there should be no more than 3 such required experiments or major modifications for a "Major Revision" recommendation. If more than 3 experiments are necessary to validate the study conclusions, then you are encouraged to recommend "Reject".

Reviewer #1: Yu et al.

Reviewed for PLOS Pathogens

In this manuscript, the authors propose an approach to jointly estimate parameters related to genetic drift and to sampling variance using time-series data. They apply the approach to CoV-2 sequence collected in the UK in 2020-2021. They argue that the degree of observed genetic drift is higher than may be expected.

I see a number of population genetic oddities in this work. While the authors jump through many hoops in an attempt to justify the assumption of neutrality within lineages in order to simplify interpretations, these efforts are unsatisfactory. As degrees of genetic drift are being gleaned from changes in allele frequency, and given that the CoV-2 genome is essentially entirely functional, this neutrality assumption (even within strains) is unjustified. While the authors perform a simple test to examine deterministic allele frequency changes in an effort to identify 'possibly non-neutral' effects, this is problematic as the authors seem to be searching for the sort of deterministic increases in allele frequency associated with selective sweeps only. However, the vastly more common type of selection will be purifying in nature, and thus they will be unable to observe 'deterministic' changes in allele frequency amongst rare variants. As such, the vast majority of their sequence data will be impacted by purifying selection as well as the resulting background selection effects. They simply can not justify neglecting these major evolutionary contributors. This negative selection will of course be occurring in all 'strains' and 'lineages', and will to a first approximation give the appearance of a reduced effective population size. Given that the main conclusion of this manuscript is that genetic drift may be stronger than one would expect given "the number of positive individuals" observed (though the basis for this claim is still quite unclear to me), I see no reason to think that neglected purifying selection effects would not be the main contributor to the observation. The authors may want to study this recent AREES Review to consider these expectations (Charlesworth & Jensen, 2021, 'Effects of selection at linked sites on patterns of genetic variability').

In this work, we developed a statistical method to infer the effective population size that a well-mixed Wright-Fisher population would have to have to generate the allele frequency fluctuations seen in SARS-CoV-2 genomic data. This classic definition of effective population size gives us a measure for the strength of genetic drift (no matter how well the allele frequency trajectories can be described by a Wright-Fisher model). A separate question, which we speculate upon, is what mechanisms can generate this reduced effective population size. In

response to the reviewer's comments, we have now moved the section about potential mechanisms that can generate reduced effective population size (superspreading and deme structure) from the Results section into the Discussion section.

The reviewer raises a very valid point that background selection can decrease the effective population size, and should be discussed in our manuscript. Based on the empirical estimates of the distribution of fitness effects (Bloom and Neher, bioRxiv, 2023, which are consistent with experimental measurements; see Dadonaite et al., 2023; Starr et al., 2022; Iketani et al., 2022; Flynn et al., 2023) and two new analyses that we describe below, we do not believe that background selection is sufficiently strong in this system to explain a substantial fraction of the inferred decrease in effective population size that we observe.

We first estimated the magnitude by which we expect the effective population size to be decreased due to background selection given the empirically estimated distribution of fitness effects (Bloom and Neher, bioRxiv, 2023; reproduced in Figure R2) using simulations. For these simulations, we modified the neutral simulations in the following ways. Initially, each individual has a fitness drawn from the empirical distribution of deleterious fitness effects. Additionally, each individual forms a single lineage. To model selection, the probability of being drawn in the multinomial distribution is weighted by e^s, where s is the fitness coefficient. Mutations occur on the background of each individual in each generation with probability 0.01 and the mutants have a fitness that is the sum of that of the parent and a newly drawn fitness from the distribution of deleterious fitness effects. The burn-in period ends when the number of lineages reaches the threshold of 100 lineages, and recording begins. No new lineages are created in the simulation, so lineages are defined as the descendants of the individuals that are initially in the simulation.

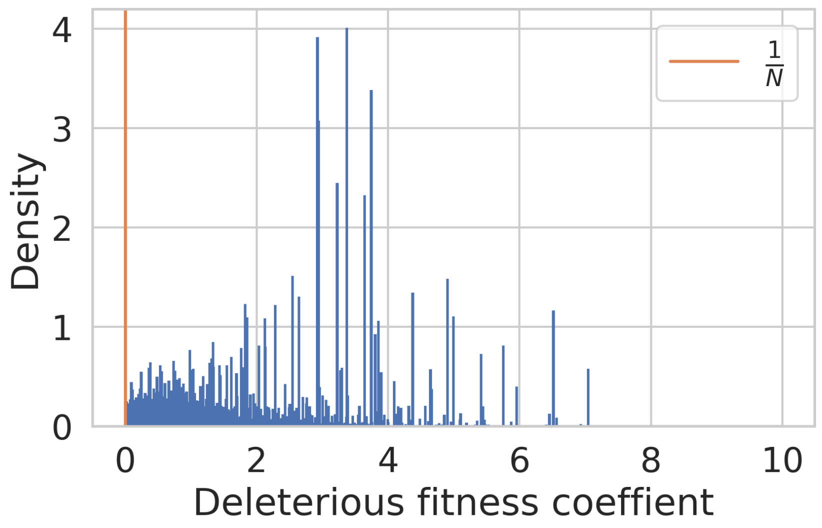

Figure R2: The distribution of deleterious fitness effects from Bloom and Neher, bioRxiv, 2023. The orange vertical line indicates 1/N, which is the threshold in fitness above which selection dominates over genetic drift. Here, N is set to 10^4, which is the order of magnitude of the census population size of SARS-CoV-2 in England.

Using the simulated data, we found that the inferred effective population size is consistent with the true effective population size (Figure R3) to within error bars. The deviations only occur when there is insufficient data due to there being few lineages.

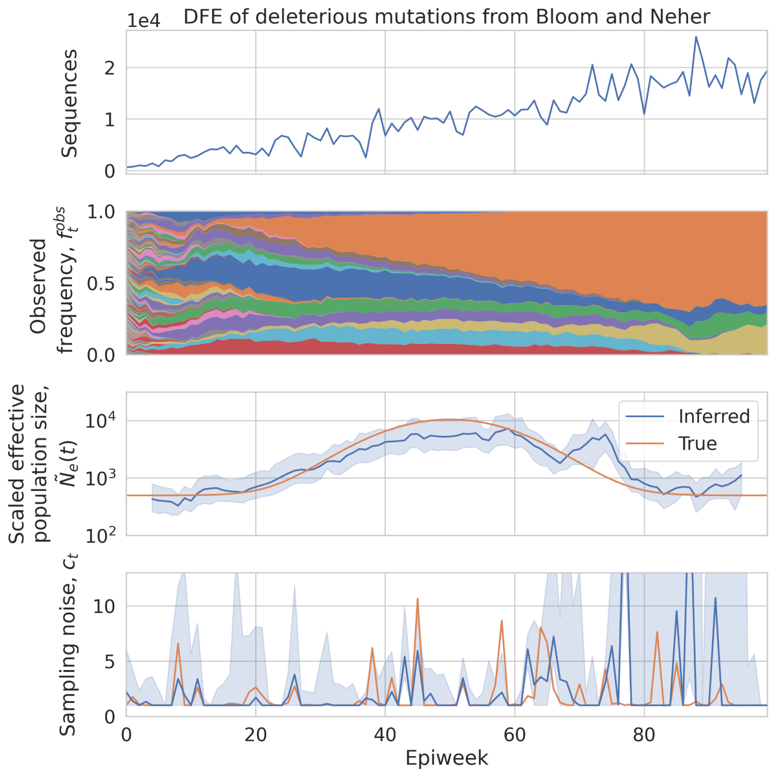

Figure R3: Simulated lineage frequency dynamics where deleterious mutations occur at rate 0.01/genome/generation and the distribution of deleterious fitness effects is taken from the empirically estimated values in Bloom and Neher, bioRxiv, 2023. The inferred effective population size and measurement noise are shown.

Since we had modified the simulations from the simulations, we also performed a control to check that when there are only neutral mutations, the simulated data still gives the correct inferred effective population size; this was indeed the case to within the error bars (Figure R4) . Importantly, the ratio of the inferred effective population size for the simulation with the deleterious distribution of fitness effects and neutral mutations was no lower than 0.5 (Figure R5).

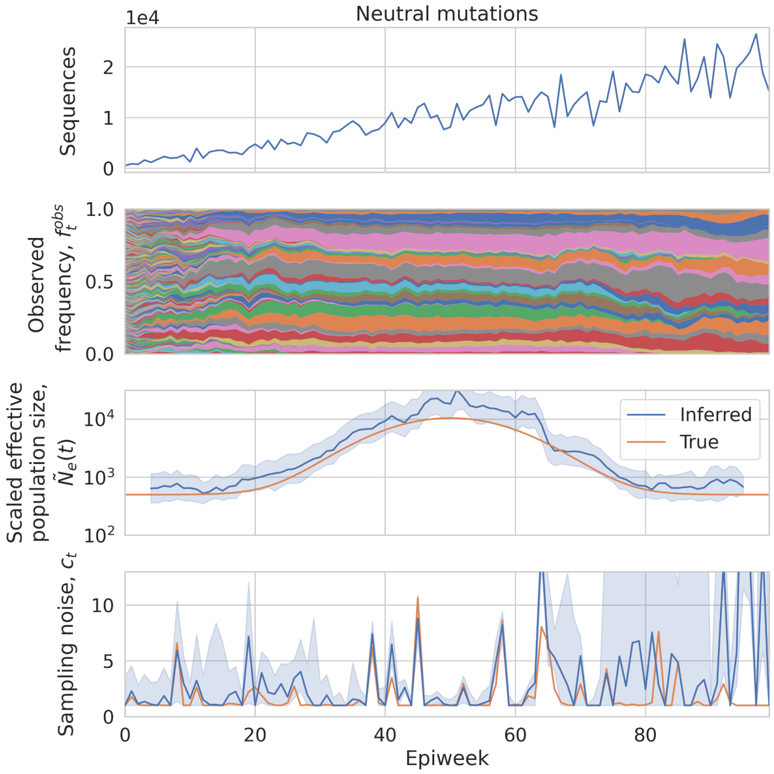

Figure R4: The same simulation as in Figure R3 but as a control, where the fitness of new mutations is always 0. The inferred effective population size and measurement noise are shown.

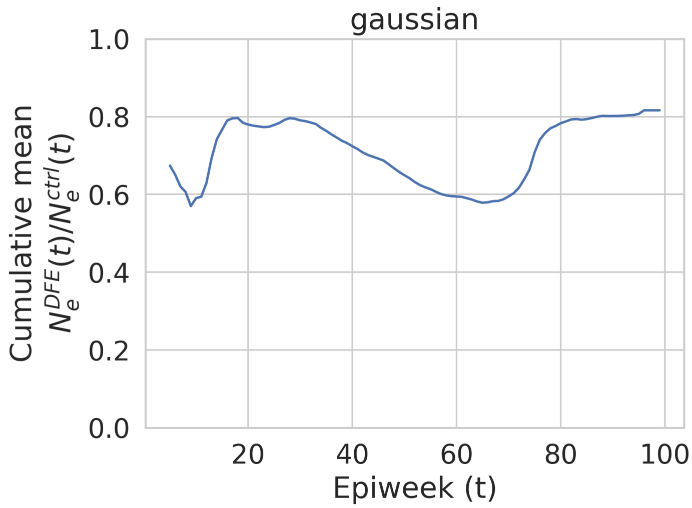

Figure R5: The cumulative mean ratio of the point estimates of the inferred effective population size in the simulations using the empirical distribution of deleterious fitness effects and the neutral simulations.

We also estimated the magnitude by which we expect the effective population size to be decreased due to background selection given the empirically estimated distribution of fitness effects using analytical theory. Most theoretical studies on background selection consider strongly deleterious mutations with a single negative fitness value and assume that deleterious mutants quickly die out so that multiple mutations don't occur in the same background (Charlesworth and Jensen, Annual Review of Ecology, Evolution, and Systematics, 2021). However, in this case we need to consider a distribution of fitness effects and the possibility of mutants with different fitnesses existing simultaneously. As such, we used Equation 8 derived from Nicolaisen and Desai, Genetics, 2013 for the effective population size in the presence of deleterious mutations with a distribution of fitness effects, assuming a constant mutation rate and no recombination:

$$N_e \approx N exp\left[- \int_{1/N}^{\infty} \frac{\mu}{s} \left(1 - e^{-st}\right)^2 \rho(s) ds \right] \quad (1)$$

where μ is the deleterious mutation rate per generation per genome, $\rho(s)$ is the deleterious distribution of fitness effects (i.e. the fitness effect is -s), t is time in generations into the past, and N is the census population size. Assuming no recombination is a conservative assumption, as recombination mitigates the effects of background selection (Charlesworth and Jensen, Annual Review of Ecology, Evolution, and Systematics, 2021).

Using the empirically estimated distribution of fitness effects from Bloom and Neher, bioRxiv, 2023 and the clock rate of 31 substitutions per year (Nextstrain SARS-CoV-2 GISAID build on August 7, 2023, which is an upper bound estimate on the deleterious mutation rate that affects the effective population size), a generation time of 5.1 days (Hart et al., eLife, 2022), and a census population size of 10^4 (order of magnitude of census population size of SARS-CoV-2 in England), we estimated that the effective population size would be decreased by at most a factor of 2 at times far into the past, and less in more recent times, due to background selection (Figure R6). The above formula was derived assuming strong selection (s>>1/N) for the bulk of negative mutations, which we see from the distribution of fitness effects does hold (Figure R2). This result is consistent with what we found in the simulations.

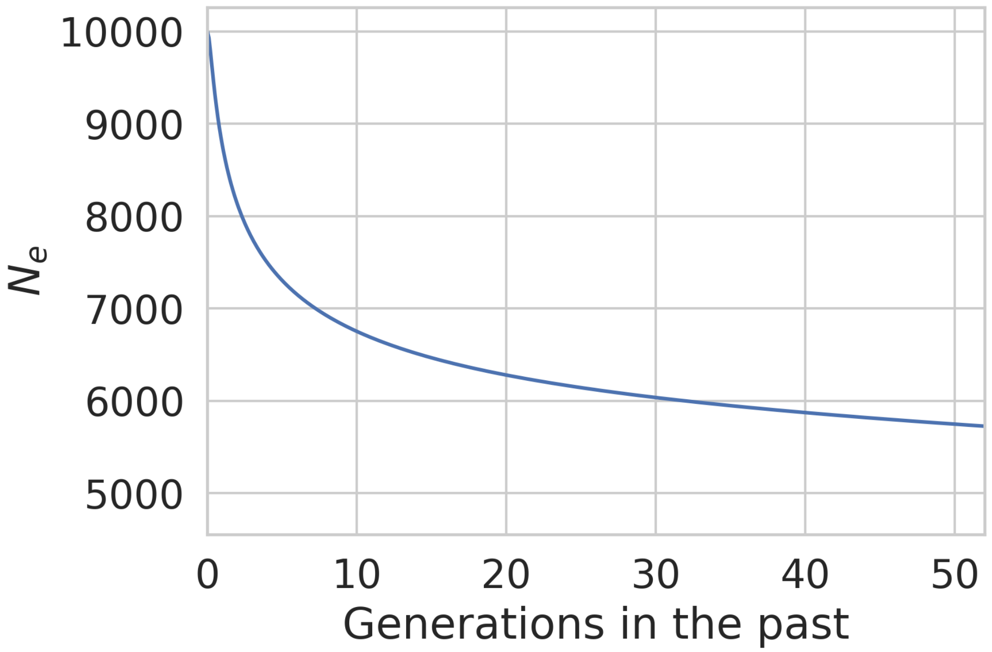

Figure R6: The effective population size in the presence of deleterious mutations with a distribution of fitness effects, assuming a constant mutation rate and no recombination, calculated using Equation 1. The census population size is 10^4, the clock rate is 31 substitutions per year, the generation time is 5.1 days, and the distribution of deleterious fitness effects is taken from Bloom and Neher, bioRxiv, 2023.

Thus, while background selection can in general decrease the effective population size, in this system it can explain at most a factor of 2 reduction in the effective population size, which is a small fraction of the observed reduction of two orders of magnitude. This analysis of background selection using simulations and analytical theory has been added to the manuscript in the results (lines 272-284) and methods sections (lines 759-768) and the Supplementary Information (lines 1241-1264), and we have also cited Charlesworth et al., 2021.

As another of many possible examples in this regard, the authors also neglect gene flow, which is likely considerable in this human pathogen, and which would also serve to inflate estimates of genetic drift when ignored.

We agree that gene flow both within the population and in or out of the population is important for SARS-CoV-2 but we believe this point has already been addressed in the first version of the manuscript.

The deme model described in the Discussion investigates gene flow and demographic structure within the population, and we find that jackpot events in demes will lead to a strongly decreased effective population size. Importantly, in the absence of jackpot events, we do not expect gene flow within the population to affect the effective population size (Charlesworth et al., Nature Genetics Review, 2009). This point has been clarified in the Discussion in lines 490-493.

Gene flow in and out of the population was previously addressed in the discussion in lines 488-490: “Migration can substantially change frequencies that are locally rare, but we expect importations to only weakly influence the frequency fluctuations of abundant variants, on which we have focused in this work.”

The authors propose other unsupported assumptions to further simplify, and justify this as "we consider the extreme situation where only one mechanism at a time is driving the dynamics". Again, there will simply be no empirical data in which only one evolutionary force is acting; at a bare minimum this CoV-2 data is being shaped by mutation, recombination, genuine genetic drift as driven by population size change and structure, purifying selection, background selection, 'superspreading', etc. The authors may want to consider the recent PLOS Pathogens Review on general evolutionary dynamics in CoV-2 (Terbot et al., 2023, 'Developing an appropriate evolutionary baseline model for the study of SARS-CoV-2 patient samples'). The authors may also want to consider this more generalized PLOS Biology Commentary, written by many of the leading thinkers in evolutionary inference, about the dangers of neglecting known-to-be-contributing evolutionary processes when performing such estimation (Johri et al., 2022, 'Recommendations for improving statistical inference in population genomics').

Our motivation for considering each mechanism in turn was not to suggest that only a single mechanism was at play, but rather to gain intuition about how different mechanisms could affect the effective population size. We wondered whether the reduction in the effective population size could be entirely explained by superspreading, but found that superspreading was unlikely to be able to fully explain the observed reduction in effective population size. We found that host deme structure could be strong enough to explain the observed reduction in effective population size. However, we now emphasize more strongly that the jackpots-in-deme mechanism is merely a possibility, as we cannot rule out from our data and approaches that other mechanisms are at play. We have clarified our motivation described here in the text in lines 349-351, which is now part of the Discussion rather than the Results section.

In lines 447-515 in the Discussion, we have also added a section summarizing how we have addressed other relevant evolutionary forces and why we think that genetic drift is driving a large fraction of the magnitude of the effective population size that we observe, where we have cited Johri et al., 2022.

As a final point, it is entirely unclear why the authors are describing effective population size under a randomly mating Wright-Fisher model at all (and why test simulations in particular are being performed under this clearly inappropriate model), when they themselves are making a case for skewed offspring distributions via 'superspreading'. There are of course many developed frameworks under generalized Moran models and multiple merger coalescent theory that would be much more appropriate for a viral population of this sort.

Our goal was to quantify the strength of the lineage frequency fluctuations in SARS-CoV-2 transmission. By using the standard definition of an effective population size of a Wright-Fisher

population, we used a well-known standard measure for the strength of lineage frequency fluctuations. This quantity reflects the mean squared deviation of the fluctuations per unit time.

Lineage frequency fluctuations are asymptotically described by the Wright-Fisher model, as long as the offspring distribution has a finite mean and variance. Empirically measured SARS-CoV-2 offspring distributions that take into account superspreaders (see references in Table S1 in manuscript) have been described by a negative binomial distribution, which has a finite mean and variance and thus can be described by the Wright-Fisher model.

To further show that a Wright-Fisher model can capture the strength of genetic drift for SARS-CoV-2 based on the empirically measured offspring number distributions, we simulated the lineage frequency trajectories using a census population size of $3.4*10^5$ and the empirically measured offspring number distribution from Endo et al., 2020 which is described by a negative binomial distribution with offspring number variance of 65 (basic reproduction number 2.5, dispersion parameter 0.1). The simulations were implemented in the same way as the neutral simulations for validating the method described in the paper, with the following modifications. The number of offspring for each parent individual are drawn from a negative binomial distribution with mean 1 and variance 65. Then, the number of offspring is summed for all parent individuals in a lineage. The frequency of each lineage is calculated by dividing by the total number of offspring in the population and then used as the frequency input to the multinomial for drawing the true number of individuals in each lineage in the next time point.

We then inferred the effective population size using our method on the simulated data and found that it indeed predicts the expected effective population size (Figure R7). This demonstrates that our inference of the effective population size is robust to the kind of broad offspring distributions that have been estimated for SARS-CoV-2.

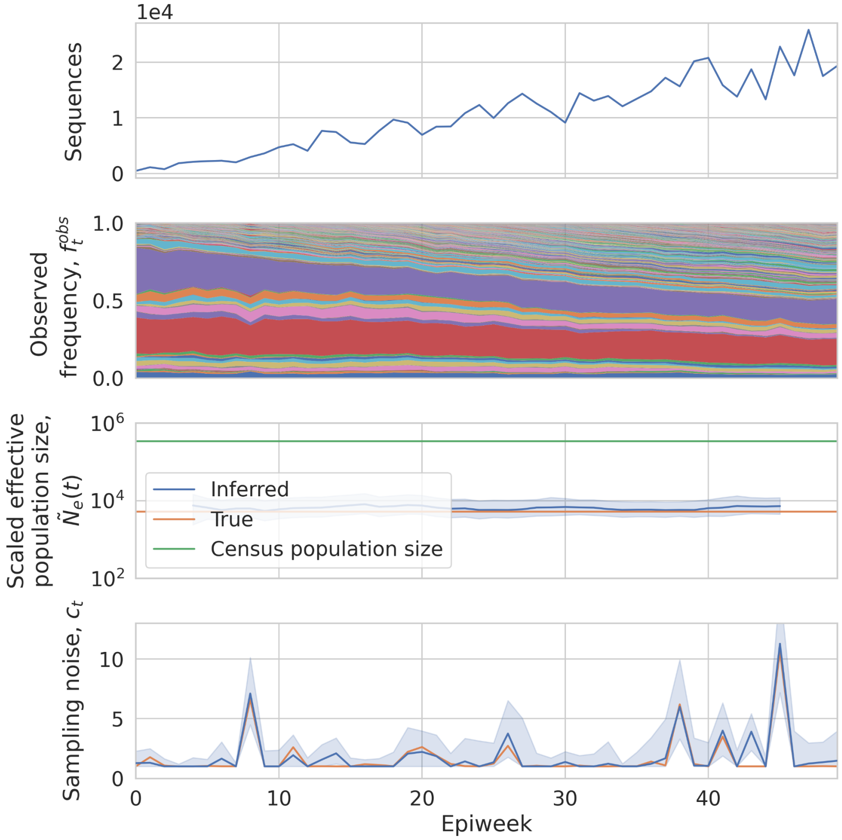

Figure R7: Simulated lineage frequency dynamics with an offspring number distribution described by a negative binomial distribution with variance 65 (inferred by Endo et al., 2020) and the inferred effective population size and measurement noise.

We cannot exclude the possibility that the empirically measured offspring distributions are biased and that the true offspring distribution is even more extremely heavy-tailed. However, even in this case, the use of a Wright-Fisher model and the effective population size are still informative for quantifying the magnitude of fluctuations and comparing between estimates. It tells us the effective population size that a well-mixed model would have to have to generate the magnitude of the fluctuations that we observe (Charlesworth, Nature Reviews Genetics, 2009).

We have clarified the motivation for using a Wright-Fisher model in lines 113-115.

In summary, the neglect of fundamental evolutionary processes and population genetic considerations makes this work unsuitable for publication.

Reviewer #2: 1. It would be nice to do more in the way of model checking, i.e., quantifying how well the fitted model matches the observed data. Some possible plots (maybe just as SI):
a. Scatter plot of $(\sqrt{f_{t+1} - \sqrt{f_t})$ vs $f(t)$.
b. Histogram of $(\sqrt{f_{t+1} - \sqrt{f_t}) / \sqrt{\tilde N_e(t)}$. This should be approximately normal, right?

c. Scaling of the mean squared change in the square root of frequency with the time elapsed. (Again, this could be normalized by the estimated $\tilde N_e(t)$ to allow data from different times to be averaged together.) This should be linear, right? (And with a known slope.) Saturating behavior would correspond to measurement error, and super-linear behavior could be due to selection or correlations in local transmission network structure. Maybe this is too messy to say anything definitive, but even if that's the case, it's nice to show it. Another way to approach this might be with a scatter plot of $f_{t+2} - f_{t+1}$ vs $f_{t+1} - f_t$.
Just throwing ideas out there, but I do think it's important to have some figures somewhere that show what features of the data the model is capturing and what features might point to other dynamics beyond the model.

We have created the plots as suggested:

The scatter plot of $(\sqrt{f_{t+1} - \sqrt{f_t})$ vs $f(t)$ is centered on 0 with a scatter that visually looks Gaussian, which is what we would expect in the model (Figure R8).

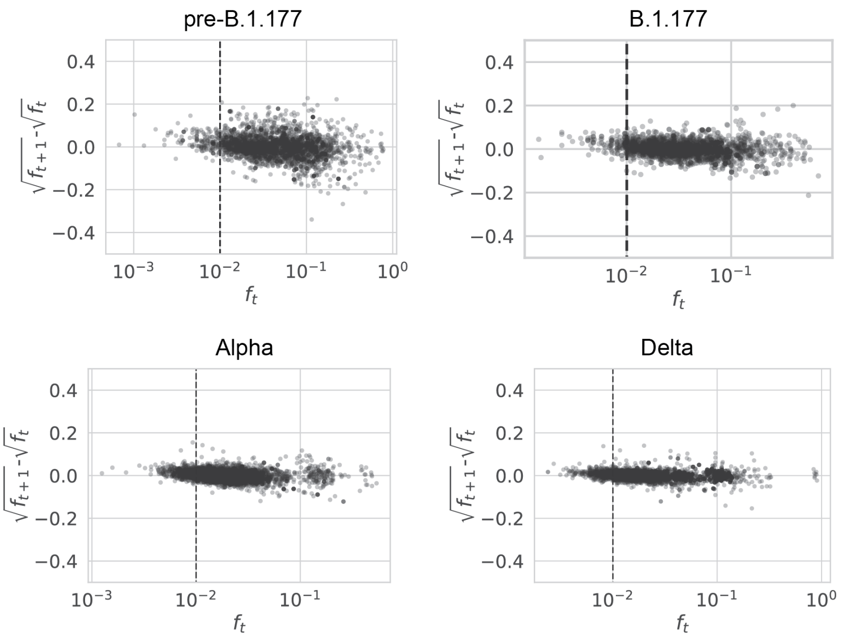

Figure R8: Scatter plot of $(\sqrt{f_{t+1} - \sqrt{f_t})$ vs $f(t)$ by variant. The vertical dashed line indicates 10^{-2} which is the minimum frequency for being a coarse-grained lineage in our method (lineages with lower frequency are combined to create coarse-grained lineages).

The Gaussian distribution of the square root frequency displacements can be seen more clearly in the histogram of $(\sqrt{f_{t+1} - \sqrt{f_t}) / \sqrt{\kappa_{t,t+1}}$, where $\kappa_{t,t+1}=c_t/{4M_t} + c_{t+1}/{4M_{t+1}}+1/{4 \tilde N_e(t)}$ (Figure R9).

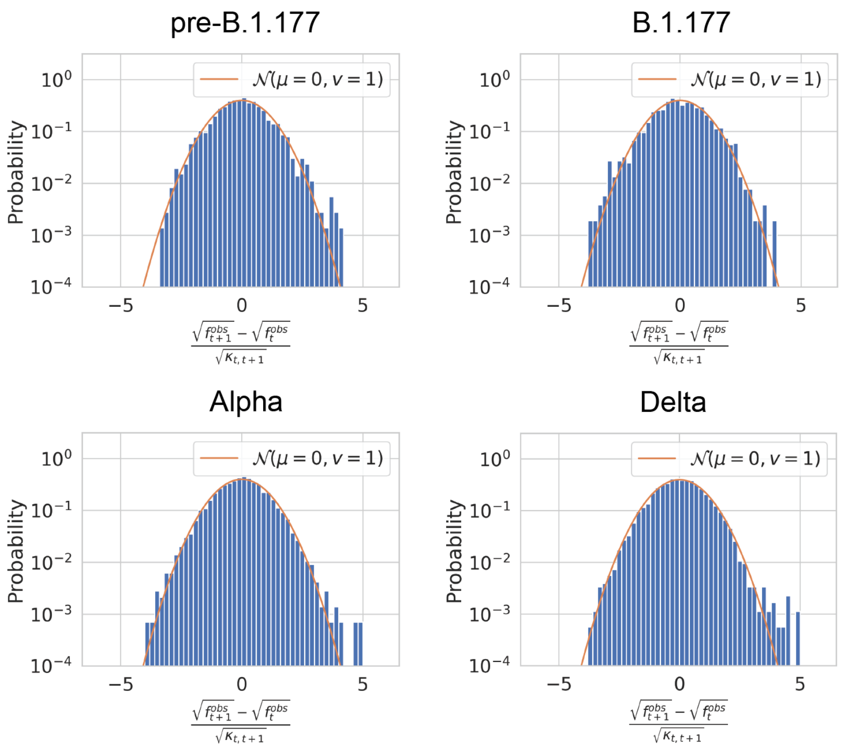

Figure R9: The distribution of the square root frequency displacements as shown by a histogram of $(\sqrt{f_{t+1}} - \sqrt{f_t}) / \sqrt{\kappa_{t,t+1}}$. The orange line indicates a gaussian distribution with mean 0 and variance 1, which is the expectation from our model.

Finally, we plotted the mean squared displacement of the square root frequencies normalized by the inferred strength of measurement noise c_t, the number of sequences, and the inferred effective population size. The mean squared displacement estimated using the point estimates of the inferred parameters is linear, but often with a slope larger than expected from the model; however, when we use the lower and upper bounds of c_t, the range of slopes encompassed by the data almost always includes the expected slope, with the exception being Delta (Figure R10). A higher slope than expected could indicate correlations in the transmission network (which would arise, for instance, from the host deme model that we introduced in the manuscript) or evidence of selection.

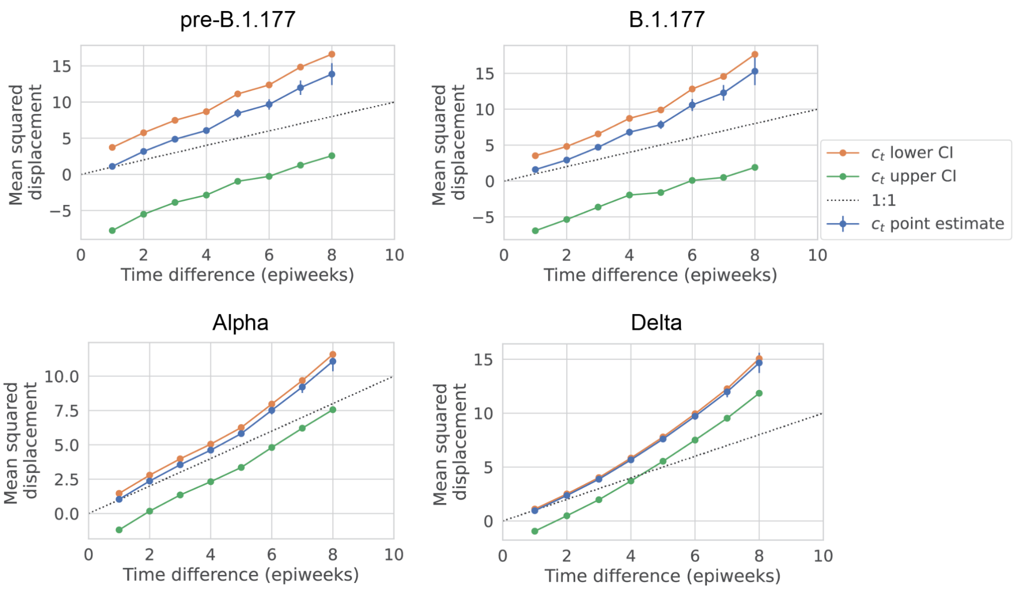

Figure R10: The mean squared displacement of the square root frequencies normalized by the inferred strength of measurement noise c_t (using the point estimate, upper confidence interval, or lower confidence interval), the number of sequences, and the inferred effective population size.

Figure R9 has been added to the manuscript as Supplementary Figure 14 and is referenced in line 225.

2. For removing spurious fluctuations that are actually due to selection, I don't understand the approach based on looking for lineages where one can reject a null hypothesis of neutrality. This would make sense if the goal of the paper were to identify lineages showing clear signs of selection for, e.g., follow-up on their characteristic mutations. But here what matters is how selection might bias estimates of drift, which is a different question. We know a priori that there are no lineages with a selective coefficient of exactly zero. I think it would make more sense to fit a model with each lineage having its own constant selective coefficient s_i that gets inferred along with all the other parameters. As long as most of the lineages are observed for multiple time points, it seems like it should be possible.

See above.

Reviewer #3: The following points should be addressed in a revision:

1. A point of concern is how the selection between major lineages could impact the inference method and the results. This applies specifically to well known clade displacements Alpha-Delta (May/June 2021), and Delta-Omicron, which happens just after the data cut-off on Dec. 2021. In both cases, the ancestral clade has census population sizes dropping very quickly, and how this affects the inference remains to be tested in simulations. A point to be noted is that the crossover from Alpha to Delta, which corresponds to a bottleneck in census population sizes, shows up in a plausible way in the inferred N_e. However, no trace of the next crossover is to be

seen in the reported N_e. The analysis should be extended at least to February 2022 to cover the full crossover from Delta to Omicron (BA.1).

We extended the analysis until March 25, 2022 (Figure R11). However, because the number of sequences sharply decreased in January 2022, the time points of the inference results that converged did not extend much further than the time period that was already in the main text. Additionally, there was a slight increase in the inferred effective population size at these later timepoints; we think this slight increase in the inferred effective population size may be due to the fact that we are averaging over 9 weeks and there is not enough data at the later times. When we redid the analysis with 5 weeks averaging rather than 9 weeks averaging, we saw a small dip in the inferred effective population size as expected, but this happened quite late and the too-small error bar suggests that there is not enough data. It is possible that our Delta estimates for this last month are not very reliable due to the limited data. On the other hand, another scenario that could be consistent is that the variance in offspring number decreased over time. We cannot disentangle both cases at the moment.

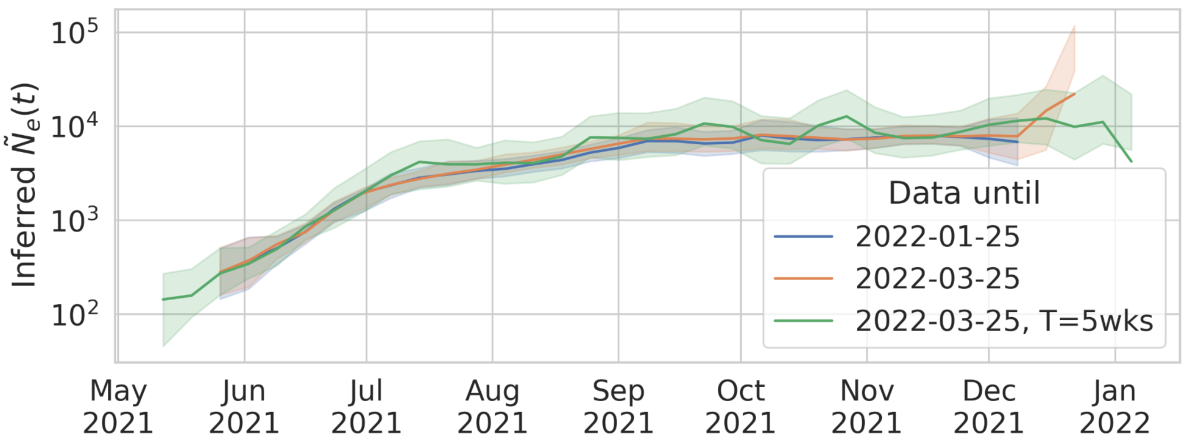

Figure R11: The inferred effective population size for Delta when extending the data until March 25, 2023, using a sliding time window of 9 weeks (default) or 5 weeks.

2. The authors limit their study on the COG-UK case-data because they provide "consistently large number of sequences SARS-CoV-2 cases". There is a number of other regions/countries with good sequencing and surveillance of SARS-CoV-2 cases over time (e.g., California, Germany, France). Is the method easily extendable to countries other than England? The manuscript makes a point on scalability and applicability of the method, so validation beyond one showcase scenario seems appropriate.

In the current version of the method, we created lineages from a tree, so the method is limited in application to datasets with a tree that includes most of the sequences or that has already classified sequences into lineages (for instance using a combination of making a tree with representative sequences, creating lineages, and then assigning new sequences to lineages

based on sequence similarity). We are currently working on extending our approach to use mutations, which would remove the need to make a tree, but in this first study, we defined lineages based on trees. This has been added to the discussion (lines 542-544).

3. Rare lineages that fall below the count/frequency threshold are randomly combined into "superlineages" that are above the threshold number. This may combine sequences that are far apart from each other on the tree instead of following topological structure. The procedure would be fine in a truly neutral evolution, but may affect a possible signal of selection. We would propose to combine rare lineages with their parent lineage instead. Also, the term "superlineage" is not very appropriate if the joined lineages are not closely related. Perhaps the term should be reconsidered anyway, because a reader could misunderstand it as suggesting higher fitness or super-spreading. Maybe ``coarse-grained lineages'' is more appropriate?

We were unsure of how to combine lineages with their parent lineages, but we did do an analysis that we think should address the reviewer's point that combining closely related lineages into coarse-grained lineages may lead to different results. We cut the tree closer to the root, which should group closely related lineages together (Figure R12-13). The inferred effective population size from these new groupings is slightly different from the results of randomly combining lineages, but not drastically so (still within the error bars) (Figure R12). The slight difference may be because of the reviewer's point that there are some slightly non-neutral dynamics in the population, which we also observed in our detection of non-neutral lineages.

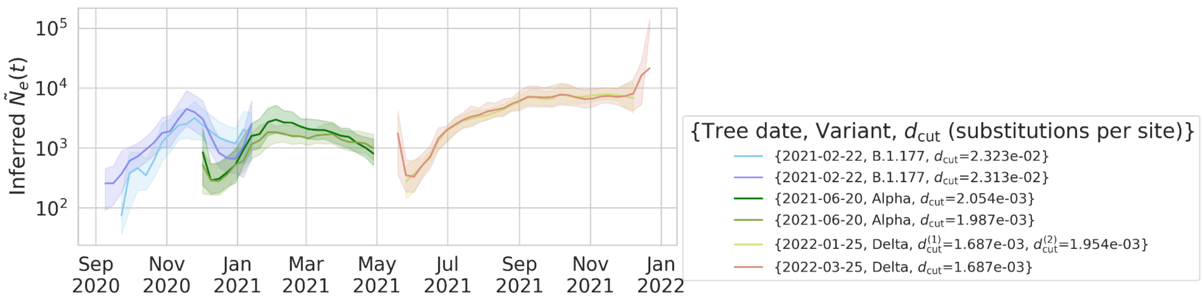

Figure R12: The inferred effective population size when cutting the tree at different depths to test the effect of combining lineages with other more closely related lineages in forming the coarse-grained lineages.

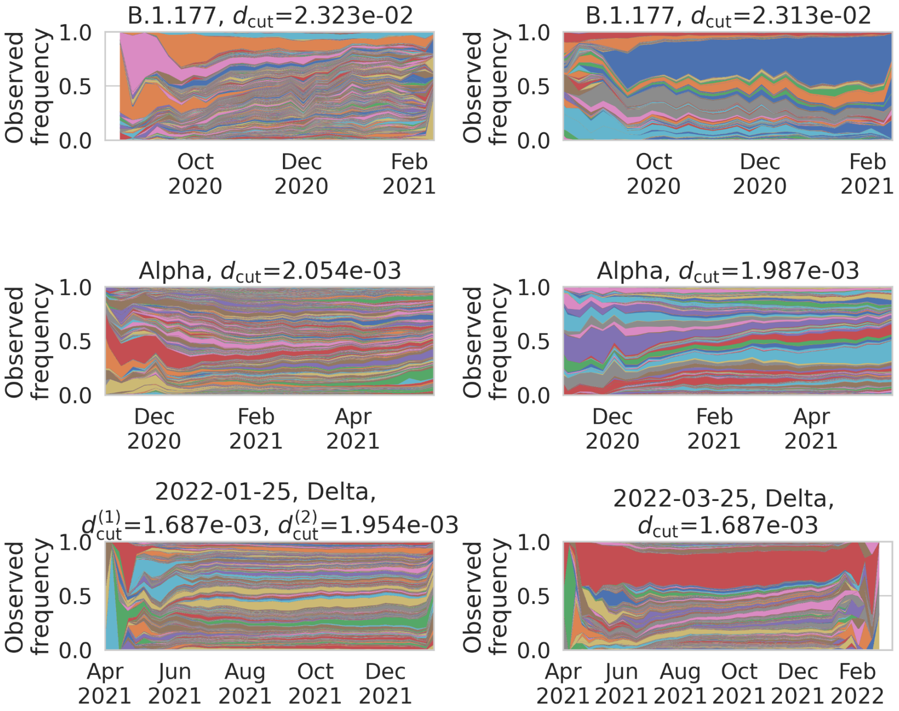

Figure R13: The lineage frequency time series using the tree cut depths shown in Figure R12.

These figures have been added as Supplementary Figures 10 and 11 and referenced in line 214.

The term “superlineages” has been changed to “coarse-grained lineages” throughout the manuscript as suggested.

4. The following is not a ``major issue'' but a suggestion on how to increase the impact of the paper.
At the end of the day, what do we learn from the decomposition of noise into (broadly interpreted) genetic drift and measurement noise? This question deserves more quantitative comments. The authors mention the origination probability for new mutant lineages, which is clearly an interesting point for application of the results. But what determines the origination and fixation probability of a new variant just observed in a few counts? If both types of noise enter (apart from the, say, positive selection coefficient of the variant), does the decomposition matter?

The strength of genetic drift influences the effective population size and says something about the population dynamics (i.e. a not well-mixed population of a given size) and its demography. The decomposition of noise into genetic drift and measurement noise matters because the establishment probability is only affected by genetic drift but not (our inferred) measurement noise. The ultimate fixation probability depends on the strength of genetic drift and as well as

the competition between simultaneously sweeping lineages (clonal interference) and thus it is only partially controlled by the strength of genetic drift. This has been added in lines 308-337.

Similarly, could the results on N_e say something on the effectivity of NPI measures and viral control strategies?

If our interpretation is correct that deme structure and jackpot events strongly influence the effective population size, then superspreading *events* are important to decrease and NPI measures should try to reduce these superspreading events. This has been added in lines 467-471.

Part III – Minor Issues: Editorial and Data Presentation Modifications
Please use this section for editorial suggestions as well as relatively minor modifications of existing data that would enhance clarity.

Reviewer #1: n/a

Reviewer #2: # Minor comments/questions

1. It looks like the estimate of the variance from [44] is pretty consistent with this paper's estimate. This should be highlighted more. (Or clarified if I'm misunderstanding.) It would also be good if possible to compare to the result of [41].

Ref. [44] (Endo et al., 2020) has been highlighted in lines 420-422. Because this study models the overdispersion by fitting to the size distribution of outbreaks, their result may actually be consistent with ours in that the high variance in offspring number may be partly explained by superspreading events (for example, due to host deme structure) rather than only superspreaders.

Ref. [41] (Miller et al., 2020) finds that the fraction of infected individuals responsible for 80% of all secondary infections is between 0.02 and 0.1 and the basic reproduction number is between 2 and 3. Using the theory developed in Lloyd-Smith et a., Science, 2005, we converted this estimate to the dispersion parameter for a negative binomial offspring distribution, k, and the corresponding variance in offspring number (Figure R14) using gamma distributed individual reproductive numbers. We find that 0.02 to 0.1 corresponds to a variance in offspring number of between 65 and 627.5. These values are similar to the values that we estimate. However, we note that the data that was used was collected from February 23 to April 22, 2020, which is very early in the pandemic and before the start time of our analysis. This has been added to Table S1.

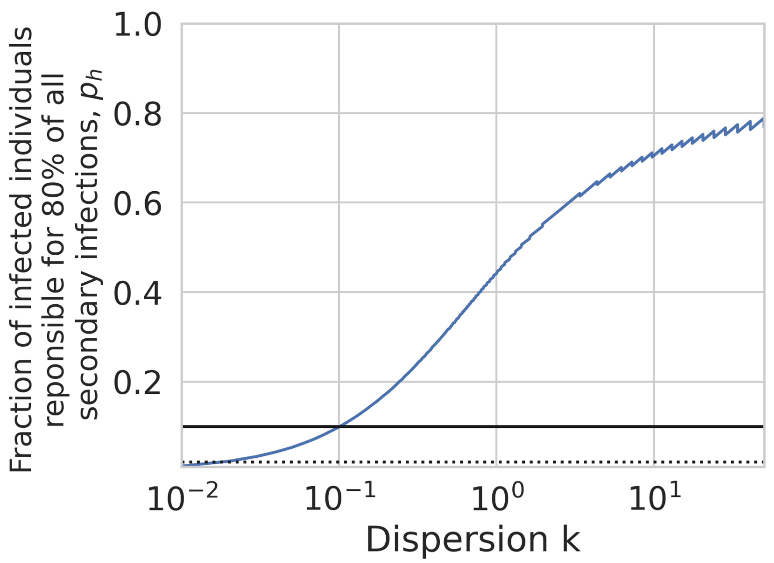

Figure R14: The dispersion parameter in a negative binomial offspring number distribution (corresponding to gamma distributed individual reproduction numbers) corresponding to the fraction of individuals responsible for 80% of all secondary infections, calculated using the approach from Lloyd-Smith et al., Science, 2005. The horizontal solid line indicates 0.1 and the horizontal dashed line indicates 0.02, which are the bounds of the estimates from Miller et al., Nature Communications, 2020.

2. I don't think the argument in 660-662 is correct, but as stated above I think this whole section should removed.

Yes, this section has been removed.

3. I think d_cut could be explained more clearly, and specifically that it's measuring a distance from the root. Often when people refer to "depth" in a tree they're talking about distance from the tips. I would also recommend a few words of explanation of the logic behind this choice of grouping.

The definition of d_cut in terms of distance from the root has been clarified in lines 611-612 and Figure S9 caption. The choice of grouping has been explained in lines 195-197 and 583-587.

4. Would the optimizer try to set c_t to be much less than 1 if it weren't constrained?

Because of the way the code is written, c_t cannot be less than 0 (the log of c_t is taken and then the value is later exponentiated). However, we did test what would happen if we allowed c_t to go to 0. In this case, the optimizer does indeed set c_t to be between 0 and 1 sometimes (Figure R15); however, this does not have a large influence on the inferred c_t

at other timepoints as these inferred values are similar to what was shown in the main text (Figure 2d). Figure R15 has been added to the manuscript as Figure S28.

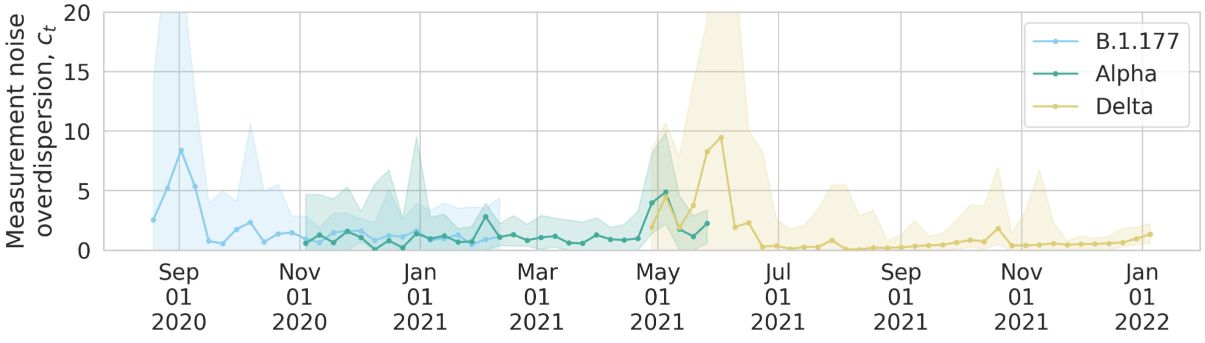

Figure R15: The inferred measurement noise overdispersion parameter when changing its lower bound from 1 to 0.

5. 543: why allow $\tilde N_e$ values up to 10^{11} if all answers that are $> 10^5$ are going to be thrown away? Just to be clear, the runs with $\tilde N_e > 10^5$ have much lower likelihood than the convergent runs, right? Would including the higher final values make much difference in the estimates?

This was a leftover choice from an older version of the analysis. We had previously noticed that runs with inferred $\tilde N_e$ above 10^5 are at 10^{11} indicating that it did not converge. Thus, we had set the threshold at $\tilde N_e$=10^5. We checked again for the present analysis, and this still seems to be the case (Figure R16). Thus, neglecting values larger than 10^5 effectively only neglects the runs that did not converge. Figure R16 has been added to the manuscript as Figure S27.

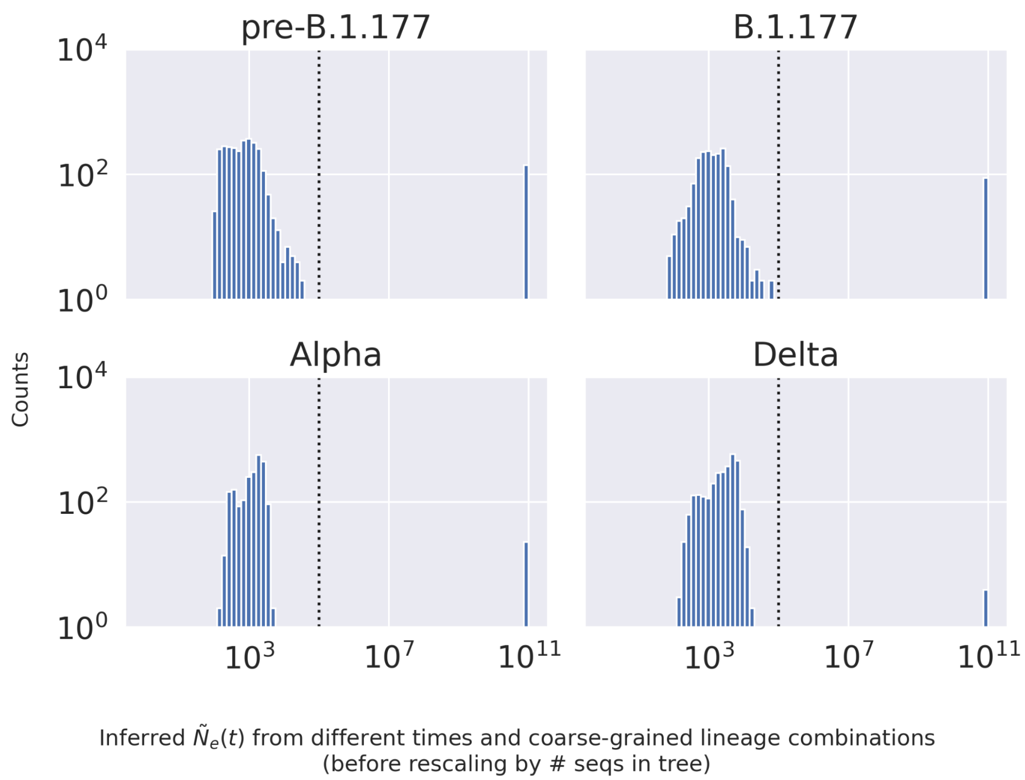

Figure R16: Inferred effective population size from different times and coarse-grained lineage combinations. The vertical dashed line indicates 10^5 which is the value above which results in the text were thrown away due to non-convergence (these only include values at 10^{11}).

Typos etc

- 111-114: repetitive with 84-86

84-86 has been shortened to not be repetitive.

- 183-184: better to say "only one or a few pangolin lineages"

This has been updated.

- I didn't understand 188-189, "any downsampling was done by preserving genetic diversity".

This has been clarified in the text (now lines 193-195).

- Fig 2a: "Lineage 1" cut off

In our version of the pdf, the label was not cut off.

- 222: "anda cross" -> "and across"

This has been updated.

- Indicate the super-spreaders in Fig 3a with a different color?

The superspreaders have been updated to be blue rather than red.

- Fig 3b and accompanying text (269-278) a bit off. First, text refers to "3c" rather than 3b. Also states that if every contact leads to a transmission, it gives a narrow offspring distribution with no superspreading, but this is wrong: the index case in each deme has many offspring/is a superspreader, with all the others having 0 or 1 offspring (because everybody else in their deme will already be infected). The basic idea is right though. Maybe just cut the part about every contact leading to a transmission event.

These changes have been made.

- Fig S18: show d_cut for Alpha?

We didn't use this tree for the main text (we used the other Alpha tree), which is why it did not show a dashed horizontal line, but we have added dashed lines for the two values of d_cut that were tested in Supplementary Figure S9 (Figure R17). The corresponding figure in the manuscript (Figure S25) has been updated.

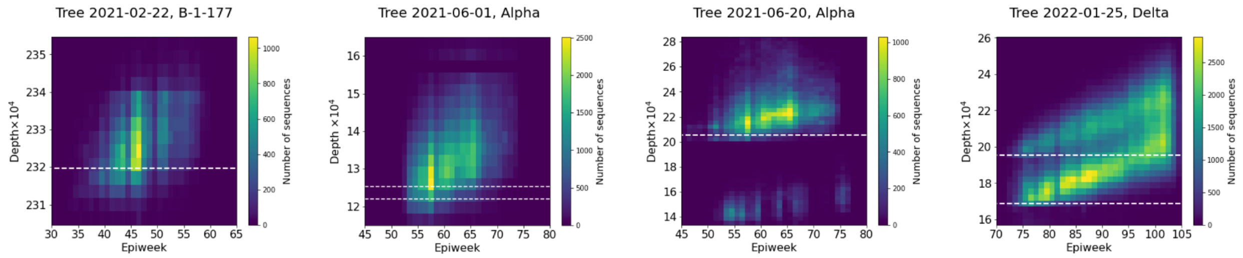

Figure R17: Update to Figure S25 in the text showing the dashed white lines in the second panel for the values of d_cut that were used in Figure S9.

- 489, 496: "$0 \ll f$" doesn't mean anything. Want $1/\tilde N_e \ll f$, right?

Yes, good point. It has been updated in line 639.

- 502: "number of counts is sufficiently large": like 25? Better to be a bit more specific.

The number of counts needs to be large enough such that the central limit theorem applies, so above approximately 20. This has been updated in the manuscript.

- 483: mention at the beginning that you're introducing the approximations to get a nice form for the likelihood. Otherwise it's not clear at first why the exact expressions aren't being used.

This has been updated in lines 635-637.

- 561-564: should explain better what the issue here is---the omitted sequences may be biased towards low fitness, right? Also, what fraction of the sequences are we talking about here? Some numbers would be useful to get a sense of whether this is a technicality or a serious issue.

The omitted sequences could bias the effective population size even in neutral dynamics, if a particular part of the tree is being left out. The fraction of sequences assigned to a lineage is shown in Figure R18 and has been added to the manuscript as Figure S29. This has been clarified in lines 732-734.

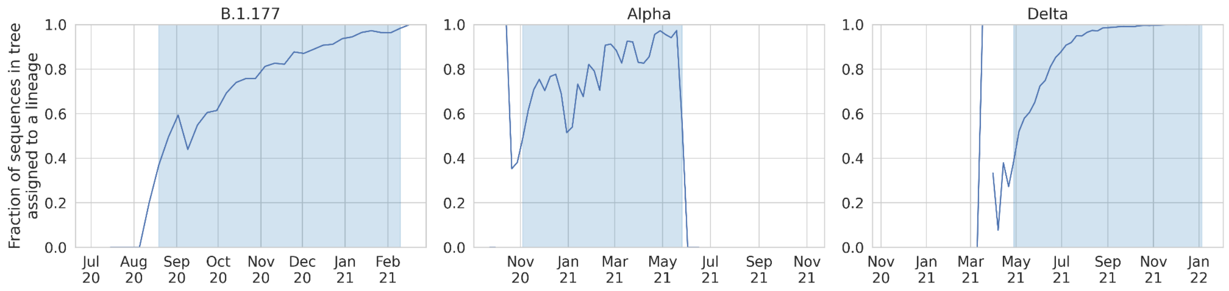

Figure R18: The fraction of sequences in the tree that are assigned to a lineage. The blue shading indicates the period of time in the data that was used for the inference analysis.

- 670: "emmission"

Thank you; this has been updated.

Reviewer #3: 5. The authors map the effective population size as a function of time, both for whole England and for each region within England. They find evidence that transmission was not well mixed at that time. These type of finding should be strongly dependent on the NPI (non-pharmaceutical intervention) measures that restrict movement (regionally/internationally). Could you relate changes in the inferred effective population size with changes in the national set of travel restrictions or measures? And does it differ from the signal in the census population sizes? This could help the interpretation of the results.

Thank you for this comment. We were also very interested in mapping our results to timings of lockdowns and changing restrictions, but the changes in the ratio of census to effective population size over time were comparable to the size of the error bars, so we could not conclude anything definitive about changes over time, with the one exception that is discussed in the manuscript for Alpha.

Regardless, out of curiosity, we did look into the timings of lockdowns to see how they may have correlated with changes in the ratio of census to effective population size (disregarding the error bars), and we report these here. We do see a potential decrease in the ratio of the census and effective population sizes (roughly the variance in offspring number if no other evolutionary forces are at play) after each lockdown (https://www.instituteforgovernment.org.uk/data-visualisation/timeline-coronavirus-lockdowns) . The first lockdown began on March 26, 2020, and while we are unable to infer the effective population size this early on due to lack of data, we do see the ratio is slightly decreasing (although the error bars are large) until July 2020. The second lockdown began on November 5, 2020, and we see a slight decrease in the ratio for B.1.177 shortly after. The error bars for pre-B.1.177 are too large to be able to tell a trend. The third lockdown began on January 6, 2021, and we see a decrease in the ratio for Delta during this time. However, we expect the change in the effective population size and the ratio of the census and effective population sizes to depend in a complex way on NPIs (for instance, people will be following them to various degrees which may also change over time). We did feel that these observations were too speculative to include in the manuscript.

6. In simulations of different deme strucuture the authors vary the number of individuals within each deme and keep the number of demes constant. Should both quantities not been varied in a way such that the total population stays constant?

Indeed, both quantities should have been varied in a way such that the total population stays constant. However, since we only used Epiweeks 42-50 for the inference (when only a small number of demes have been infected), the total number of demes did not matter. Unfortunately, due to the computational costs of running these individual-based deme simulations, it was challenging to update the simulations.

7. The authors use a cutoff on the sum of frequency and population size on lineages? Is this additive criterion underpinned by a theoretical argument?

The cutoff examines both frequency and population size. There is not a theoretical argument, but this choice was mainly to account for the fact that the total number of counts varies over time. This has been added in lines 581-583.

Additional minor changes to fix typos:

- In equation 27, Ac (leftover notation from an earlier version of the manuscript) has been replaced by \kappa_{i,j}.

- All statements of “similar to a Kalman filter” have been replaced with “a Kalman filter” since we realized that our HMM is just a Kalman filter.

References

Bloom and Neher, Fitness effects of mutations to SARS-CoV-2 proteins, bioRxiv, 2023

Charlesworth et al., Effective population size and patterns of molecular evolution and variation, Nature Genetics Review, 2009

Charlesworth and Jensen, Effects of Selection at Linked Sites on Patterns of Genetic Variability, Annual Review of Ecology, Evolution, and Systematics, 2021

Dadonaite et al., A pseudovirus system enables deep mutational scanning of the full SARS-CoV-2 spike, Cell, 2023

Endo et al., Estimating the overdispersion in COVID-19 transmission using outbreak sizes outside China, Wellcome Open Research, 2020

Flynn et al., Systematic Analyses of the Resistance Potential of Drugs Targeting SARS-CoV-2 Main Protease, ACS Infectious Diseases, 2023

Hart et al., Inference of the SARS-CoV-2 generation time using UK household data, eLife, 2022

Iketani et al., Functional map of SARS-CoV-2 3CL protease reveals tolerant and immutable sites, Cell Host and Microbe, 2022

Lloyd-Smith et al., Superspreading and the effect of individual variation on disease emergence, Science, 2005

Miller et al., Full genome viral sequences inform patterns of SARS-CoV-2 spread into and within Israel, Nature Communications, 2020

Starr et al., Deep mutational scans for ACE2 binding, RBD expression, and antibody escape in the SARS-CoV-2 Omicron BA.1 and BA.2 receptor-binding domains, PLoS Pathogens, 2022
