# Supplementary material for: Lineage frequency time series reveal elevated levels of genetic drift in SARS-CoV-2 transmission in England
Source: PLoS Pathog. 2024 Apr 15;20(4):e1012090. doi: 10.1371/journal.ppat.1012090 (PMC11045146; doi:10.1371/journal.ppat.1012090)
Supplement: Attachment — Submitted filename: Response to Reviewers Round 2.pdf [file ppat.1012090.s039.pdf]

Reviewer's Responses to Questions

Part I - Summary
Please use this section to discuss strengths/weaknesses of study, novelty/significance, general execution and scholarship.

Reviewer #2: The authors have done a ton of work responding to reviews and have addressed my concerns. Fig R9/S14 looks great!

Thank you so much!

Reviewer #3: (No Response)

Part II – Major Issues: Key Experiments Required for Acceptance
Please use this section to detail the key new experiments or modifications of existing experiments that should be absolutely required to validate study conclusions.

Generally, there should be no more than 3 such required experiments or major modifications for a "Major Revision" recommendation. If more than 3 experiments are necessary to validate the study conclusions, then you are encouraged to recommend "Reject".

Reviewer #2: None

Reviewer #3: The paper presents an approach to separately infer the amount of genetic drift and measurement noise from genetic data. The main observation of the paper: the inferred effective population size (as defined in a Wright-Fisher model) is two orders of magnitude smaller than the number of positive cases. I appreciate the extensive work that has gone into the revision and the reply to reviewers' comments. Nevertheless, the revised version leaves a number of questions open.

1. The authors claim that the method is highly scalable because it only relies on lineage frequency time series data. However, in other parts of the paper, the authors do explicitly rely on the phylogenetic tree. This constraint also appears to preclude the application to data from different countries. At the same time, the authors find it difficult to combine smaller clades with their parent lineages, which should not be a problem when a tree is available. Additionally, the authors mention that they cannot extend the analysis to later times than March 2022 because there is not enough data, which precludes the analysis of later bottlenecks. Indeed, the total number of available sequences for UK decreases from >150.000 per month to ~20.000 per month, which should still be sufficient for the kind of analysis performed. Together, it remains unclear to what extent the method is scalable, what amount of data is necessary, and whether the method only relies (and should rely) on lineage frequency time series.

We thank the reviewer for these points.

Regarding “whether the method only relies (and should rely) on lineage frequency time series”, our method does only rely on lineage frequency time series, but our approach to getting the lineages was to cut a tree (where the tree was constructed using only the mutations and no time information) at a particular depth and group downstream sequences that stemmed from the same branch together into lineages. We chose this approach because there was a tree available for these sequences from COG-UK and we wanted to be somewhat consistent with the existing pango nomenclature for lineages, which were defined using a tree. In principle, we don’t need to use a tree to get lineages since we are just interested in tracking the frequency of groups of closely related sequences over time. Thus, another approach to get these groups of related sequences would be to perform clustering of sequences based on a distance metric without creating a tree. Another approach is to use allele frequencies (the frequencies of individual mutations) rather than lineage frequency trajectories. Both of these approaches have the potential to be more computationally scalable. Future work should evaluate the feasibility and accuracy of using these different approaches to process the data for inferring the effective population size. In the Discussion lines 455-469, we now describe more explicitly our motivation for how we created the lineages and provide an outlook on these different approaches to getting lineages, groups of sequences, or alleles:

Fourth, we have defined lineages by cutting the phylogenetic tree at a particular depth; we chose this approach because a tree available for these sequences from COG-UK and we wanted to be somewhat consistent with the existing pango nomenclature for SARS-CoV-2 lineages, which were defined using a tree. Recent advances have made building trees for large datasets more tractable, but we can potentially increase the scalability of our approach even further by making the method tree-free. For example, one idea is to cluster the sequences based on a distance metric and use cluster frequencies over time or another idea is to use allele frequencies (the frequencies of individual mutations). Future work should evaluate the feasibility and accuracy of using these different approaches to process the data for inferring the effective population size.

Regarding scalability, whereas our approach only needs a tree to define lineages, the most commonly used methods for inferring pathogen effective population size are phylodynamics methods (i.e. BEAST), which additionally require a time-scaled phylogeny and fitting a model to effective population size. As a result of these additional computationally-intensive steps, the phylodynamics methods are much less computationally scalable.

Regarding combining lineages with parent lineages, we are still not sure of what the reviewer means by this. If we are interpreting the reviewer correctly, the parent lineage that the reviewer refers to would be one that includes the lineage in question, so combining them together would be double counting the lineage in question. If the reviewer instead means combining the lineage with the other lineage that has the same parent, then we think that’s what we effectively did by cutting the tree closer to the root. The only difference would be that in this case, the cut depths would be different for each lineage, but we do not expect this to make a difference.

Another approach that we had thought of to address the reviewer's concern was to combine lineages based on genetic distance (i.e. a lineage that's smaller than the threshold size gets combined with the lineage that's closest to it in genetic distance). However, we quickly realized that given the number of lineages (for some variants, thousands), it was computationally too intensive to calculate the distance matrix. Thus, we settled on the approach of cutting closer to the root, which should achieve the same effect as combining lineages that are more closely related genetically.

Regarding the number of sequences available, from our data, we have 0 Delta sequences by March 1, 2022 (Figure R1). The sequences that the reviewer cites are probably of Omicron. We didn't look at Omicron because those sequences were not available at the time of our analysis. Additionally, there is more evidence of within-variant selection of Omicron, which may complicate the analysis and thus we believe it is beyond the scope of this paper.

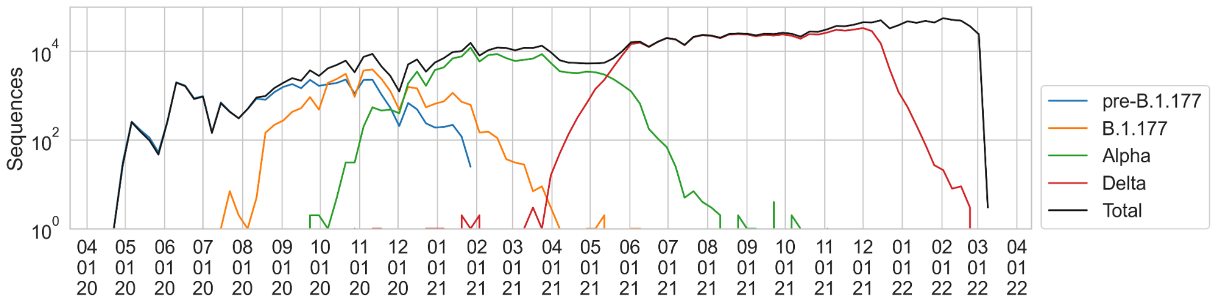

Figure R1: The number of sequences over time in the COG-UK pillar 2 data (filtered to only surveillance sequences) in total and stratified by different lineages and variants.

We believe that applying the approach to other datasets is possible but it is beyond the scope of the present proof-of-principle paper, in which we wanted to focus on the best sequenced region of the world (England). Extending our approach to other datasets is a very important future goal. Our simulations, which included many different parameter regimes, also point to the generalizability of our approach. We think that this approach would be best suited for large datasets with a long period of sampling, including HIV, Ebola, and maybe seasonal influenza. We have added this point to the Discussion in lines 471-473:

We think that this approach would be best suited for large datasets with a long period of sampling, and for pathogens this includes HIV, Ebola, and potentially seasonal influenza. It may also be interesting to adapt this approach to study data from field studies and ancient DNA.

2. The authors did not address the concern of how between-lineage selection affects the method of inference of effective population size. When a lineage is exponentially changing in size due to between-lineage selection, this could affect the inferred effective population size or its interpretation. Clearly, this point is related to the other referees' points on within-lineage

selection, e.g. purifying selection (which should be ubiquitous).

We would expect a lineage that is exponentially changing in size to also have approximately exponentially changing effective population size (as long as the other factors that affect N_e stay approximately the same, like the variance in offspring number or host network structure). We don’t think it would affect the interpretation of the inferred effective population size.

A bottleneck can be expected at the onset of a new wave when the new variant is still rarer. Indeed, our inferred effective population size reproduced these expected bottlenecks, between pre-B.1.177 and B.1.177, between B.1.177 and Alpha, and between Alpha and Delta. It is indeed interesting that the N_e of Delta stayed high even when it died out and Omicron emerged. When responding to the previous revision, our results led us to believe that it was because the number of sequences for Delta became too low. Following the current reviewer comment, we wanted to double check that this was the case. Indeed, when we now checked more closely, we saw that by Epiweek 108 (January 5, 2022) there were only 79 sequences of Delta. Because each coarse-grained lineage must have at least 20 counts, this meant that there were at most 3 coarse-grained lineages during this time, and this is not enough to infer the effective population size. We have added these points to the Discussion in lines 280-284:

Our approach was able to reproduce the expected decrease in effective population size during the decline of pre-B.1.177, B.1.177, and Alpha, as well as the increase in effective population size during the emergence of B.1.177, Alpha, and Delta (Figure 2c). We did not have enough sequences during the time when Delta was going extinct to infer the effective population size during that time period.

We addressed Reviewer 1’s point about within-lineage selection in our previous revision. We found that conservatively excluding lineages that could be non-neutral did not substantially change the inferred effective population size (Figure S18). Additionally, we used simulations and analytical theory together with the empirically estimated distribution of fitness effects (Figure S19) to test for the potential effects of background selection. We found that background selection could explain at most a factor of 2 reduction in the effective population size (Figure S20-22), which is only a small fraction of the observed reduction of two orders of magnitude. Thus, we concluded that within-lineage selection was not able to explain the reduced effective population size that we observed.

In summary, the manuscript presents a clever idea of noise decomposition to obtain estimates of the effective population size in a highly complex evolutionary system far from equilibrium. At the same time, some methodological questions remain open. More importantly, the discussion with the reviewers has shown that the evolutionary scenario of this system is, at least, sufficiently far away from textbook neutrality that the very meaning of the effective population size, regarded as an intermediate summary statistics for the computation of measurable quantities, remains to be clarified. What falsifiable results could you or others compute from the pattern of effective population sizes found, in order to ultimately corroborate or refute the contentious assumption of partial neutrality made in the inference procedure? For example, should we expect more nonsynonymous (slightly deleterious) mutations originating in the

bottleneck of June 2021 getting eventually fixed or not? How does the fixation probability of a mutation, in the scenario derived, depend on the sampled frequency of an actual observation? This type of questions is what I would hope any further revision of the paper could shed more light on.

We agree with the reviewer that the effective population size depends on many different factors, and we believe that we addressed the evolutionary forces that may influence the effective population size in our system (selection, mutation, and migration) in the previous round of revisions and found that they do not explain our results. We have updated the Discussion in lines 319-322 to include the following text:

Our method is not able to precisely pinpoint how many lineages are under selection, but it appears that there is relatively little within-variant selection in the time period we investigated, and our method is robust to slight deviations from neutrality.

We thank the reviewer for the opportunity to expand on the implications of our findings. The magnitude of the effective population size affects the establishment probability, or the probability that a new mutation will rise to a high enough frequency to escape stochastic extinction. The establishment probability of weakly beneficial mutations is linearly related to the effective population size (Kimura, 1962). For strongly beneficial mutations, the impact of the effective population size on the establishment probability is quantitatively less straightforward and depends on the host network structure (Alexander et al., 2010). It would be interesting to look at how the distribution of fitness effects of established mutations changes over time in a future paper and to compare it to the changes in effective population size that we observed, but we think it is beyond the scope of our current paper. In the absence of clonal interference, the fixation probability is the same as the establishment probability; if there is clonal interference, the fixation probability will depend on additional factors like the mutation rate (Schiffels et al., 2011; Good et al., 2012). The low effective population sizes that we observe suggest low establishment probabilities, and new variants may have to arise several times before one of them makes it. The sampling or observation process should not affect the fixation probability. We have now added these points to the Discussion in lines 325-338:

Accurately estimating the strength of genetic drift allows us to better understand disease spread and extinction, as well as to better parameterize evolutionary models and understand how mutations will establish in the population. The establishment probability is the probability that a new mutation will rise to a high enough frequency to escape stochastic extinction. For weakly beneficial mutations, the establishment probability is linearly related to the effective population size [52]. For strongly beneficial mutations, the impact of the effective population size on the establishment probability is quantitatively less straightforward and depends on the host network structure [3]. In the absence of clonal interference, the fixation probability, or the probability that the mutation will fix in a population, is the same as the establishment probability; if there is clonal interference, the fixation probability will depend on additional factors like the mutation rate [53, 54]. The low effective population sizes that we observe suggest low establishment probabilities; the probability that any newly arisen beneficial mutant rises to a significant frequency will be small. and new variants may have to arise several times before one of them

makes it. More generally, our results give an order of magnitude estimate for the effective population sizes that can be used to more accurately parameterize evolutionary models for SARS-CoV-2 as well as an approach to infer the effective population size in more specific contexts.

In summary, we have determined one crucial parameter for evolutionary modeling and forecasting - the effective population size. Our results give an order of magnitude estimate for the effective population sizes that can be used in future forecasting models for SARS-CoV-2, which will affect the stochastic dynamics of the predicted evolutionary trajectories and the establishment probabilities.

Part III – Minor Issues: Editorial and Data Presentation Modifications
Please use this section for editorial suggestions as well as relatively minor modifications of existing data that would enhance clarity.

Reviewer #2: The one thing I would say is that I think Fig R10 is a very interesting result as well and would recommend also including it in the manuscript. But I can also see the argument that this manuscript is already turning into a beast, and maybe Fig R10 is sufficiently interesting that it's worth following up on in its own separate work rather than getting lost in this one.

Thank you for this suggestion! We agree that it is an interesting result. After discussing, we have decided that Fig R10 is something we would like to follow up on in a separate work so we have not included it in the manuscript.

Reviewer #3: (No Response)

References

Alexander and Day, Risk factors for the evolutionary emergence of pathogens, J. R. Soc. Interface, 2010

Good et al., Distribution of fixed beneficial mutations and the rate of adaptation in asexual populations, PNAS, 2012

Kimura, On the probability of fixation of mutant genes in a population, Genetics, 1962

Schiffels et al., Emergent Neutrality in Adaptive Asexual Evolution, Genetics, 2011
